# Controlling the shell growth mechanism and interfacial oxidation of InAs/ZnSe quantum dots for shortwave infrared emission

**DOI:** 10.1038/s41467-026-77896-0

**Published:** 2026-10-19

**Authors:** Taewan Kim, Manuela De Franco, Miguel Dosil, Jae Taek Oh, Rajesh Bera, Marta Martos Valverde, Gaurav Kumar, Gerasimos Konstantatos

**Affiliations:** 1 ICFO–Institut de Ciències Fotòniques, The Barcelona Institute of Science and Technology, Castelldefels, Barcelona, 08860 Spain; 2 ICREA–Institució Catalana de Recerca i Estudis Avançats, Passeig Lluís Companys 23, Barcelona, 08010 Spain

**Keywords:** Quantum dots, Materials for devices, Lasers, LEDs and light sources

## Abstract

Short-wave infrared (SWIR) emitters underpin applications in communication, imaging and sensing, yet efficient, scalable devices remain limited. Colloidal InAs quantum dots (InAs CQDs) offer a compelling, cost-effective alternative to conventional epitaxial SWIR light-emitting diodes (LEDs). However, developing heavy-metal-free InAs CQDs for emission beyond 1400 nm is hindered by lattice mismatch during core/shell growth, which degrades photoluminescence quantum yield (PLQY). Furthermore, integrating CQDs into LEDs often introduces severe interfacial quenching and intrinsic charge imbalances. Here we report a highly efficient heavy-metal-free SWIR LED operating at 1525 nm, enabled by tailored injection strategies and oxidation-induced interfacial engineering. Optimized high-temperature ZnCl₂ treatment and continuous precursor injection improve shell nucleation and growth, while controlled interfacial oxidation is associated with reduced shell-thickness-dependent strain signatures and improved PLQY retention during shell thickening. Together, these strategies yield measured PLQYs exceeding 55% for emission beyond 1400 nm. Moreover, we demonstrate that incorporating a semiconducting InAs CQD interlayer effectively suppresses interfacial quenching and regulates electron over-injection. Combined with a doped hole-transport layer to reduce series resistance, our optimized devices achieve a maximum external quantum efficiency of 3.5% and a record radiance of ~17 W sr^−1^ m^−2^, alongside a T50 operational lifetime of 16 h. Our findings suggest that integrating mechanism-driven shell growth with rational device engineering offers a strategy to overcome current bottlenecks in colloidal emitters, facilitating the transition toward short-wave infrared applications.

## Abstract (web summary)

Short-wave infrared emitters could support communication, imaging and sensing, but efficient, scalable devices remain elusive. Kim, De Franco et al. engineered indium arsenide quantum dots and device interfaces, achieving efficient emission, high radiance and improved stability.

## Introduction

Short-wave infrared (SWIR) light sources are essential for emerging technologies, including advanced optical communication^1^, deep-tissue bio-imaging^2^, and machine vision^3^. However, conventional epitaxial SWIR light-emitting diodes (LEDs) face critical bottlenecks, including strict lattice-matching requirements and high-cost manufacturing processes, hindering integration with silicon. Colloidal quantum dots (CQDs) present a compelling solution to these challenges. With their size-tunable bandgap, direct band transitions, and narrow emission linewidths, CQDs not only offer superior optical properties but also enable cost-effective solution processing and substrate-independent integration. Despite this potential, SWIR CQD LEDs remain in their early stages. Previous studies on SWIR CQD LEDs have relied on toxic heavy metal-based semiconductors^4–6^, with the exploration of heavy-metal-free SWIR candidates being largely uncharted.

InAs CQDs are emerging as promising lead-free semiconductor nanocrystals for applications in the NIR and SWIR regions, owing to their excellent electrical conductivity, high thermal stability, and broad optical wavelength tunability^7,8^. Early demonstrations of InAs CQD electroluminescence were achieved at around 1200 nm, but reported external quantum efficiency (EQE) was below 0.5%^9^. Since the demonstration of InAs CQDs with absorption beyond 1600 nm in 2021^10^, substantial progress has been made in developing more scalable and accessible synthetic routes. In particular, conventional syntheses rely on highly reactive tris(trimethylsilyl)arsine (TMS-As)^11^ precursors, which are hazardous and subject to strict handling and regulatory limitations, motivating the development of safer alternatives such as tris(dimethylamino)arsine (amino-As)^12–14^. Despite these advances, achieving high photoluminescence quantum yield (PLQY) in SWIR-emitting InAs CQDs remains challenging due to non-radiative recombination from surface defects, interfacial strain, and incomplete passivation, reflecting a limited mechanistic understanding of interface formation and surface passivation. To date, relatively high PLQY has been demonstrated primarily for core/shell InAs CQDs emitting in the 950-1100 nm range^15^, while emission beyond 1400 nm typically yields PLQY below 20%^16^.

Recent progress in amino-As-derived InAs/ZnSe core/shell CQDs has enabled RoHS-compliant CQD LEDs with steadily improving performance. EQEs have increased from 5.5% at 947 nm to 13.3% at 896 nm through shell optimization^17,18^. However, these advances rely on relatively small CQDs, limiting emission to wavelengths below 1100 nm. Extending emission into the SWIR regime, LEDs based on larger InAs/ZnSe CQDs achieve EQE of 1.1% at 1410 nm, representing the state of the art for InAs-based CQD LEDs beyond 1.4 μm to the best of our knowledge^19^. Nevertheless, both PLQY and device efficiency remain limited at longer wavelengths.

One widely used approach to improve the PLQY of core-shell structures is growing multiple or graded shell to mitigate lattice mismatch^20,21^. However, these methods increase the total particle size, hindering charge injection in light-emitting diodes, and require complex multi-step synthetic processes^22^. Another intriguing yet contentious approach to increasing the PLQY of III-V InP core/shell CQDs is the oxidation of the core. While oxidation of the CQD surface is generally known to increase the full width at half maximum (FWHM) and create traps that decrease the PL and device performance^23^, it has also been shown to increase the PLQY of InP cores by reducing lattice mismatch with ZnS shell^24^ or passivating surface defects^25^. Nevertheless, the precise structural role of the oxide layer, particularly whether it functions merely as a surface passivator or as a structural buffer to facilitate epitaxial growth, has not been fully elucidated^23–25^.

In this study, we report an InAs/ZnSe core/shell CQD synthesis strategy achieving a PLQY over 40% at wavelengths beyond 1400 nm, avoiding highly reactive reducing agents or toxic precursors. This performance is enabled by an optimized shell growth method and an oxide layer. Specifically, high-temperature ZnCl_2_ treatment and continuous precursor injection improve shell growth, while controlled interfacial oxidation is associated with reduced shell-thickness-dependent strain evolution and improved PLQY retention. Together, these strategies yield measured PLQYs exceeding 55% at emission wavelengths beyond 1400 nm. To demonstrate the full potential of our highly emissive CQDs, we fabricated SWIR LEDs targeting 1550 nm emission. To maximize device efficiency and radiance, we engineered the charge-transport interfaces by introducing a small-sized InAs CQD layer as an electron-transport interlayer, together with a doped hole-transport layer. The InAs CQD interlayer effectively suppresses non-radiative recombination at the interface, thereby prolonging the radiative recombination lifetime and reducing trap-mediated losses. Concurrently, the use of p-doped poly-[bis(4-phenyl)(2,4,6-trimethylphenyl)]amine (PTAA) enhances hole transport, ensuring higher radiance. These synergistic interfacial improvements yielded a record-setting EQE of 3.5% and a radiance of 17 W sr^−1^ m^−2^, representing the highest performance reported to date for SWIR InAs CQD LEDs.

## Results and discussion

### Growth mechanism elucidation and optimization for InAs/ZnSe Core/shells

**Fig. 1:**
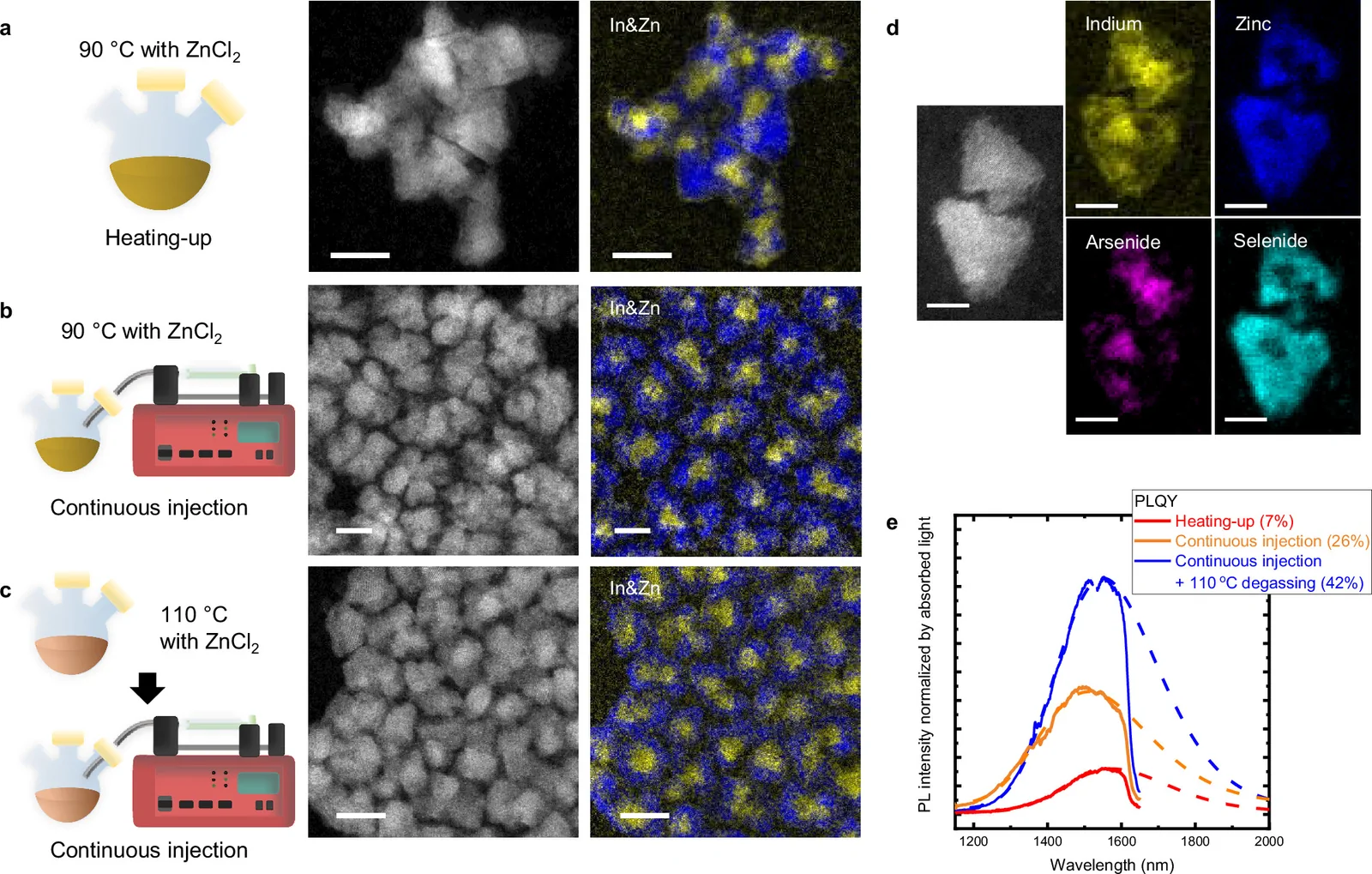
Elucidation of the InAs/ZnSe core/shell growth mechanism. **a** HAADF-STEM image and corresponding EELS elemental map (In, yellow; Zn, blue) of InAs/ZnSe core/shell CQDs synthesized via a conventional heating-up method with ZnCl₂ degassed at 90 °C, showing severe aggregation. **b** HAADF-STEM image and EELS elemental map of core/shell CQDs synthesized under identical ZnCl₂ degassing conditions but with a continuous precursor injection protocol, revealing a pinwheel-like shell morphology with non-conformal ZnSe growth. **c** HAADF-STEM image and EELS elemental map of core/shell CQDs synthesized with ZnCl₂ degassed at 110 °C followed by continuous injection, yielding uniform tetrahedral or near-spherical morphologies. The white scale bars in Fig. 1a–c correspond to 10 nm. **d** EELS elemental maps of a representative CQD from the optimized synthesis (c), showing the spatial distribution of indium, zinc, arsenic, and selenium, with trace co-distribution of In and As in the shell region indicative of interfacial alloy formation. The white scale bars in Fig. 1d correspond to 5 nm. **e** PL spectra of InAs/ZnSe core/shell CQDs synthesized under the three conditions, with PLQY values of 7%, 26%, and 42% for heating-up, continuous injection, and 110 °C degassing with continuous injection, respectively.

In general, InAs CQDs synthesized using amino-As precursors form tetrahedral shapes composed of In-rich (111) facets. The In-rich (111) facets can alloy with shell precursors such as Zn, effectively mitigating lattice mismatch and enabling uniform shell growth and surface passivation in small-sized CQDs^26^. However, the enlarged flat facets in larger CQDs frequently experience increased lattice mismatch at the core-shell interface^27–29^. As a result, shell growth on these surfaces becomes less uniform and more prone to the formation of interfacial defects, severely degrading the PLQY. To investigate this structural degradation at the interface, we first analyzed the InAs/ZnSe core/shell structures grown via conventional heating-up methods. InAs CQDs were synthesized using In(I)Cl and amino-As precursors, following a modified protocol from a reported method^12^. Although the use of In(I)Cl provides a scalable synthesis route for large-sized SWIR InAs CQDs, the previously reported one-pot InAs/ZnSe synthesis resulted in aggregation. Therefore, a 20-fold molar ratio of ZnCl_2_ relative to In was degassed with the cores at 90 °C, followed by the injection of an equivalent molar amount of TOP-Se relative to ZnCl_2_ prior to the heating-up process. Figure 1a shows the electron energy loss spectroscopy (EELS) elemental mapping of In and Zn for these baseline samples. As observed in the transmission electron microscopy (TEM) images, the synthesized core/shells exhibited significant aggregation.

Hypothesizing that this aggregation was caused by an excessive amount of shell precursors, we conducted the heating-up process with only half the initial amount of TOP-Se, followed by the continuous injection of the remaining half. Figure 1b presents the TEM images and EELS mapping of In and Zn for the core/shells synthesized using this approach. While the aggregation of the CQDs was significantly reduced, we observed that the shell did not coat the core uniformly, but rather grew into a pinwheel-like morphology. To understand the origin of this phenomenon, we performed TEM with EELS measurements on the core/shells at the initial stage of the reaction (Supplementary Fig. 1). Zn-rich domains were preferentially localized near the vertices of the InAs cores. In the corresponding high-resolution images, the lattice fringes within these Zn-rich regions were non-collinear with those of the adjacent InAs core, demonstrating that the initial domains were crystallographically misoriented rather than conformally extending the core lattice. These observations support a vertex-initiated island-nucleation pathway. The occurrence of approximately four to five wing-like domains in the fully grown particles is also consistent with the growth of multiple independently nucleated domains.

The degassing of ZnCl_2_ was initially performed at 90 °C because ZnCl_2_ can etch the surface of InAs CQDs. However, we found that deliberately inducing this etching process results in structural modifications. Figure 1c displays the TEM and EELS mapping of In and Zn for InAs/ZnSe core/shells synthesized from InAs cores treated under vacuum at 110 °C for 2 h with continuous injection process. Unlike the previous cases, these core/shells predominantly exhibited a tetrahedral or sphere-like morphology. To understand the mechanism behind this structural evolution, EELS mapping including In, As, Zn, and Se elements was examined. In contrast to the 90 °C case, which provided separated InAs and ZnSe as shown in Supplementary Fig. 2, trace amounts of In and As were found to co-distribute with Zn and Se in the shell region in Fig. 1d. This observation suggests that the InAs etched by ZnCl_2_ re-participates in the reaction during shell growth, leading to the formation of an alloy structure. As shown in Fig. 1e, while the initially aggregated core/shells and the pinwheel-shaped shells exhibited PLQYs of only 7% and 26%, respectively, the core/shells synthesized under the optimum conditions achieved an efficiency of approximately 42%.

One notable observation, consistent with previous studies on similar architectures^26^ and our TEM data (Fig. 1d and Supplementary Fig. 3), is that the ZnSe shell does not conformally coat the InAs core. Instead, it often grows as an inverted tetrahedron relative to the core, with the core vertices facing the shell’s facet centers rather than its vertices. In addition, although the ZnSe-rich domains initially nucleate without lattice matching at the core vertices, the fully grown core/shell particles exhibit epitaxial registry across the InAs/ZnSe interface. We speculate that this behavior arises from strain-driven island nucleation of ZnSe on specific InAs (111) facets, followed by the subsequent growth of new tetrahedral structures to minimize the overall interfacial energy and lattice mismatch, as shown in Supplementary Fig. 3. This non-uniform growth mode introduces two significant structural challenges. First, forming a direct epitaxial bond between these zinc-blende facets induces a severe charge imbalance. Unlike uniform growth, where the charge mismatch between In (+¾) and Se (- ½) generates a minor imbalance of only ¼, interfacing the In (111) and Se (111) facets creates a charge disparity (+^3^/_4_ vs. −^3^/_2_) as shown in Supplementary Fig. 3. This charge mismatch is expected to lead to the formation of dangling bonds or a highly defective amorphous interface to bridge the gap. Second, as these isolated ZnSe tetrahedra grow on different facets and eventually coalesce, the accumulated lattice distortion and strain at their merging boundaries likely hinder the formation of a continuous, defect-free crystal shell. Although our modified shell growth process partially mitigates these growth non-uniformities, the fundamental challenges of lattice mismatch and interfacial strain remain.

### Role of the interfacial oxidation in InAs/ZnSe core-shell structures

To address the challenge of shell growth on these enlarged facets, we explored a surface-engineering strategy involving controlled air exposure. To evaluate the effect of core oxidation on subsequent shell growth, 1400 nm InAs cores were exposed to ambient air at room temperature for 0–144 h after purification. As 3 *d* XPS analysis showed a progressive increase in the relative contribution of oxidized arsenic species with increasing exposure time (Supplementary Fig. 4). The resulting InAs/ZnSe core/shell CQDs exhibited their highest PLQYs of approximately 40–45% when the oxidized arsenic contribution was in the range of approximately 17–26%. Based on these results, an oxidation time of 12 h was selected as the standard condition. Detailed experimental conditions are provided in the Methods section.

**Fig. 2:**
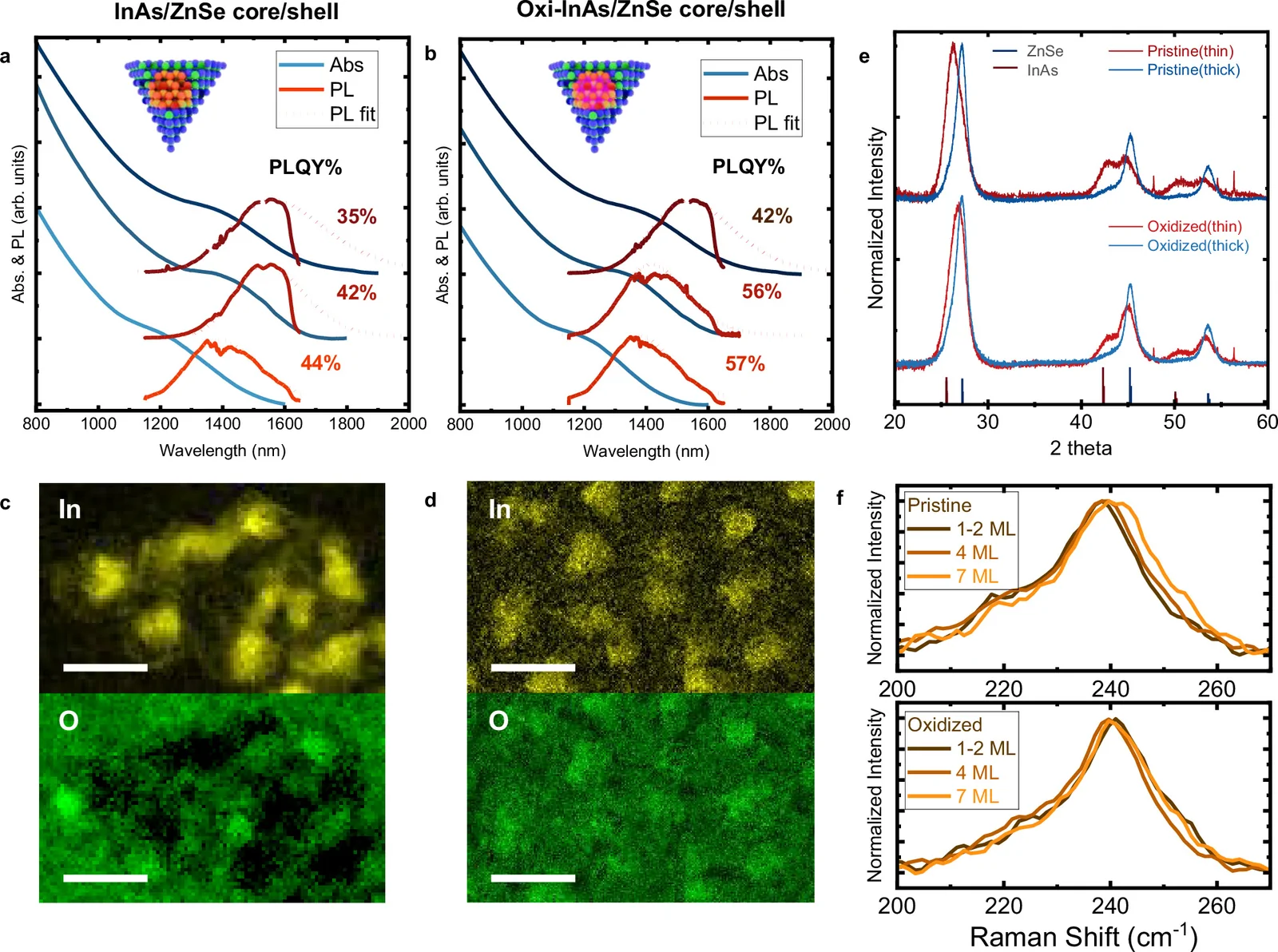
Optical characterization and structural analysis for oxidation-associated strain evolution in InAs/ZnSe core/shell CQDs. **a, b** Absorbance and PL spectra of pristine (**a**) and oxidized (**b**) InAs/ZnSe core/shell CQDs synthesized from cores with varying bandgaps. Dashed lines indicate fitted PL spectra. The corresponding PLQY values are indicated for each sample. **c, d** EELS elemental maps of indium (In, yellow) and oxygen (O, green) for pristine (**c**) and oxidized (**d**) InAs/ZnSe core/shell CQDs. The oxygen background present in both samples arises from methanol used during TEM sample preparation. The white scale bars in Fig. 2c and d correspond to 10 nm. **e** XRD patterns of thin- and thick-shell InAs/ZnSe CQDs synthesized from pristine and oxidized cores. Reference peak positions for bulk ZnSe (blue) and InAs (red) are indicated. **f** Raman spectra of pristine (top) and oxidized (bottom) core/shell CQDs with varying ZnSe shell thicknesses (1–2, 4, and 7 monolayers).

Figure 2a displays absorption and photoluminescence spectra of the resulting InAs/ZnSe core/shell CQDs synthesized from cores with varying bandgaps. The pristine samples, which were synthesized under the optimized condition of 110 °C ZnCl_2_ treatment and continuous precursor injection, exhibited PLQYs of 44%, 42%, and 35% across the 1300 to 1500 nm range, which outperform previously reported values for InAs-based core/shell CQDs in this wavelength range. Furthermore, the oxidized counterparts demonstrated further enhanced PLQYs of 57%, 56% and 42% in the 1200 to 1500 nm window in Fig. 2b, respectively. The batch-to-batch reproducibility of the optimized synthesis was evaluated using six independently synthesized core/shell CQD batches, yielding a mean PLQY of approximately 39%. The optical properties of the pristine and oxidized InAs/ZnSe core/shell CQDs are summarized in Table 1.

**Table 1:** Optical properties of pristine and oxidized InAs/ZnSe CQDs

InAs/ZnSe	1st peak of core (nm)	Emission peak (nm)	PLQY (%)	FWHM (meV)
Pristine	1254	1380	44 ± 3	165
	1294	1559	42 ± 3	137
	1392	1591	35 ± 2	180
Oxidized	1220	1363	57 ± 4	151
	1238	1416	56 ± 4	149
	1367	1560	42 ± 3	161

To investigate the role of interfacial oxidation, we synthesized core/shell CQDs with a thin shell layer and conducted EELS compositional analysis. Both the pristine and oxidized-core samples exhibited high initial PLQYs of 44% and 42%, respectively (Supplementary Fig. 5a and b). As shown in Fig. 2c, the EELS mapping of the pristine InAs/ZnSe CQDs reveals a distinct indium signal but lacks any localized oxygen signal in the CQDs, as shown in Supplementary Fig. 6. In contrast, the EELS mapping of indium and oxygen for the oxidized-core/shell CQDs in Fig. 2d reveals a localized enhancement of the oxygen signal at positions coinciding with the In-rich region, which is absent in the pristine sample (Supplementary Fig. 7). Although a high oxygen background signal is present in both samples due to the methanol treatment during TEM sample preparation as shown in Supplementary Figs. 6 and 7, the compositional profile clearly distinguishes this localized interfacial oxide layer, which is absent in the pristine sample.

To closely investigate the role of the interfacial oxide layer in the growth of core/shell CQDs, we analyzed samples with varying shell thicknesses. While the oxidized core/shell structures maintained a PLQY of approximately 40% even with increased shell thickness, the PLQY of the thick pristine core/shell structures decreased to 23% as shown in Supplementary Fig 8. To analyze this, X-ray diffraction measurements (XRD) revealed that for thin shells, the oxidized sample exhibited peaks closer to zinc selenide, whereas thick shells showed no significant difference between the two samples (Fig. 2e). To verify whether these effects originated from variations in the shell thickness, TEM images and absorption spectra were compared. Given the absence of significant size differences in the TEM images and the identical intensity ratios between the core-originating peaks (above 800 nm) and both core- and shell- originated peaks (below 600 nm), the core and shell thicknesses are considered to be comparable between the samples in Supplementary Figs. 5 and 8. To further investigate these chemical species, XPS analysis was conducted on samples with different shell thicknesses and on the pinwheel-shaped sample shown in Fig. 1b (Supplementary Fig. 9). In both the thick-shell and pinwheel-shaped samples, the In 3 *d* signal was largely attenuated, whereas the Zn 2*p* spectra were dominated by a ZnSe-like doublet, with the Zn 2*p*₃/₂ component near 1021 eV. Under thin shell conditions, however, both the In 3 *d* and Zn 2*p* spectra exhibited similar trends. First, tracking the interfacial alloy formation was challenging because the reported binding energies of potential alloy species (such as In_2_Se_3_ and Zn_3_As_2_) overlap closely with the conventional InAs and ZnSe peaks^30,31^. Instead, the distinct changes emerged in the higher binding energy regions, suggesting the presence of different chemical species. Specifically, in the pristine sample, additional peaks became apparent near 445.6 and 453.2 eV for In, and 1023.26 and 1046.5 eV for Zn, which are presumed to originate from metal chlorides such as InCl_3_ and ZnCl_2_^32,33^. In contrast, for the oxidized sample, these secondary peaks appeared with increased relative intensity at slightly lower binding energies (near 445.2 and 452.9 eV for In 3 *d* and 1022.9 and 1045.9 eV for Zn 2*p*), which suggests the additional presence of metal oxides such as In_x_O_y_ and Zn_x_O_y_. The prominent spectral contributions from surface ligands and oxidized chemical species in the thin shell samples can be attributed to the increased surface-to-volume ratio, different synthetic conditions, and the limited probing depth of XPS (see Methods and Supplementary Fig. 9 for details). By contrast, the pinwheel-shaped particles consisted of spatially separated InAs cores and ZnSe wing domains, with no clear evidence of additional Zn-related chemical species.

These XPS results establish a difference in the interfacial chemical environments of the pristine and oxidized samples. The early shift of the XRD peaks toward bulk ZnSe in the oxidized thin shells suggests that the oxide layer accommodates the residual lattice mismatch and suppresses strain accumulation as the shell thickens, allowing the ZnSe shell to crystallize closer to its bulk composition from the early stages of growth. Meanwhile, in the pristine core/shell, as the shell thickens and its outer region approaches a pure ZnSe composition, the structural mismatch between the constrained inner interface and the unconstrained outer shell accumulates internal stress. This stress could induce lattice strain that ultimately degrades the optical properties. To investigate the mechanical strain exerted on the InAs core, Raman spectroscopy was performed on pristine and oxidized core/shell CQDs with varying shell thicknesses as shown in Fig. 2f. In the pristine CQDs, the InAs LO phonon band progressively shifts toward higher wavenumbers and broadens as the shell thickness increases from 1–2 to 7 monolayers (Supplementary Fig. 10), consistent with the accumulation of compressive strain in the core^34^. In contrast, the oxidized core/shell CQDs exhibit a LO phonon band that remains largely unchanged in both position and lineshape across all shell thicknesses examined, suggesting that the interfacial oxide region limits the additional accumulation of compressive strain in the core as the ZnSe shell thickens. This thickness-independent behavior in the oxidized samples, contrasting with the pronounced spectral evolution in pristine CQDs, is consistent with the divergence in PLQY observed between the two systems upon thick-shell growth (40% vs. 23%) and with a strain-buffering role of the interfacial oxide during shell thickening.

Furthermore, to observe the initial surface states prior to shell growth, XPS analysis was performed on the pristine and oxidized cores following ZnCl_2_ treatment. For the Zn-treated cores, the Zn 2*p* spectra exhibited a peak at 1023.1 eV for the pristine sample, consistent with Zn remaining largely as residual ZnCl_2_ on the surface (Supplementary Fig. 11). In contrast, the oxidized core exhibited a Zn 2*p* peak at a lower binding energy of 1022.3 eV, consistent with a change from a predominantly ZnCl_2_-like environment toward Zn coordination involving oxygen-containing surface species. A similar shift was observed in the thin-shell samples (1023.3 eV and 1022.9 eV for pristine and oxidized, respectively), indicating that the altered Zn chemical environment detected after ZnCl_2_ treatment is also present during the early stages of shell growth. Nevertheless, both Zn-treated pristine and oxidized cores exhibited a negligible PLQY of less than 1%. To further evaluate the passivation effect after shell growth, time-resolved photoluminescence (TRPL) measurements were performed. Supplementary Fig. 12 shows comparable decay lifetimes between pristine and oxidized CQDs for both thin and thick shells (Supplementary Fig. 12 and Supplementary Table 2). These results suggest that the primary role of the interfacial oxide is to serve as a structural buffer rather than a direct surface passivator. Since interfacial stress and accumulated lattice strain become increasingly severe in larger core/shell systems^27,33,35^, utilizing such an intermediate buffer layer represents a crucial strategy for employing short-wave infrared InAs CQDs in future electroluminescent applications.

### Regulating electron injection in InAs/ZnSe CQD light emitting diodes

**Fig. 3:**
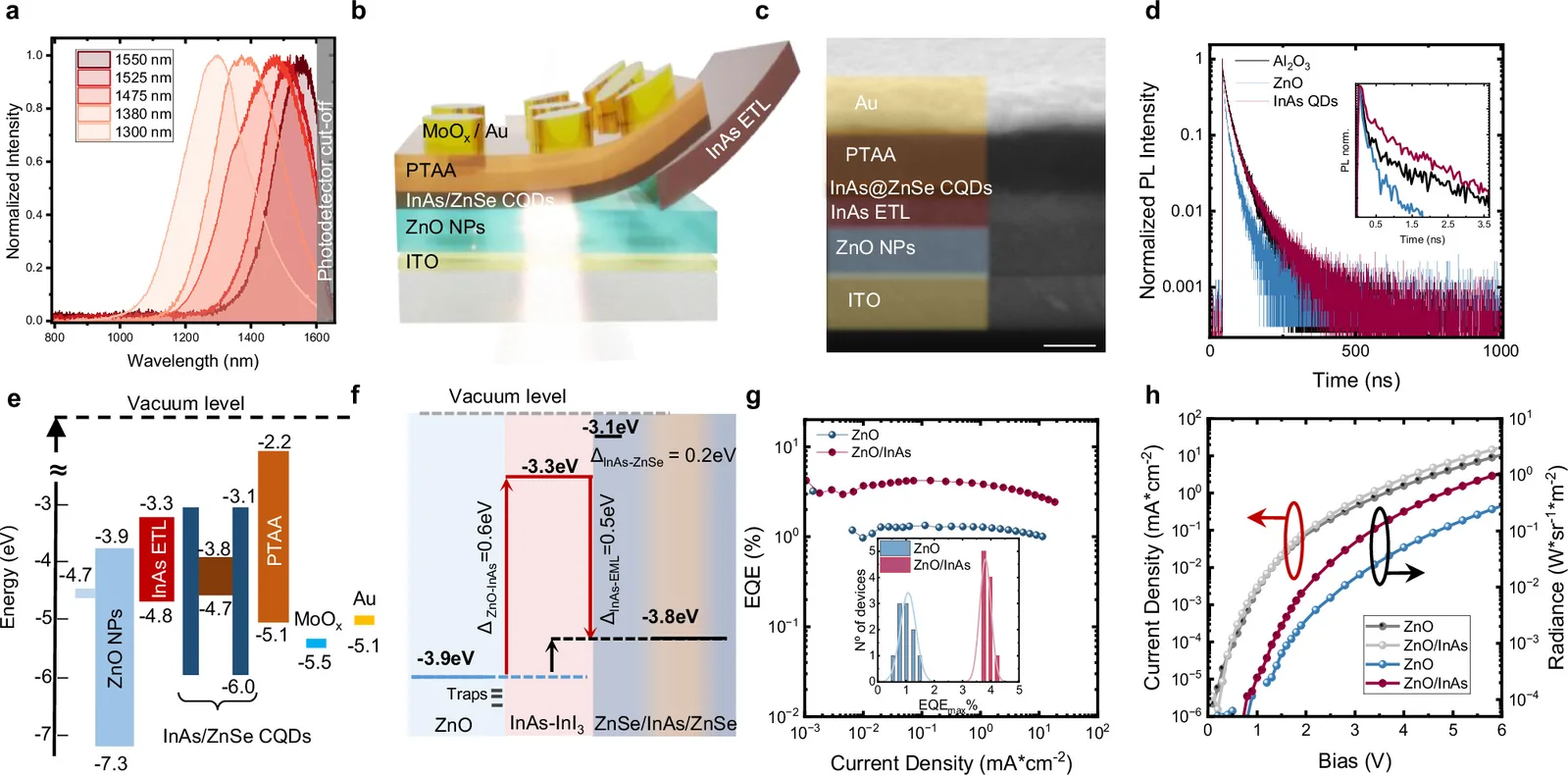
Light-emitting Diodes based on InAs/ZnSe CQDs and ETL optimization. **a** Size-dependent electroluminescence (EL) of InAs/ZnSe quantum dots (QDs), with emission peaks at 1300, 1380, 1450, 1525, and 1550 nm. **b** Device architecture: ITO//ZnO//InAs-ETL//InAs/ZnSe CQDs//PTAA//MoOₓ//Au. **c** Cross-sectional SEM of the device. The white scale bars in Fig. 3c correspond to 100 nm. **d** TRPL of InAs/ZnSe CQDs deposited on Al_2_O_3_ (black), ZnO (blue), and InAs ETL (red). Inset: early-time decay dynamics. **e** Schematic of the energy level alignment and CBM offsets of ZnO, InAs ETL, and InAs/ZnSe. **f** Flat-band energy level alignment of the device structure. **g** External quantum efficiency (EQE) of devices employing ZnO as the sole electron transport layer (blue) and ZnO/InAs ETL (red). **h** Current density-voltage-radiance (J-V-R) characteristics of ZnO-based (blue, black) and ZnO/InAs ETL-based (red, gray) LEDs, showing enhanced radiance at comparable current densities for devices incorporating the InAs ETL.

Leveraging the tunable properties of our InAs/ZnSe CQDs, we successfully demonstrated LEDs across a broad spectral range between 1300 and 1550 nm (Fig. 3a). Device optimization was performed using the largest-core InAs/ZnSe CQDs, which exhibit a first excitonic absorption peak at 1400 nm and a film PL emission centered at 1500 nm, resulting in electroluminescence centered at 1525 nm (Supplementary Fig. 13). These devices employ CQDs coated with a 3-4 ML ZnSe shell (lateral size of ~11 nm) and subjected to controlled surface oxidation for 12 h, yielding a relatively high PLQY of ~40%. Further shell thickening does not appreciably improve PLQY but introduces electron injection barriers that hinder carrier injection. The LEDs adopt an inverted configuration, in which the CQD emissive layer is sandwiched between a transparent indium tin oxide (ITO) cathode serving as the electron-injecting contact and a MoO_x_/Au anode that facilitates efficient hole injection (Fig. 3b). A ZnO-based electron transport layer (ETL) provides efficient electron transport and facilitates hole-blocking^36^. The InAs/ZnSe colloidal CQDs emissive layer is deposited next, followed by a PTAA hole transport layer (HTL). The lower hole mobility of PTAA (~10^−4^−10^−3 ^cm^2 ^V^−1^ s^−1^)^37^, compared to the electron mobility of ZnO ( ~ 10^−3^−10^−2 ^cm^2 ^V^−1^ s^−1^)^36^, leads to electron-dominated transport, causing carrier imbalance. In addition to this injection asymmetry, ZnO-based ETL introduces interfacial non-radiative recombination likely due to surface defects such as oxygen vacancies and hydroxyl groups, which act as trap states and promote exciton quenching at the ZnO/emissive layer interface^38^. Previous strategies employing ultrathin insulating interlayers (e.g., PMMA) have been shown to mitigate quenching and reduce electron injection, but at the cost of hindered charge transport and increased series resistance^19^. To overcome these limitations, we introduce a semiconducting interlayer of wider band-gap InAs CQDs (Eg ~ 1.46 eV), between ZnO and the emissive layer (Fig. 3b, c). The InAs CQDs exhibit a first excitonic absorption peak at ~850 nm, consistent with their small size (~3 nm diameter), and are synthesized via a modified oleic acid (OA)-mediated route (Supplementary Fig. 14)^13^. To enable their dual function, as a charge-regulating interlayer and surface-passivating interface, the OA-capped InAs CQDs undergo solid-state ligand exchange (SSLE) with indium (III) iodide (InI_3_) (Supplementary Fig. 14). This process replaces long OA ligands with shorter inorganic ones, improving charge transport and passivating surface trap states^39^. To directly probe interfacial recombination dynamics, TRPL measurements were performed on InAs/ZnSe CQDs deposited on alumina substrates (Al_2_O_3_, used as reference), and on either ZnO or an InAs CQD layer (Fig. 3d). On an inert Al_2_O_3_ substrate, the CQDs exhibit an average decay lifetime of 32.50 ns, which serves as a reference sample. When deposited on ZnO, the lifetime decreases to 18.69 ns, indicating strong non-radiative recombination induced by ZnO surface defects. In contrast, CQDs deposited on an InAs-InI_3_ layer retain a similar lifetime (30.73 ns; Fig. 3d and Supplementary Table 3), demonstrating that the InAs interlayer effectively suppresses interfacial recombination. This, in turn, is expected to translate into improved radiative efficiency in operating devices^40^. Space-charge-limited current (SCLC) measurements further support improved charge transport in devices incorporating the InAs ETL compared to ZnO-only devices (Supplementary Fig. 15).

Building upon these results, the final device architecture was optimized by incorporating the InAs CQD interlayer as a functional ETL and tuning its thickness relative to ZnO. The optimized structure consists of ITO (100 nm) // ZnO (140 nm) // InAs ETL (40 nm) // InAs/ZnSe (25 nm) // PTAA (120 nm) // MoO_x_ (10 nm) // Au (100 nm) (Fig. 3b), with uniform layer stacking confirmed by cross-sectional SEM (Fig. 3c and Supplementary Fig. 16). The energy level alignment for this architecture is summarized in Fig. 3e. On the hole side, the valence band maximum (VBM) of the InAs emitting core (VBM = −4.7 eV) aligns with PTAA highest occupied molecular orbitals (HOMO = −5.1 eV) and remains close to the UPS-measured value for the InAs/ZnSe system with intermediate shell thickness (VBM = −5.4 eV) (Supplementary Fig. 17). On the electron side, SSLE using halide- based (InI_3_) inorganic ligands (Supplementary Fig. 18) tunes the energetics of the InAs ETL modifying the energy landscape at both the ZnO/InAs ETL and ETL/emissive layer interfaces^41^. The conduction band minimum (CBM) of ZnO, at −3.9 eV to the vacuum level, forms a ~ 0.6 eV barrier with the InAs-InI_3_ ETL (CBM = −3.3 eV), thereby regulating electron injection (Fig. 3f). The InAs ETL is energetically positioned between ZnO and the InAs core of the emitter (CBM = −3.8 eV), while the ZnSe shell (CBM = −2.2 eV) defines the highest-energy barrier. This stepwise alignment lowers the effective injection barrier.

Consistent with the spectroscopic and optoelectronic observations, the incorporation of the ETL InAs-InI_3_ interlayer leads to a clear improvement in device performance. ZnO-only control devices exhibit a peak EQE of 1.6% (EQE_mean_ = 1.1% ± 0.2%, Fig. 3g), a peak radiance of 0.3 W sr^−1^ m^−2^, and a current density of 9 mA cm^−2^ at 6 V (Fig. 3h). In contrast, devices incorporating the InAs-InI_3_ ETL show a threefold increase in EQE, reaching a maximum of 4.2% (EQE_mean_ = 3.8% ± 0.2%, Fig. 3g), together with a reduced turn-on voltage (1.1 V compared to 1.3 V for ZnO-only at R = 2 · 10^−4 ^W sr^−1^ m^−2^). At a fixed bias of 6 V and current density of ~17 mA cm^−2^, the radiance increases to 1.1 W sr^−1^ m^−2^, approximately three times higher than the control devices (Fig. 3h). The enhancement arises from reduced trap density and more efficient charge balance provided by the InAs ETL, consistent with the lower turn-on voltage and higher radiance at the same current density.

### Optimized hole-transport layer for higher radiance

**Fig. 4:**
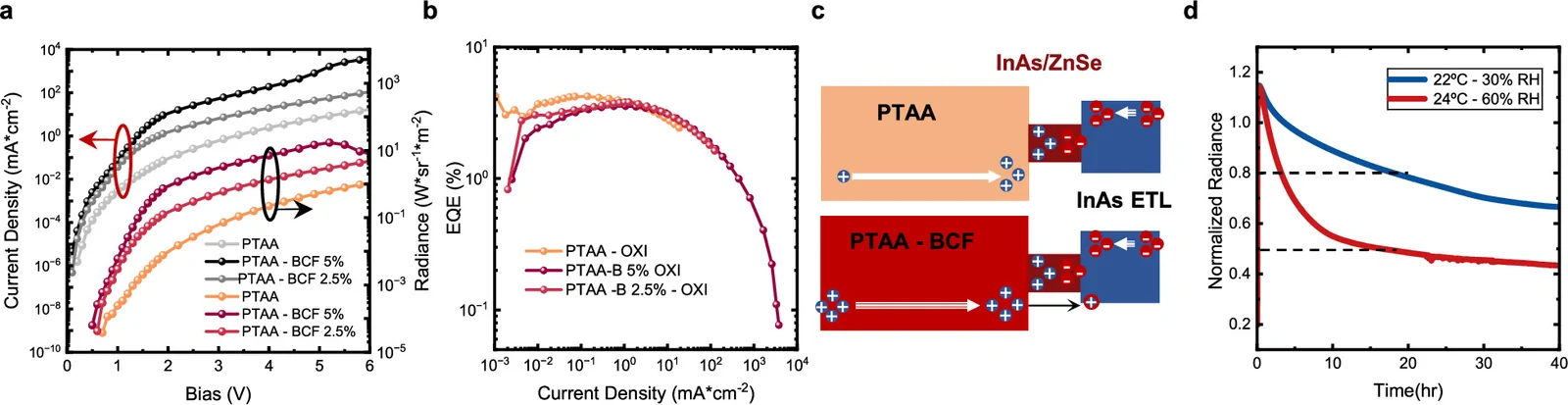
Device characterization upon BCF-doping of PTAA. **a** Current density-voltage-radiance (J-V-R) characteristics of devices with different doping levels. **b** EQE for devices with a general architecture ITO//ZnO//InAs-InI_3_//InAs/ZnSe// HTL //MoOx//Au. EQE of devices employing different concentrations of BCF, 0% (Orange- PTAA), 2.5% (red), and 5% (dark red). **c** Schematic of charge transport. Thick PTAA induces a resistive hole‑transport regime, where low intrinsic conductivity increases the device series resistance and limits hole injection. BCF doping enhances PTAA conductivity, reducing series resistance and boosting hole injection, but leads to hole‑over‑injection due to the limited hole‑blocking ability of the InAs layer, as evidenced by the stronger EQE roll‑off in doped PTAA devices compared to undoped ones. **d** Stability test on ITO//ZnO//InAs ETL//InAs/ZnSe//PTAA-BCF 5% //MoO_x_//Au at a constant current density of 100 mA cm^−2^, under low (blue curve, 22 °C, 30% RH) and high humidity environments (red curve, 24 °C, 60% RH).

We showed that both ZnO-only and InAs-modified devices operate in a hole-induced resistive regime due to ~100 nm thick PTAA, where the device resistance is dominated by limited hole injection and transport through HTL, making hole transport the main bottleneck (Supplementary Fig. 19). As seen in the previous J-V characteristics (Fig. 3h), the current density is limited to ~10-20 mA cm^−2^ at 6 V (Fig. 4a). While this configuration yields EQE_max_ of 4.2%, it also limits radiative output due to reduced current.

To address the charge-limited transport caused by the resistive PTAA layer while maintaining charge balance, molecular doping with tris(pentafluorophenyl)borane (BCF) is employed (Supplementary Fig. 20). As a strong Lewis acid, BCF enhances PTAA conductivity and improves hole mobility^42,43^. An optimal doping level of ~5 wt% ensures efficient charge transport without loss in device performance. This improvement is directly reflected in device performance: upon BCF doping, the maximum current density increases from ~17 mA cm^−2^ to ~3500 mA cm^−2^, while the turn-on voltage decreases from 1.1 V to 0.7 V (Fig. 4a). The maximum radiance correspondingly increases from ~1 W sr^−1^ m^−2^ to ~17 W sr^−1^ m^−2^, while EQE decreases slightly from 4.2% to 3.5% (Fig. 4b). These results are consistent with enhanced hole transport and reduced series resistance, enabling significantly higher radiative output at the expense of a modest reduction in recombination efficiency. Similar behavior is observed for both ZnO and InAs-based devices (Supplementary Fig. 21), although InAs-based devices consistently exhibit higher overall performance.

Finally, the operational stability of the optimized devices was evaluated under constant-current driving in air, without encapsulation. Measurements were performed under a constant current density of 100 mA cm^−2^ (initial bias ~3 V and radiance ~4.4 W sr^−1^ m^−2^) under two ambient conditions (22 °C/30% RH and 24 °C/60% RH). Under low humidity (30% RH), the device reached 80% of its initial radiance (T_80_) after 18 h and remained above ~60% of its initial radiance after 40 h. In contrast, at elevated humidity (60% RH), degradation accelerated markedly: the device reached T_80_ in only 3 h and half of the starting radiance (T_50_) in 16 h, with the radiance decreasing to ~40% of its initial value within the first ~15-20 h before stabilizing. The strong dependence of the operational lifetime on ambient humidity identifies moisture as a key factor governing device degradation. Although BCF doping improves the moisture resistance of the PTAA HTL through stable Lewis acid-polymer interactions^43,44^, the InAs/ZnSe CQD film also exhibits a significant decrease in PL intensity under humid conditions (Supplementary Fig. 22), indicating that multiple layers contribute to degradation. The dominant moisture-sensitive pathway within the complete device stack, however, remains unclear. Under both humidity conditions, the devices exhibit a characteristic transient increase in radiance during the initial stage of operation, followed by gradual degradation. While similar positive-aging behavior has been reported for oxide-based ETLs^45–48^, the additional interfaces introduced by the InAs interlayer make the microscopic origin in the present architecture difficult to identify. Notably, even under the more demanding high-humidity condition, the devices substantially outperform previously reported SWIR InAs CQD LEDs, for which a T_50_ of only 19 min was achieved at a much lower current density (0.2 mA cm^−2^) and initial radiance (~0.3 W sr^−1^ m^−2^), and only with encapsulation^19^. The improved operational stability is attributed to two complementary interfacial strategies: an InAs interlayer that mitigates instability at the ETL/emissive-layer junction, as supported by the TRPL results discussed above, and BCF doping of PTAA, which improves the moisture robustness of the HTL. These results demonstrate that the stability improvements reported here are maintained across a range of ambient conditions rather than representing a best-case scenario, while also highlighting the importance of continued interfacial engineering to further enhance environmental stability.

In summary, high-temperature ZnCl_2_ treatment combined with continuous precursor injection improves the shell nucleation and growth pathway, enabling pristine InAs/ZnSe core/shell CQDs to achieve PLQYs exceeding 40% beyond 1400 nm. Comparative structural and spectroscopic analyses further indicate that controlled interfacial oxidation modifies the core/shell interface and is associated with reduced shell-thickness-dependent strain evolution. Combining optimized shell growth with controlled interfacial oxidation yields measured PLQYs exceeding 55% in the >1400 nm range without relying on toxic or highly reactive precursors. These results identify control of the shell nucleation and growth pathway as the primary determinant of high-efficiency long-wavelength InAs CQD emitters, with interfacial oxidation providing a complementary strategy for limiting additional strain accumulation in thicker-shell structures. Building upon these highly efficient emitters, rational interfacial and transport-layer engineering enables a step-change in SWIR CQD-LED performance. The introduction of the InAs-InI_3_ CQD interlayer effectively suppresses interfacial quenching and improves charge balance, yielding a maximum EQE of 4.2% (3.8 ± 0.2% average) and a threefold radiance enhancement to 1.1 W sr^−1^m^−2^ at 1525 nm. Further optimization via BCF-doped PTAA boosts charge transport, enabling peak radiance up to ~17 W sr^−1^m^−2^ and a reduced turn-on voltage of 0.7 V. The devices also demonstrate a T_80_ operational lifetime up to 18 h under ambient, non-encapsulated conditions. To the best of our knowledge, this simultaneous achievement of long-wavelength emission, high efficiency, high radiance, and operational stability represents state-of-the-art performance for SWIR LEDs based on III-V CQDs (Supplementary Table 4), underscoring the strength and scalability of the proposed architecture.

## Methods

### Chemicals

Indium acetate (In(OAc)_3_, 99.99%), indium(III) iodide (InI_3_, 99.998%), 1-octadecene (ODE, 90%), oleic acid (OA, 90%), 1,1,3,3,5,5-hexamethyltrisiloxane (HMTS, 95%), Zinc acetate dihydrate (Zn(OAc)_2_*2H_2_O), Tris(pentafluorophenyl)borane (BCF, 95%), methanol (MeOH, anhydrous, 99.8%) and toluene (anhydrous, 99.8%), Potassium hydroxide (KOH, 99.99%), Chloroform anhydrous (99.8%), Ethanol (EtOH, anhydrous) were purchased from Sigma-Aldrich. Butanol (BuOH, 99.9%) was purchased from Thermo Scientific. Indium(I) chloride (In(I)Cl, 99.995%) was purchased from Apollo Scientific. Poly-[bis(4-phenyl)(2,4,6-trimethylphenyl)]amine (PTAA) was purchased from Ossila. Tris(dimethylamino)arsine (amino-As) was purchased from DOCKWEILER CHEMICALS. Tri-n-octylphosphine (TOP, 97%) was purchased from STREM. Oleylamine (OLAm, 95%) was purchased from BIOSYNTH.

### InAs-OLAm 1200−1400 nm core synthesis and oxidation

Using a modified version of a previously reported method^12^, 6 mL of OLAm was degassed at 100 °C for over 2 h. Subsequently, 0.3 mL of TOP, 225 mg of In(I)Cl, and 90 μL of amino-As were mixed at room temperature to form precursor clusters. To achieve the target wavelengths, the reaction was then carried out at 280, 290, or 300 °C for 1 h, yielding first excitonic absorption peaks at 1200, 1300, and 1400 nm, respectively. Finally, the purification process was conducted inside a glovebox using toluene and BuOH. First, an excess amount of BuOH was added to precipitate the crude CQDs. The resulting precipitate was redispersed in toluene, and a 1:1 toluene:BuOH volume ratio was used to selectively precipitate indium oxide and other reaction by-products. In the final purification step, the CQDs were precipitated using a toluene:BuOH volume ratio ranging from 1:2 to 1:4, depending on the CQD size. Each precipitation step was followed by centrifugation at approximately 2800 × *g*. The oxidation process was carried out at 20−23 °C and a relative humidity of 30% ± 5% for 12 h. A blue shift of approximately 25–35 meV was used as an additional criterion for confirming the targeted oxidation condition.

### InAs/ZnSe-OLAm 1200-1400 nm core/shell synthesis

The synthesis was performed following a modified literature procedure^26^. Depending on the desired shell thickness, 5, 7.5, or 10 mL of OLAm (corresponding to approximately 7, 3-4, and 1-2 monolayers, respectively) was mixed with 50 mg of purified InAs cores and 463.4 mg of ZnCl_2_. This mixture was degassed at 110 °C for over 2 h. Separately, a 1 M TOP-Se precursor solution was prepared by stirring 268.5 mg of Se powder in 3.4 mL of TOP at 150 °C for at least 10 min. Following degassing, 1.7 mL of the TOP-Se solution was injected into the core mixture at 90 °C, and the temperature was subsequently raised to 300 °C. Upon reaching 300 °C, the remaining TOP-Se solution was continuously injected at a rate of 1.7 mL/h over a period of 1 h. Notably, 30 min after the start of this continuous injection, the reaction temperature was further increased to 320 °C for the remainder of the growth process. The crude reaction mixture was precipitated by adding BuOH at a crude:BuOH volume ratio of 1:1 to 1:2, followed by centrifugation at approximately 1,250 × *g*. The resulting precipitate was redispersed in toluene and centrifuged again at approximately 1,250 × *g*. The supernatant was then collected and filtered.

### InAs-OA 850 nm core synthesis

The 850 nm InAs CQDs were synthesized using an OA-mediated method adapted from reported paper^49^. 440 mg of In(OAc)_3_ was loaded into a 50 mL three-neck round-bottom flask along with 1.5 mL of OA and 6 mL of ODE. The mixture was briefly degassed under vacuum at room temperature, then heated to 110 °C and degassed for 1 h under vacuum with magnetic stirring using a Schlenk line. After degassing, the atmosphere was switched to argon, and 0.3 mL of degassed TOP was injected. The solution was then heated to 245 °C. A mixed arsenic precursor solution was prepared by blending 90 µL of amino-As with 1.2 mL of HMTS. The injection was performed in two steps: first, 100 µL of the precursor mixture was injected at 0.66 mL/min to form the InAs seeds. For CQD growth, 1.2 mL of the arsenic precursor solution was dropwise added to the reaction mixture containing the seeds at a rate of 1.2 mL/h. After completion of the injection, the reaction was quenched by cooling to room temperature, and the solution was transferred into a glovebox under inert atmosphere for further handling. 4 mL of anhydrous toluene was added to the crude reaction mixture to redisperse and stabilize the CQDs. CQDs were then precipitated by adding BuOH in a 1:4 volume ratio (CQDs:BuOH) and centrifuging for 5 min at approximately 2800 × *g*. The precipitate was redispersed in 10 mL of toluene, followed by the addition of a 1:0.5 toluene:BuOH mixture, and centrifuged again at approximately 2800 × *g* for 5 min to remove most oxide byproducts. The resulting supernatant was collected, and 20 mL of BuOH was added to fully precipitate the CQDs. The pellet was redispersed in 5 mL of toluene and further precipitated by adding EtOH in a 1:1 ratio. Finally, the purified CQDs were redispersed in octane at a concentration of 40 mg/mL and stored under an inert atmosphere until device fabrication.

### ZnO nanocrystal synthesis

ZnO nanocrystals were synthesized following a previously reported procedure^50^. Briefly, 2.95 g of Zn(OAc)_2_*2H_2_O was dissolved in 125 mL of MeOH under magnetic stirring at 60 °C in a 250 mL round-bottom flask. In parallel, 1.49 g of KOH was dissolved in 65 mL of MeOH. The KOH solution was added dropwise to the zinc acetate solution at a rate of 12 mL/min under magnetic stirring. The reaction mixture was maintained at 60 °C for 2.5 h. After cooling to room temperature, the ZnO nanocrystals were precipitated by centrifugation at approximately 2800 × *g* for 5 min and redispersed in fresh MeOH. This washing step was repeated twice (using 1:1 and 1:0.5 solvent-to-initial-volume ratios) to remove residual precursors and byproducts. The final white pellet was redispersed in chloroform containing 1 vol% butylamine to ensure colloidal stabilization.

### Device fabrication

ITO-coated glass substrates were sequentially cleaned by ultrasonication for 30 min in the following solvents: detergent solution, deionized water, acetone, and isopropanol. The substrates were then treated under UV–ozone for 30 min to improve surface cleanliness and wettability. A ZnO nanocrystal layer (60 mg/mL in chloroform) was spin-coated at 3000 rpm for 35 s and immediately transferred into a nitrogen-filled glovebox without further annealing. Subsequently, an InAs-OA CQD layer (40 mg/mL in octane) was deposited by spin-coating at 2000 rpm for 15 s. For solid-state ligand exchange, a 5 mg/mL solution of InI_3_ in MeOH was drop-cast onto the film and allowed to react for 30 s. The substrate was then spin-coated at 2000 rpm for 45 s, during which the film was rinsed twice with MeOH and once with toluene to remove excess ligands and reaction by-products. The emissive layer, consisting of InAs/ZnSe CQDs (30 mg/mL in octane), was spin-coated at 2000 rpm, followed by a mild annealing step at 60 °C for 2 min to minimize erosion effects during subsequent processing. A PTAA solution (24 mg/mL in toluene, doped with an appropriate amount of BCF (2.5 or 5 wt%)) was then spin-coated onto the CQD layer and annealed at 90 °C for 20 min. Finally, the devices were transferred to a thermal evaporator, with air exposure kept below 15 min. MoO_x_ (10 nm) and Au (100 nm) were sequentially deposited through a shadow mask that limited the pixel area to a circle of 3.14 mm^2^, at deposition rates of 0.1 Å/s and 1 Å/s, respectively, under a base pressure of ~7 × 10^−6 ^mbar.

### LED characterization

All devices were fabricated and characterized under ambient conditions. Current density–voltage–radiance (J-V-R) characteristics were measured using a computer-controlled source-measure unit (Keithley 2400). Radiance measurements were independently verified using a NIST-traceable Ge photodetector (Newport 818-IR) equipped with a calibrated DB15 module, ensuring consistency across the detection range. Electroluminescence spectra were collected through an optical fiber coupled to a spectrometer (Andor system, see Material Characterization), enabling spectral measurements in the range 800-1600 nm. The external quantum efficiency (EQE) and radiance were determined following the method described in reported paper^1,51^. Assuming Lambertian emission, the total optical flux emitted into the forward hemisphere is given by

1 $${F}_{{ext}}=\pi {{\cdot }L}_{0}$$

where *L*_*0*_ is the radiance normal to the emitting surface. The radiance was determined from the optical power measured by the photodetector according to

2 $${L}_{0}=\,\frac{{P}_{\det }{{\cdot }d}^{2}\,}{{S}_{\det }}$$

where *P*_*det*_ is the detected optical power measured through a 3.8mm^2^ pinhole, *S*_*det*_ is the active area of the photodetector, and *d* is the distance between the device and the detector. The solid angle was determined by accounting for the device geometry and the thickness of the glass substrate. The EQE was then calculated as

3 $${EQE}=\frac{{\pi {{\rm{\cdot }}}P}_{\det }{{\cdot }}{d}^{2}{{\cdot }}\lambda {{\cdot }}e\,}{{S}_{\det }{{\cdot }}h{{\cdot }}c{{\cdot }}I}$$

where *λ* is the EL peak wavelength, *I* is the device current, *e* is the elementary charge, *h* is Planck’s constant, and *c* is the speed of light. The radiance was calculated as

4 $$R=\frac{{P}_{\det }{{\cdot }}{d}^{2}\,}{{S}_{\det }{{\cdot }}{S}_{{LED}}}$$

where *S*_*LED*_ is the emitting pixel area. Stability tests were performed without further encapsulation. Stability measurements were carried out under a controlled atmosphere. All devices were driven under a fixed current density of 100 mA cm^−2^. The power intensity was recorded as a function of time and is reported as normalized intensity.

### Material characterization

Material characterization was carried out using complementary optical, spectroscopic, and structural techniques. UV-vis-NIR absorption spectra in solution were recorded using a Cary 5000 spectrophotometer. Steady-state photoluminescence (PL) measurements were performed using a multi-wavelength laser excitation source (Thorlabs) and detected with a Kymera 328i spectrograph, covering emission up to 1650 nm; absolute photoluminescence quantum yield (PLQY) was determined under 935 nm excitation using an integrating sphere coupled to an Andor detector system. Absolute PLQY was measured using an integrating sphere following the method of de Mello et al^52^. To correct the wavelength-dependent attenuation introduced by the PLQY optical path, the same broadband lamp was measured through (i) a reference fiber path that did not produce the 1400 nm attenuation feature (I_ref_) and (ii) the complete integrating-sphere/fiber configuration used for the PLQY measurements (I_full_), under otherwise identical acquisition conditions. The correction factor was calculated as I_ref_(λ)/I_full_(λ) and applied to the solution-phase PL spectra. For samples whose emission extended beyond the nominal detector range, the corrected spectrum within the directly measured range was fitted with a single Gaussian function, and only the unmeasured spectral tail was obtained from the fit. Extended-range detector measurements confirmed that no substantial additional emission component was present beyond the fitted range. Measurement repeatability was further confirmed by four consecutive measurements of the same sample at 3 h intervals, with a relative PLQY variation below 1%. Extended-range measurements using a detector covering wavelengths up to 2.2 μm confirmed the absence of substantial additional emission at longer wavelengths. In addition, the wavelength-dependent attenuation introduced by the integrating-sphere/fiber optical path was experimentally determined using broadband-lamp measurements and corrected as described in the Methods. These validation results are summarized in Supplementary Fig. 23. The reported absolute PLQY values carry an estimated relative instrumental uncertainty of approximately 7%, based on the detector quantum-efficiency and grating-efficiency calibrations. Furthermore, we observed that OLAm exhibits slight reabsorption in the infrared region, which decreases the measured PLQY by approximately 3%; however, no sample-specific correction was applied because the residual OLAm concentration could not be quantified accurately for each sample. Time-resolved PL (TRPL) measurements were conducted using a pulsed laser diode (827 nm, LDH-D-C-830), with emitted photons detected by a superconducting nanowire single-photon detector (ID281); photon arrival times were recorded via time-correlated single-photon counting (TCSPC) using an ID1000, and decay traces were reconstructed into intensity–time histograms and analyzed using Origin 2025. X-ray photoelectron spectroscopy (XPS) and ultraviolet photoelectron spectroscopy (UPS) spectra were referenced to the C-C component of the adventitious C 1 *s* peak at 284.8 eV and analyzed using CasaXPS, with a Shirley background subtraction applied over the binding energy range of interest. The corresponding full-survey XPS spectra are shown in Supplementary Fig. 24. UPS measurements were performed in the same chamber using a He I discharge lamp (hν = 21.22 eV), with a pass energy of 10 eV and a dwell time of 100 ms; the work function (φ) was determined from the secondary electron cutoff (E_SE_), and the ionization energy (E_ion_) from the spectral width using the valence band maximum (E_VBM_), with E_SE_ and E_VBM_ extracted by linear fitting of the high- and low-binding-energy edges (±0.1 eV). Raman spectroscopy of thin films was performed using a Renishaw inVia confocal Raman microscope equipped with a 100× objective, maintaining laser power below 1 mW to minimize sample heating and degradation at room temperature. X-ray diffraction (XRD) data were collected on drop-cast powder samples using a Rigaku SmartLab diffractometer. Transmission electron microscopy (TEM), including scanning TEM (STEM) and electron energy loss spectroscopy (EELS), was carried out at the Scientific and Technological Centres of the University of Barcelona using a JEOL 2100 F and a probe-corrected JEOL JEM ARM200F NEOARM operated at 200 kV, with EELS acquired using a Gatan GIF Continuum equipped with a K3 detector; samples were prepared by drop-casting CQD solutions onto ultrathin carbon grids.

## Supplementary information

Supplementary Information

Transparent Peer Review file

## Source data

Source Data

## Data Availability

All data supporting the findings of this study are available within the Article, its Supplementary Information and the accompanying Source Data files. Source data are provided with this paper.
