# Supplementary material for: Controlling the shell growth mechanism and interfacial oxidation of InAs/ZnSe quantum dots for shortwave infrared emission
Source: Nat Commun. 2026 Oct 19;17:9969. doi: 10.1038/s41467-026-77896-0 (PMC13680504; doi:10.1038/s41467-026-77896-0)
Supplement: Supplementary file 1 [file 41467_2026_77896_MOESM1_ESM.pdf]

Supplementary Information

Controlling the Shell Growth Mechanism and Interfacial Oxidation of InAs/ZnSe Quantum Dots for Shortwave Infrared Emission

Taewan Kim[1,§], Manuela De Franco[1,§], Miguel Dosil[1], Jae Taek Oh[1], Rajesh Bera[1], Marta Martos Valverde[1], Gaurav Kumar[1] & Gerasimos Konstantatos[1,2 *]

Affiliations

1- ICFO–Institut de Ciències Fotòniques, The Barcelona Institute of Science and Technology, Castelldefels, Barcelona, 08860, Spain

2- ICREA–Institució Catalana de Recerca i Estudis Avançats, Lluis Companys 23, Barcelona, 08010, Spain

§These authors contributed equally: Taewan Kim and Manuela De Franco.

*****e-mail: gerasimos.konstantatos@icfo.eu

Supplementary Information – Contents

Supplementary Fig. 1 Structural and elemental analysis of InAs/ZnSe core/shell CQDs at the initial stage of shell growth

Supplementary Fig. 2 Structural and elemental analysis of InAs/ZnSe core/shell CQDs synthesized via the continuous injection method

Supplementary Fig. 3 Epitaxial registry of the ZnSe shell and schematic of the inverse-oriented core/shell interface

Supplementary Fig. 4 Evolution of core optical and surface chemical properties during controlled oxidation

Supplementary Fig. 5 Optical and structural comparison of pristine and oxidized InAs/ZnSe thin-shell (1–2 ML) core/shell CQDs

Supplementary Fig. 6 Elemental distribution and line-profile analysis of pristine InAs/ZnSe core/shell CQDs

Supplementary Fig. 7 Elemental distribution and line-profile analysis of oxidized InAs/ZnSe core/shell CQDs

Supplementary Fig. 8 Optical and structural comparison of pristine and oxidized InAs/ZnSe thick-shell (7 ML) core/shell CQDs

Supplementary Fig. 9 XPS spectra of In 3*d* and Zn 2*p* regions for pinwheel, pristine, and oxidized InAs/ZnSe core/shell CQDs

Supplementary Fig. 10 Two-component Lorentzian fitting of the InAs LO phonon mode for pristine and oxidized InAs/ZnSe core/shell CQDs

Supplementary Table 1 Lorentzian fitting parameters of the InAs LO phonon mode for pristine and oxidized InAs/ZnSe core/shell CQDs

Supplementary Fig. 11 XPS spectra of the Zn 2*p* region for Zn-treated pristine and oxidized InAs cores prior to shell growth

Supplementary Fig. 12 TRPL of pristine and oxidized InAs/ZnSe core/shell CQDs with thin and thick shells

Supplementary Table 2 TRPL decay parameters of pristine and oxidized InAs/ZnSe thin- and thick-shell nanocrystals

Supplementary Fig. 13 Optical and electroluminescence properties of InAs/ZnSe quantum dots and device operation

Supplementary Fig. 14 Optical properties of InAs-OA quantum dots and schematic of solid-state ligand exchange

Supplementary Table 3 TRPL decay parameters of InAs/ZnSe nanocrystals on various ETLs

Supplementary Fig. 15 SCLC characteristics of ZnO and InAs electron-transport layers

Supplementary Fig. 16 Cross-sectional elemental mapping of the device structure

Supplementary Fig. 17 Evolution of the electronic structure with ZnSe shell thickness

Supplementary Fig. 18 UPS characterization and energy-level alignment of InAs quantum dots before and after InI_3 treatment

Supplementary Fig. 19 Effect of PTAA thickness on charge injection and device performance

Supplementary Fig. 20 PTAA and PTAA-BCF characterizations

Supplementary Fig. 21 ZnO and ZnO/InAs ETLs with PTAA-BCF

Supplementary Fig. 22 Accelerated moisture-aging test of PTAA and PTAA-BCF

Supplementary Table 4 Comparison of maximum EQE in SWIR light-emitting diodes based on CQDs

Supplementary Fig. 23 Reproducibility and validation of the PLQY measurements

Supplementary Fig. 24 Full-survey XPS spectra of Zn-treated InAs cores and InAs/ZnSe core/shell CQDs

Supplementary References

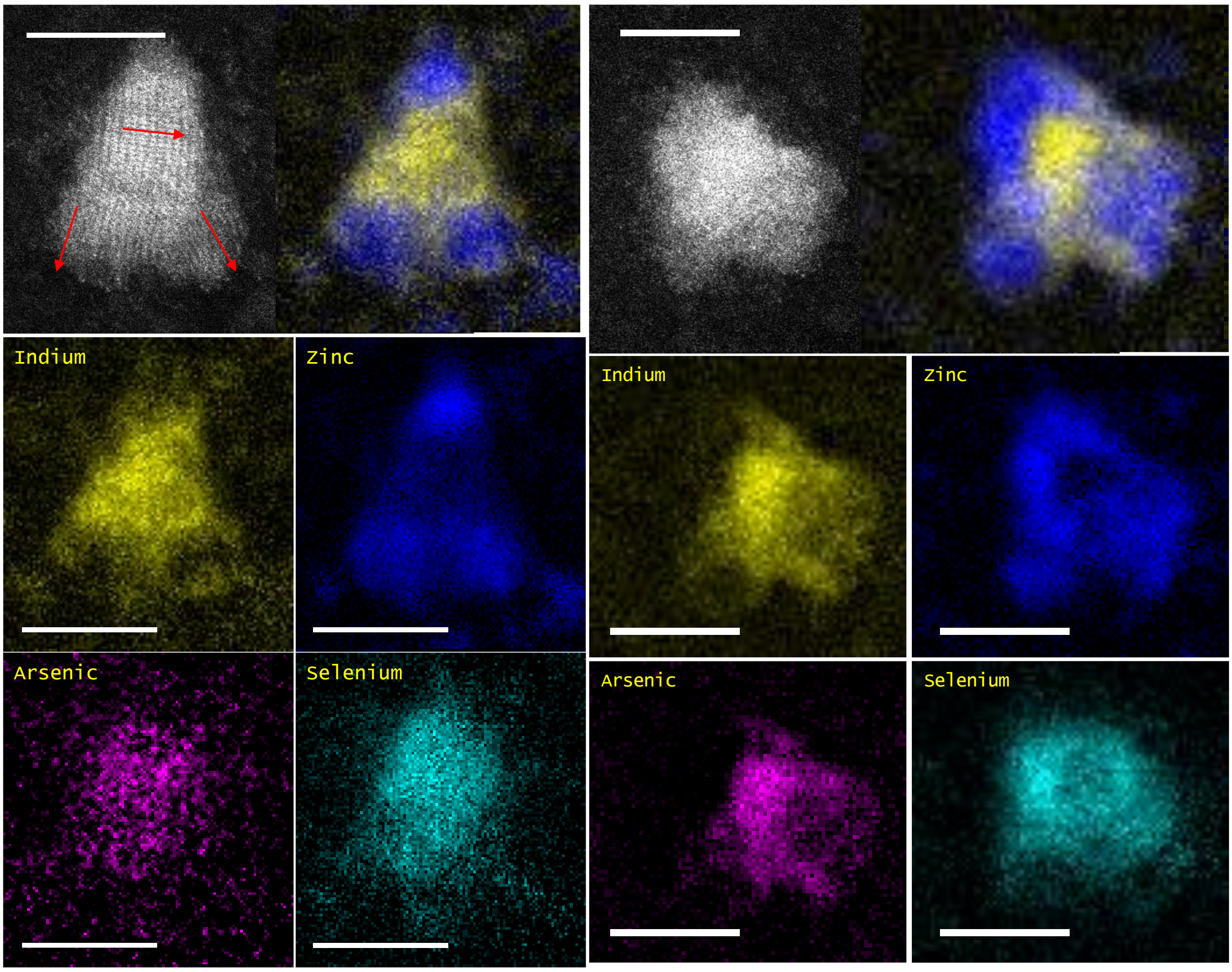

Supplementary Fig. 1. Structural and elemental analysis of InAs/ZnSe core/shell CQDs at the initial stage of shell growth. High-resolution HAADF-STEM images and corresponding EELS elemental maps of two representative particles. Zn-rich domains are preferentially localized near the vertices of the InAs cores, whereas Se is distributed more broadly over the particles. In the high-resolution image, lattice fringes within the vertex-localized Zn-rich domain exhibit an orientation distinct from that of the adjacent InAs core, as indicated by the guide lines. This local lattice misorientation demonstrates that the initial ZnSe-rich domains do not form through a conformal continuation of the InAs core lattice and supports a vertex-initiated island-nucleation pathway. All white scale bars in the TEM images correspond to 5 nm.

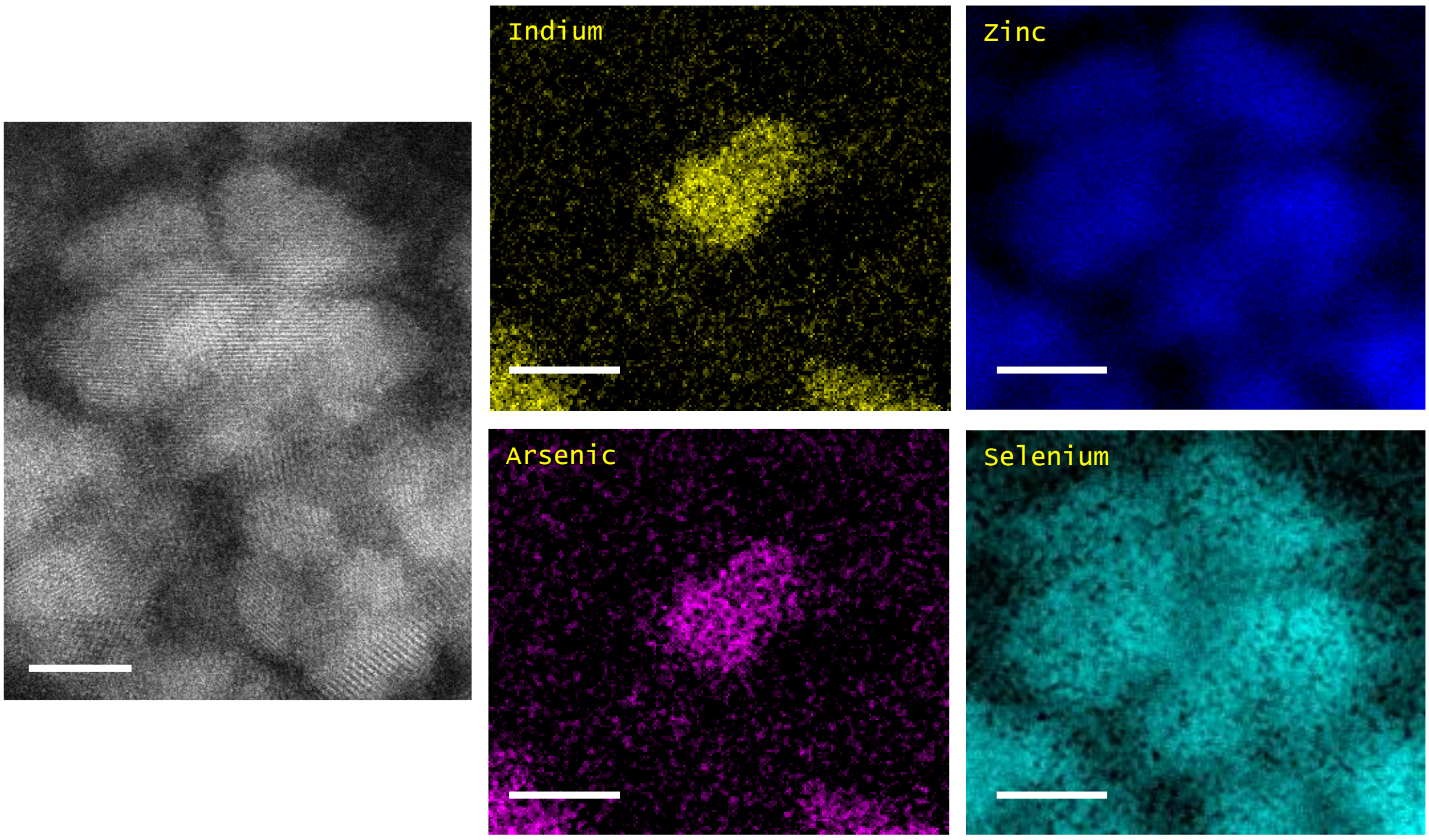

Supplementary Fig. 2. Structural and elemental analysis of InAs/ZnSe core/shell CQDs synthesized via the continuous injection method. Left HAADF-STEM image and **right** corresponding EELS elemental maps of indium, zinc, arsenic, and selenium for core/shell CQDs synthesized using the continuous injection protocol (Fig. 1b). All white scale bars in the TEM images correspond to 5 nm.

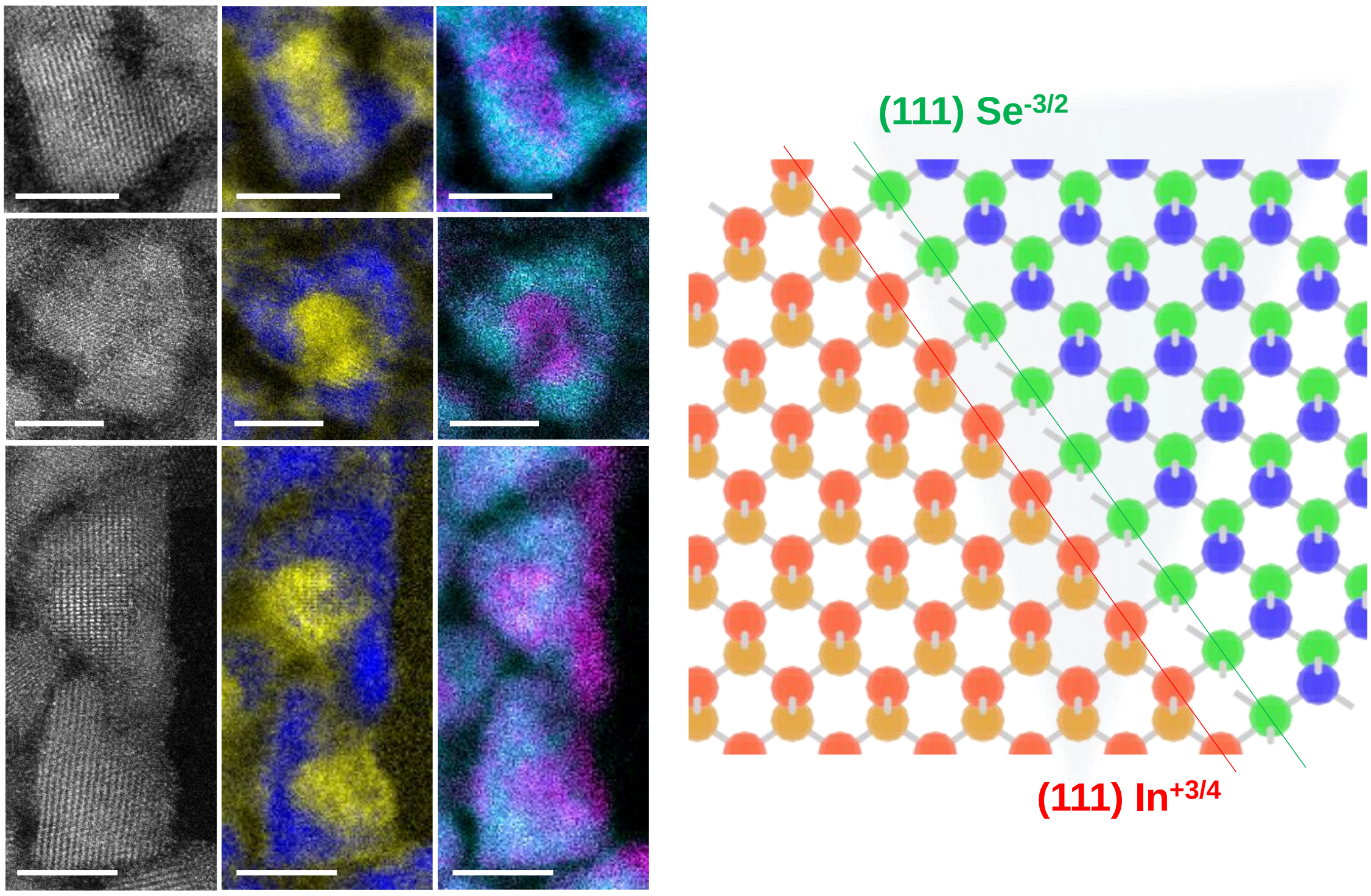

Supplementary Fig. 3. Epitaxial registry of the ZnSe shell and schematic of the inverse-oriented core/shell interface. Left HAADF-STEM images and corresponding EELS elemental maps (In, yellow; Zn, blue; As, magenta; Se, cyan) of three different InAs/ZnSe core/shell CQDs, showing continuous lattice fringes across the core/shell interface. **Right** schematic illustration of the inverse-oriented interface between the InAs (111) and ZnSe (111) facets, highlighting the charge disparity between $In^{3+/4}$ and $Se^{-3/2}$ planes. All white scale bars in the TEM images correspond to 5 nm.

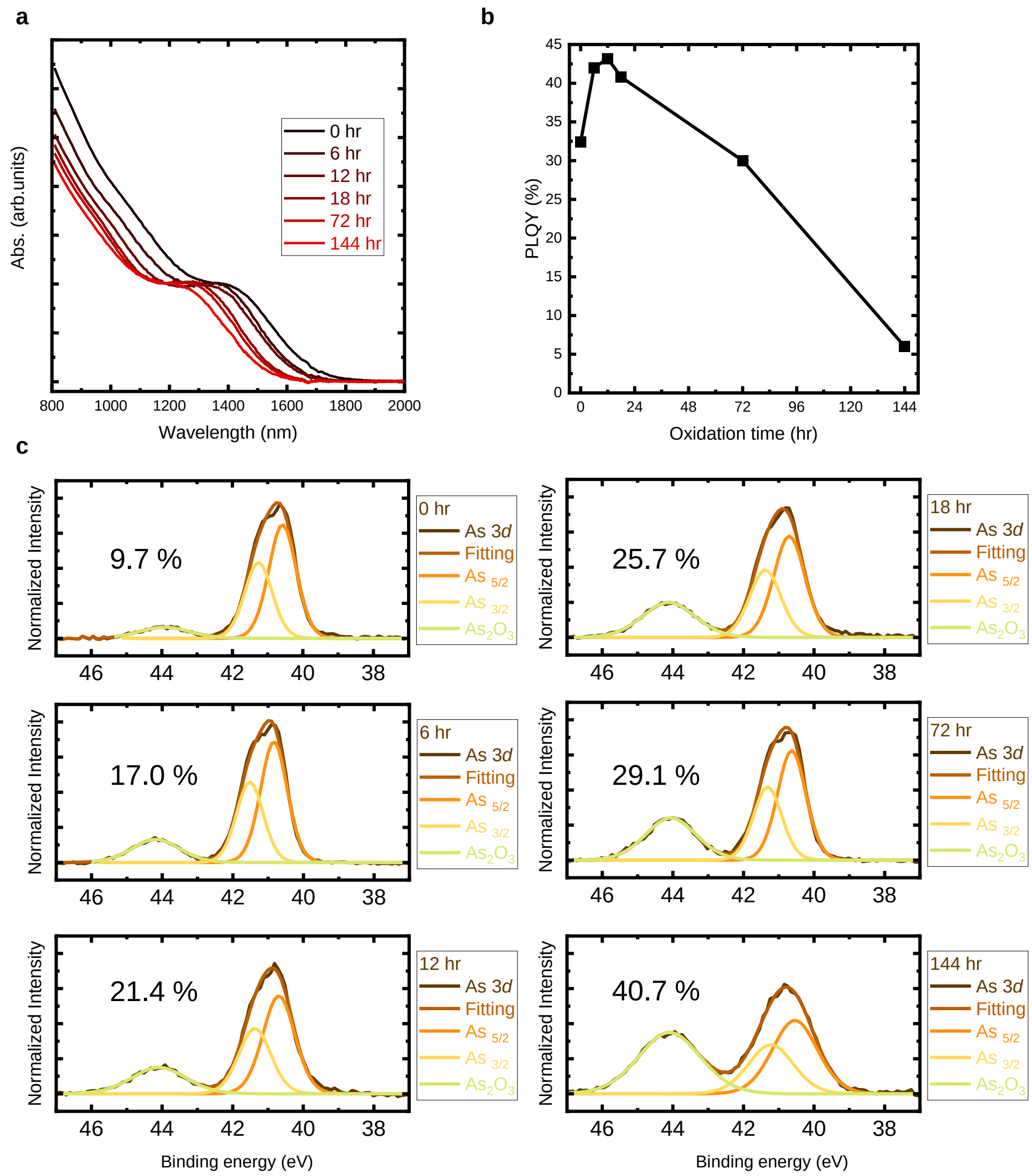

Supplementary Fig. 4 | Evolution of core optical and surface chemical properties during controlled oxidation. a Absorption spectra of InAs cores after air oxidation for 0, 6, 12, 18, 72, and 144 h, showing a progressive blue shift of the first excitonic transition with increasing oxidation time. **b** PLQY of the corresponding InAs/ZnSe core/shell CQDs as a function of the core oxidation time, showing that the final PLQY depends on the oxidation extent of the starting core. **c** High-resolution XPS spectra of the As 3*d* and oxide-related As fraction for InAs cores oxidized for different times, together with peak fitting analysis. The fitted oxide-related As component, assigned to As_2O_3, was used to evaluate the extent of surface oxidation as a function of oxidation time. The As_2O_3 fraction, calculated as the ratio of the peak area near 44 eV to the total As peak area, increased from 9.7% to 40.7% as the oxidation time increased.

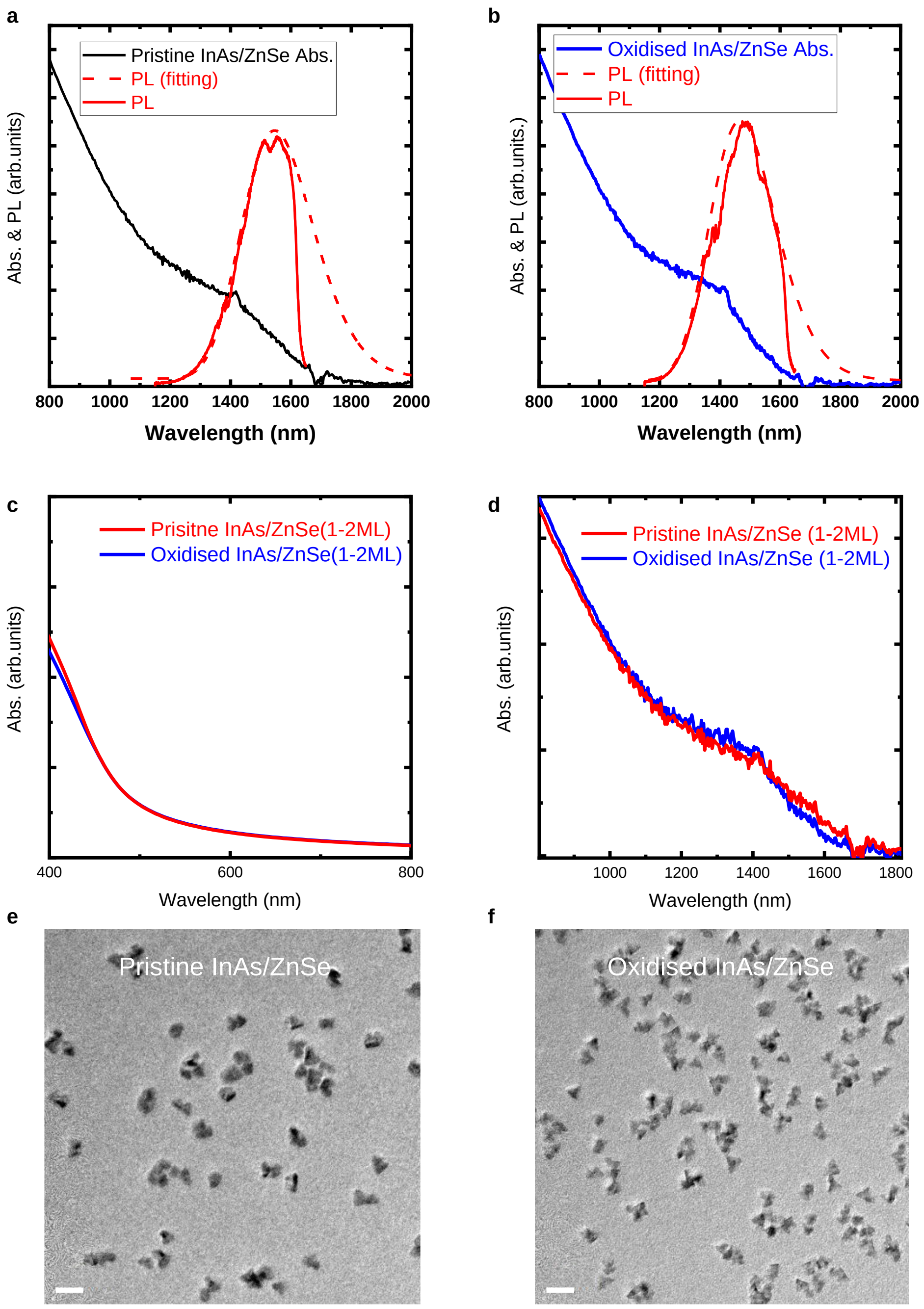

Supplementary Fig. 5. Optical and structural comparison of pristine and oxidized InAs/ZnSe thin-shell (1–2 ML) core/shell CQDs. a, b Absorbance and PL spectra (raw and fitted) of pristine (a) and oxidized (b) thin-shell InAs/ZnSe CQDs. **c, d** Overlaid absorbance spectra of the pristine and oxidized samples in the 400–800 nm (c) and 800–1800 nm (d) ranges, showing comparable intensity ratios between the core- and shell-originated absorption features. **e, f** TEM images of the pristine (e) and oxidized (f) thin-shell CQDs, showing comparable particle size and morphology. All white scale bars in the TEM images correspond to 20 nm.

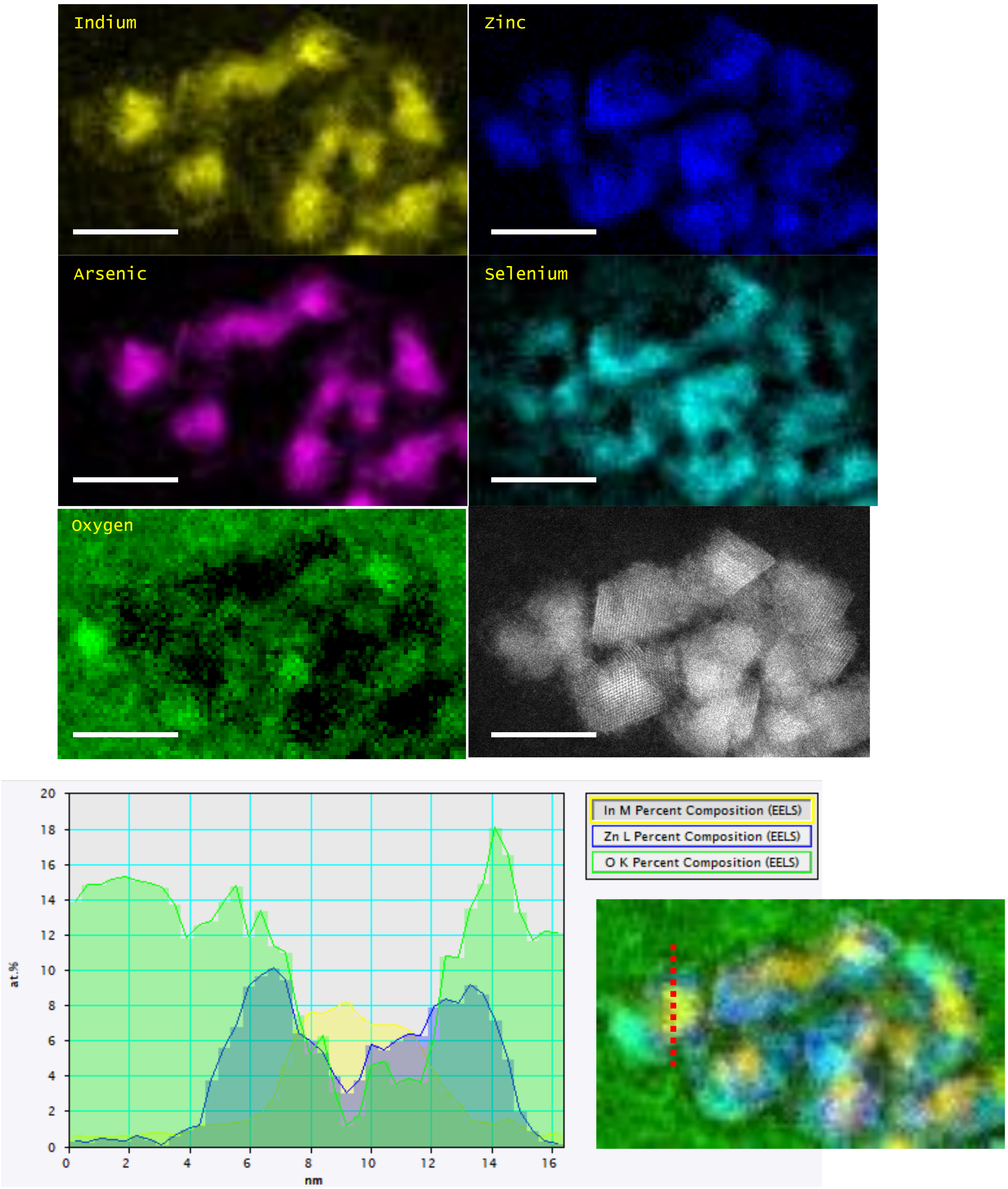

Supplementary Fig. 6. Elemental distribution and line-profile analysis of pristine InAs/ZnSe core/shell CQDs. HAADF-STEM image and corresponding EELS elemental maps of indium, zinc, arsenic, selenium, and oxygen for a cluster of pristine InAs/ZnSe core/shell CQDs. Bottom left, line profile of In, Zn, and O atomic percentage along the dashed line indicated in the overlay image (bottom right). Although a background oxygen signal is present throughout the sample due to methanol treatment during TEM preparation, the oxygen profile does not show localized co-enhancement with indium, in contrast to the oxidized sample (Supplementary Fig. 7). All white scale bars in the TEM images correspond to 10 nm.

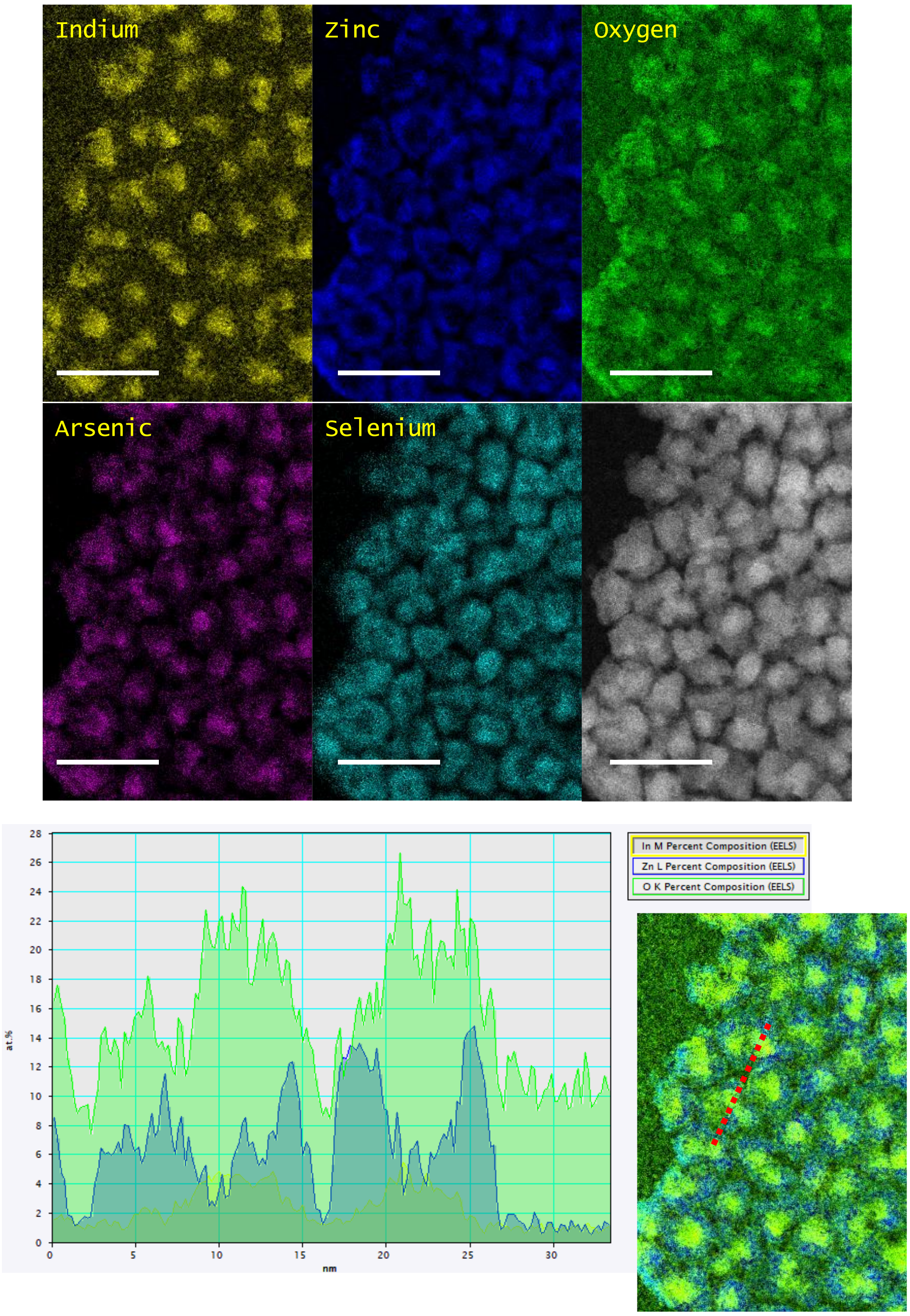

Supplementary Fig. 7. Elemental distribution and line-profile analysis of oxidized InAs/ZnSe core/shell CQDs. HAADF-STEM image and corresponding EELS elemental maps of indium, zinc, arsenic, selenium, and oxygen for a cluster of oxidized InAs/ZnSe core/shell CQDs. Bottom left, line profile of In, Zn, and O atomic percentage along the dashed line indicated in the overlay image (bottom right), showing that the oxygen signal is enhanced at positions coinciding with indium, in contrast to the pristine sample (Supplementary Fig. 6). All white scale bars in the TEM images correspond to 10 nm.

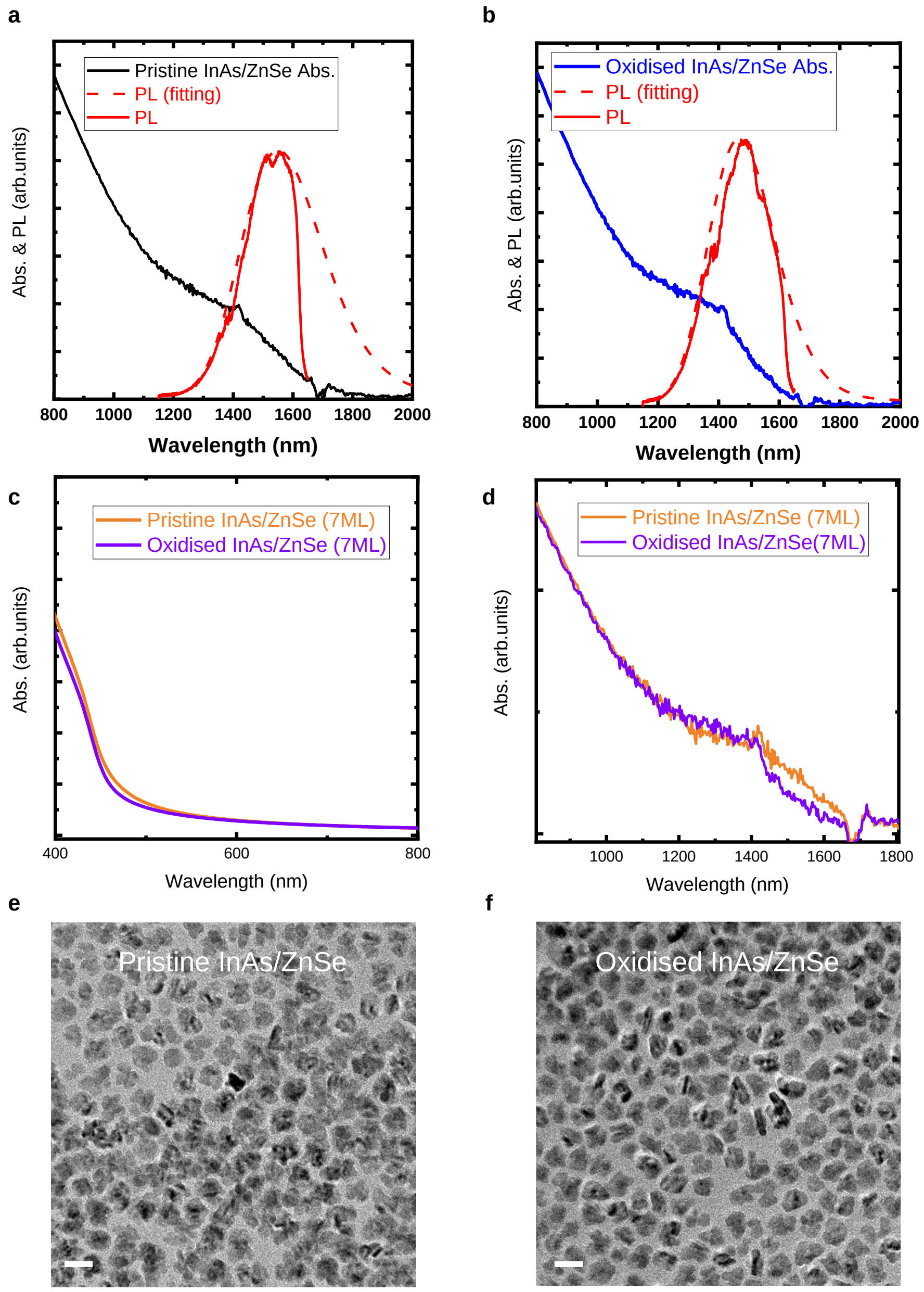

Supplementary Fig. 8. Optical and structural comparison of pristine and oxidized InAs/ZnSe thick-shell (7 ML) core/shell CQDs. a, b Absorbance and PL spectra (raw and fitted) of pristine (a) and oxidized (b) thick-shell InAs/ZnSe CQDs. **c, d** Overlaid absorbance spectra of the pristine and oxidized samples in the 400–800 nm (c) and 800–1800 nm (d) ranges, showing comparable intensity ratios between the core- and shell-originated absorption features. **e, f** TEM images of the pristine (e) and oxidized (f) thick-shell CQDs, showing comparable particle size and morphology. All white scale bars in the TEM images correspond to 20 nm.

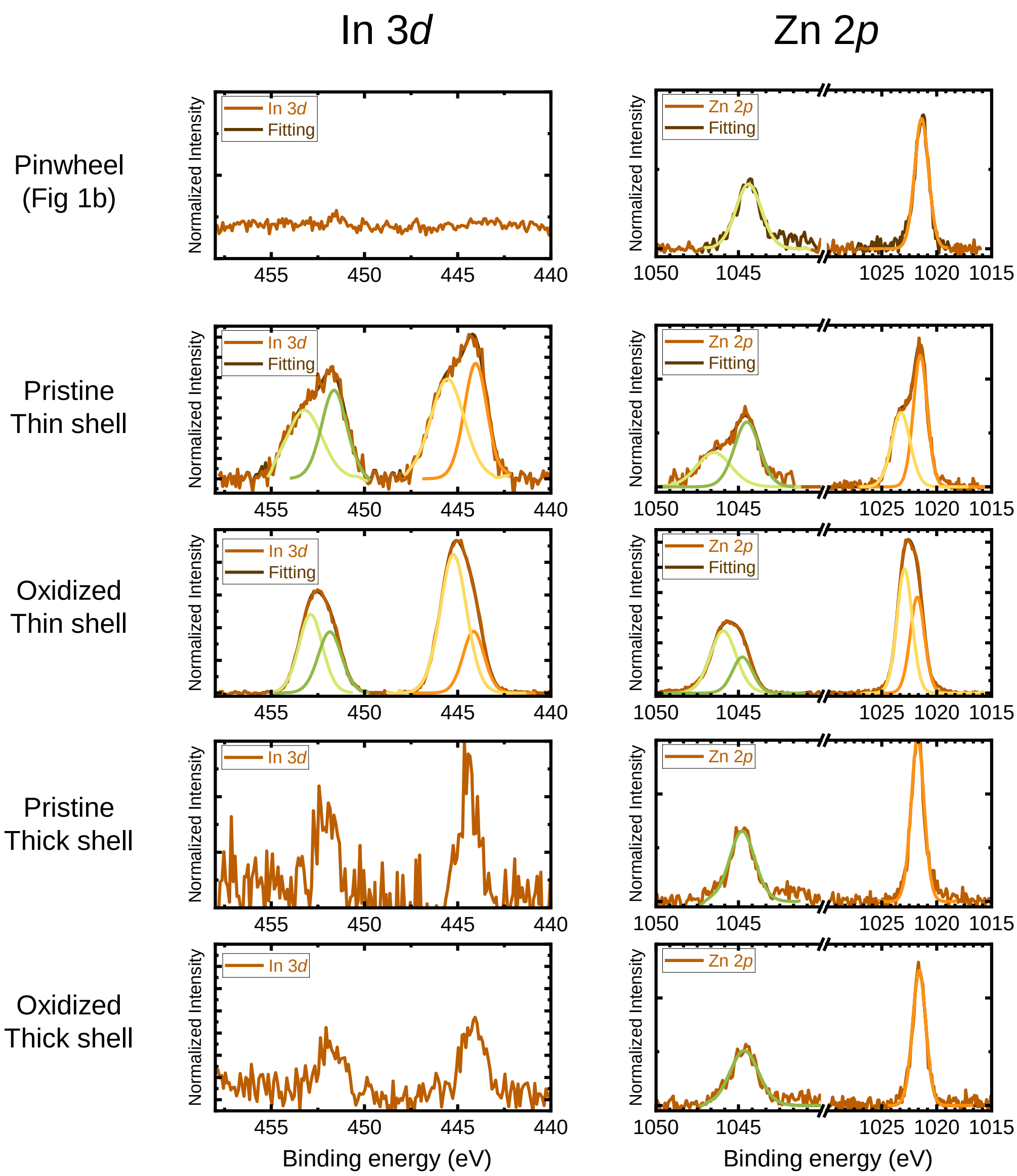

Supplementary Fig. 9. XPS spectra of In 3*d* and Zn 2*p* regions for pinwheel, pristine, and oxidized InAs/ZnSe core/shell CQDs with thin and thick shells. Left In 3*d* spectra; **right** Zn 2*p* spectra, with fitted components shown where resolvable. In the thick-shell samples and pinwheel one, the In 3*d* signal is largely obscured by noise due to the limited XPS probing depth.

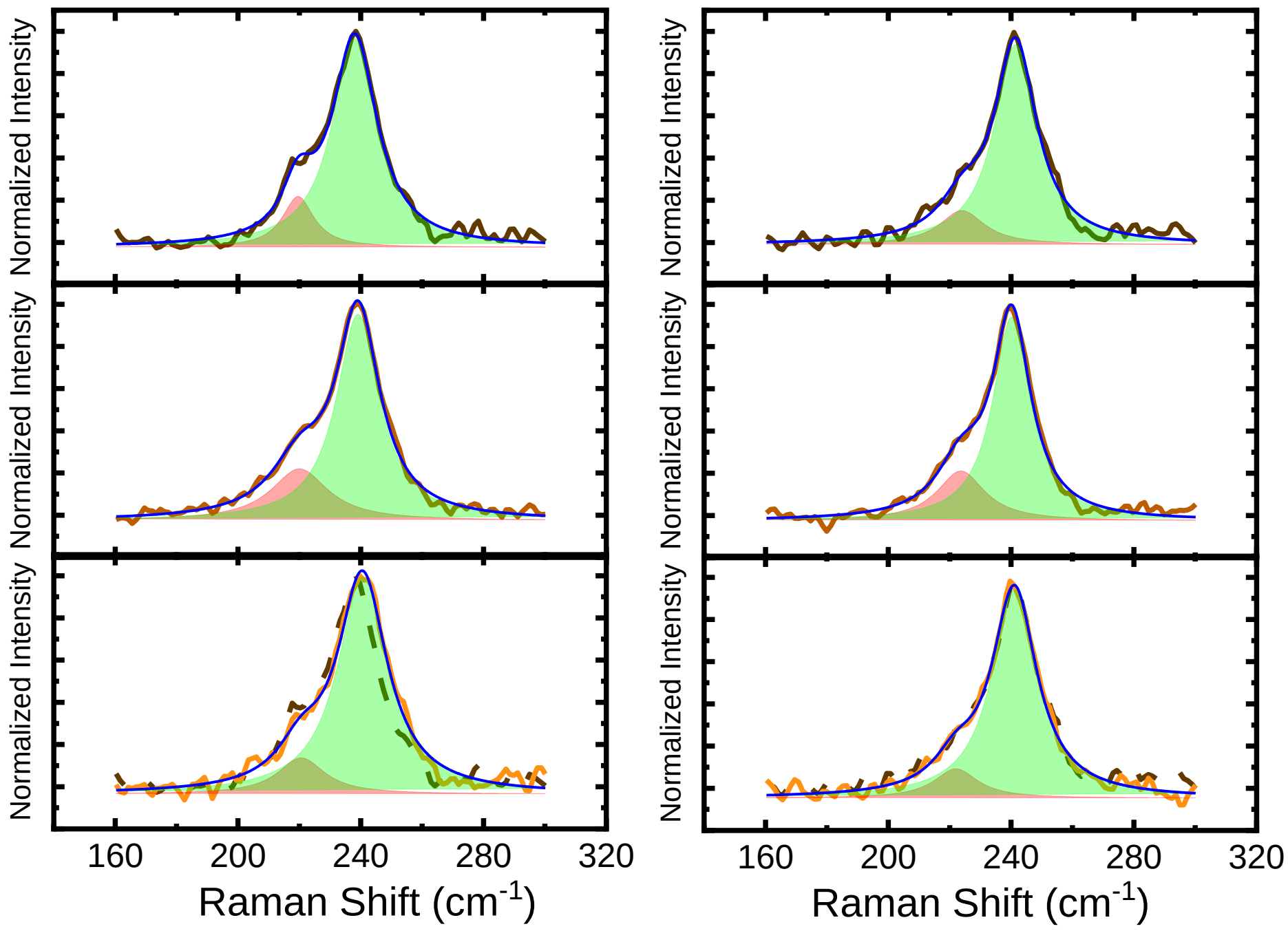

Supplementary Fig. 10. Two-component Lorentzian fitting of the InAs LO phonon mode for pristine and oxidized InAs/ZnSe core/shell CQDs. Raman spectra of pristine (left) and oxidized (right) core/shell CQDs with 1–2 ML (top), 4 ML (middle), and 7 ML (bottom) shell thickness, fit with two Lorentzian components (green, main LO component; pink, secondary component near 220 cm^{-1}) and their sum (blue). In the 7 ML panels, the corresponding 1–2 ML spectrum and fit (dashed) are overlaid for direct comparison, highlighting the shell-thickness-dependent spectral evolution in the pristine sample relative to the smaller changes observed in the oxidized sample. Extracted peak positions and FWHM values are summarized in Supplementary Table 1.

Supplementary Table 1. Lorentzian fitting parameters of the InAs LO phonon mode for pristine and oxidized InAs/ZnSe core/shell CQDs with varying shell thickness.

Sample	Shell thickness	LO peak center (cm^{-1})	LO FWHM (cm^{-1})
Pristine	1–2 ML	238.06	17.9
Pristine	4 ML	239.12	17.2
Pristine	7 ML	240.57	19.7
Oxidized	1–2 ML	241.44	16.8
Oxidized	4 ML	240.19	15.0
Oxidized	7 ML	241.08	17.9

Peak positions and FWHM values were obtained from Lorentzian fitting of the LO phonon region in the Raman spectra shown in Supplementary Fig. 10.

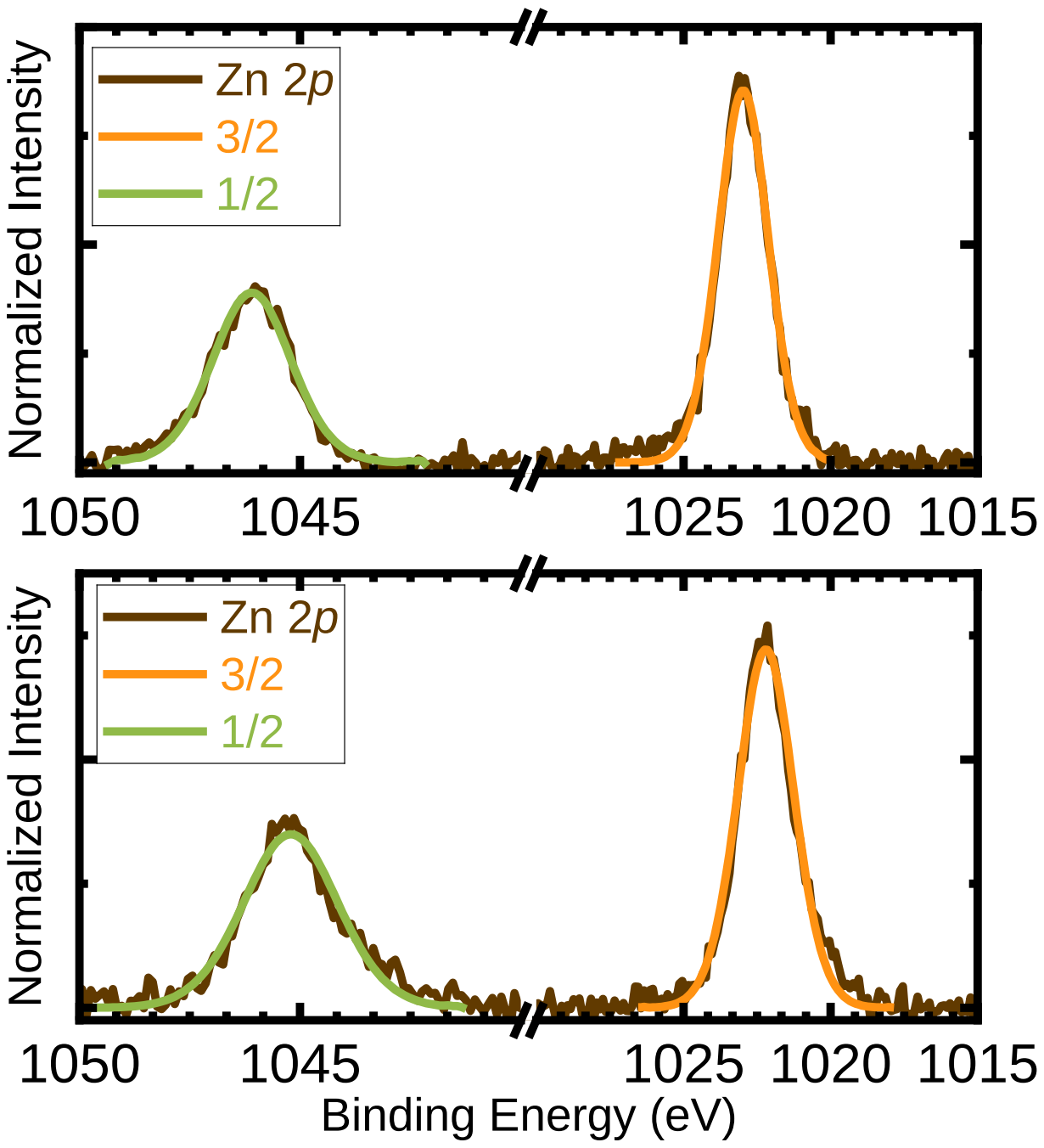

Supplementary Fig. 11. XPS spectra of the Zn 2*p* region for Zn-treated pristine and oxidized InAs cores prior to shell growth. Zn 2*p* spectra (brown) with fitted components (3/2, orange; 1/2, green) for the pristine (top) and oxidized (bottom) cores.

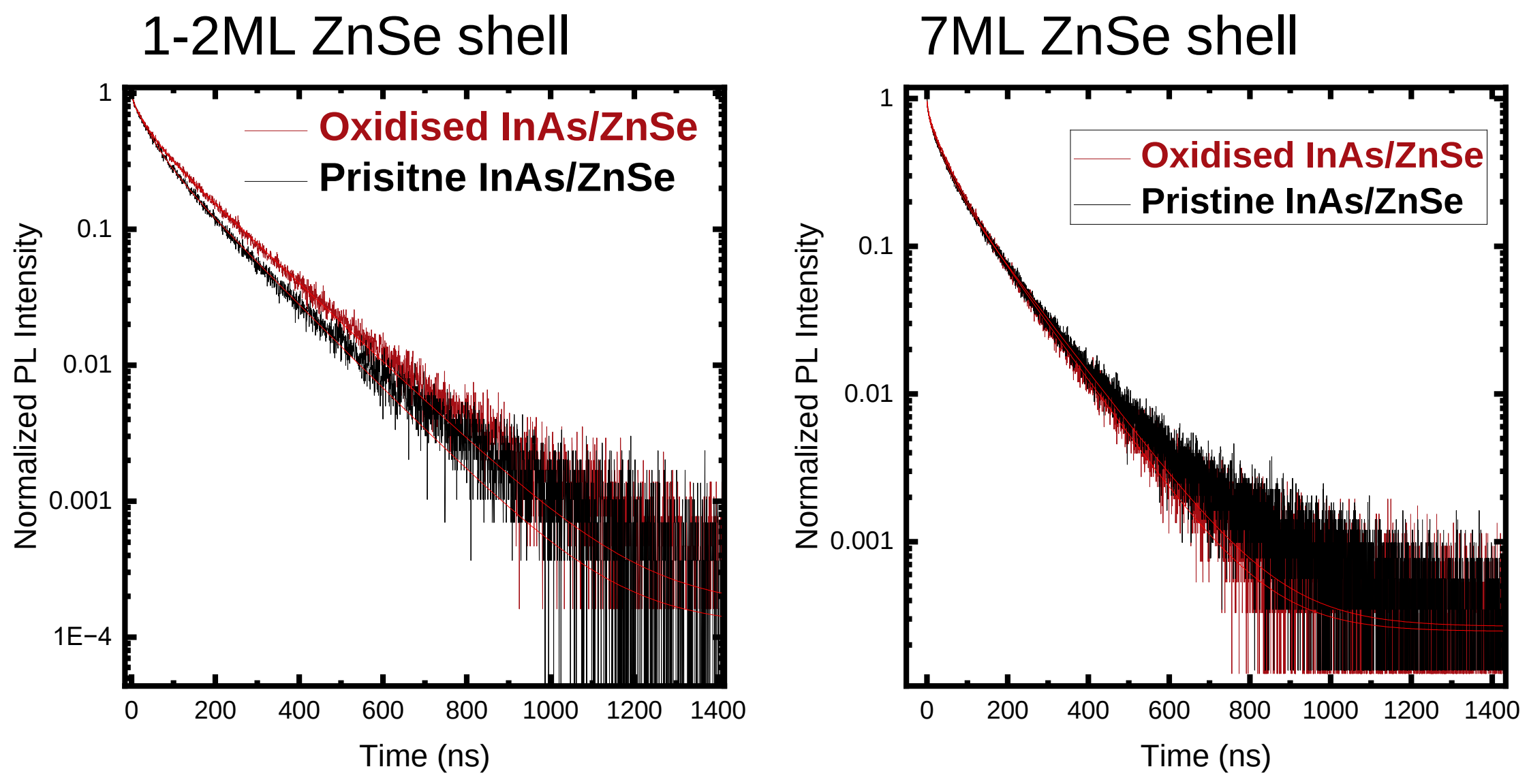

Supplementary Fig. 12. TRPL of pristine and oxidized-core/shell with thin (left) and thick (right) shell thickness

Supplementary Table 2 TRPL decay parameters of pristine- and oxidized-InAs/thin- and thick-ZnSe nanocrystals, corresponding to the data presented in Supplementary Figs. 5 and 8

PL time traces were fitted using a multi-exponential decay function:

$$y(t) = y_0 + \sum_i A_i e^{\frac{-t}{\tau}} \tag{1}$$

The amplitude-weighted average radiative lifetime was then calculated as:

$$\tau_{avg} = \frac{\sum_i A_i \tau_i^2}{\sum_i A_i \tau_i} \tag{2}$$

InAs/ZnSe	Wavelength (nm)	A_1	τ_1 (ns)	A_2	τ_2 (ns)	A_3	τ_3 (ns)	Average τ (ns)
Pristine-thin	1550	0.07	3.34	0.24	35.3	0.69	120.7	92.8
Oxidize-thin	1450	0.11	4.31	0.25	38.6	0.64	113.8	82.3
Pristine-thick	1550	0.005	3.97	0.206	43.1	0.789	140.4	119.4
Oxidize-thick	1410	0.005	4.43	0.111	35.7	0.883	149.4	135.6

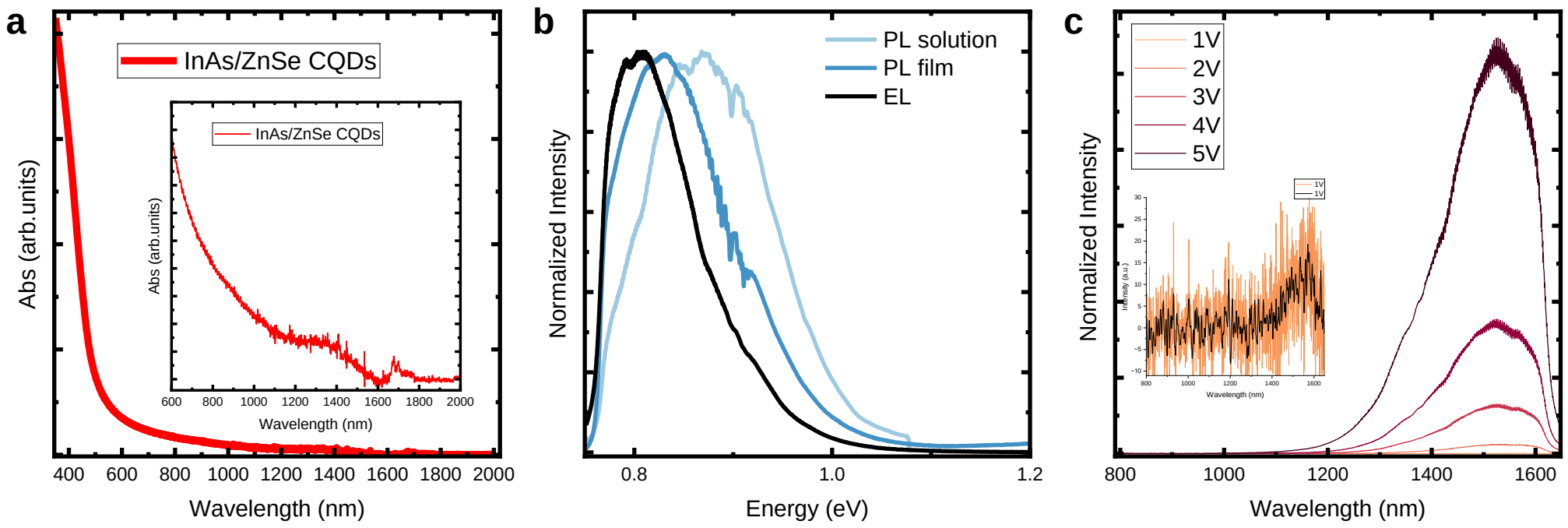

Supplementary Fig. 13 Optical and electroluminescence properties of InAs/ZnSe quantum dots and device operation. a UV-Vis absorption spectrum of InAs/ZnSe CQDs showing 3-4ML shell, and a well-defined first excitonic peak at ~1400 nm (inset). **b** Photoluminescence measurements in both solution (light blue spectrum) and solid-state film (blue spectrum) reveal a slight red shift of ~60 meV upon film formation, which is further reflected in the device electroluminescence (~20 meV shift, dark blue spectrum). **c** Bias-dependent EL measurements also show a small blue shift as the applied voltage increases, consistent with partial field-induced modulation of quantum dot emission within the device architecture.

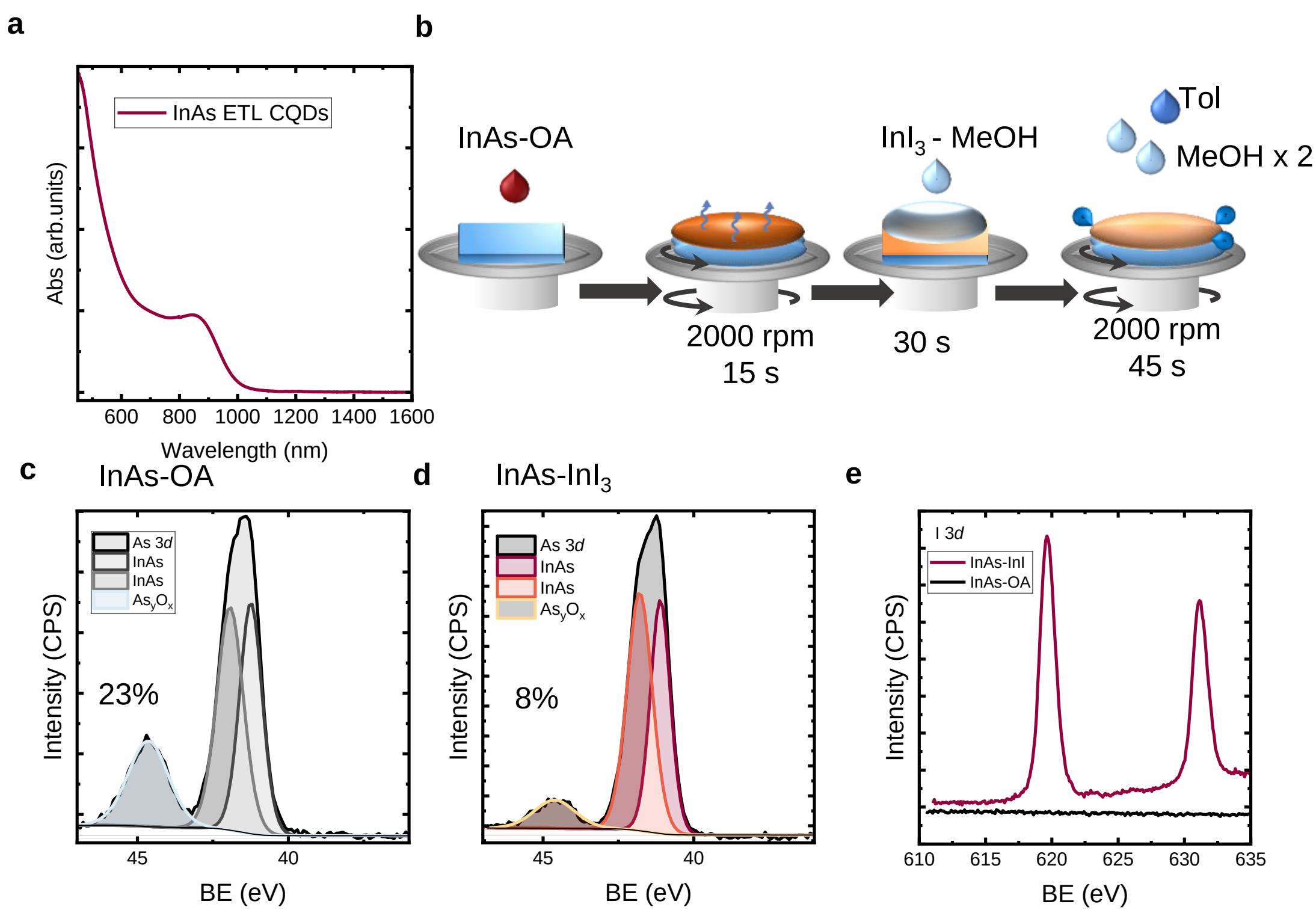

Supplementary Fig. 14 Optical properties of InAs-OA quantum dots and schematic of solid-state ligand exchange (SSLE). **a** UV-Vis absorption spectrum of InAs-OA nanocrystals in solution, showing a first excitonic absorption peak at ~850 nm; and **b** Schematic of the InI_3 solid-state ligand exchange (SSLE). OA-capped InAs CQDs are deposited by spin coating, exposed to the InI_3 solution for 30 s, and spin-rinsed with anhydrous methanol followed by toluene, resulting in a compact, conductive CQD film while preserving film integrity. **c-d** High-resolution As 3*d* XPS spectra of InAs-OA and InAs-InI_3 films. The oxidized arsenic (As-O) component at 44.5 eV is reduced from approximately 23% (c) to 8% (d), consistent with a successful resurfacing[1,2]; and **e** comparison of the I signal for InAs-OA (gray) and InAs-InI_3 (red), where XPS confirms the presence of iodine in the treated sample via peaks at 633 eV and 620 eV (I 3*d*), evidencing successful incorporation of iodine after SSLE treatment.

Supplementary Table 3. TRPL decay parameters of InAs/ZnSe nanocrystals on various ETLs

Underneath layer	A_1	τ_1 (ns)	A_2	τ_2 (ns)	A_3	τ_3 (ns)	A_4	τ_4 (ns)	Average τ (ns)
Al_2O_3	0.23	0.21	0.36	11.04	0.3	28.08	0.05	68.65	32.50
ZnO NPs	0.31	0.29	0.45	6.21	0.19	26.07			18.69
InAs ETL	0.19	0.49	0.54	10.98	0.22	43.32			30.73

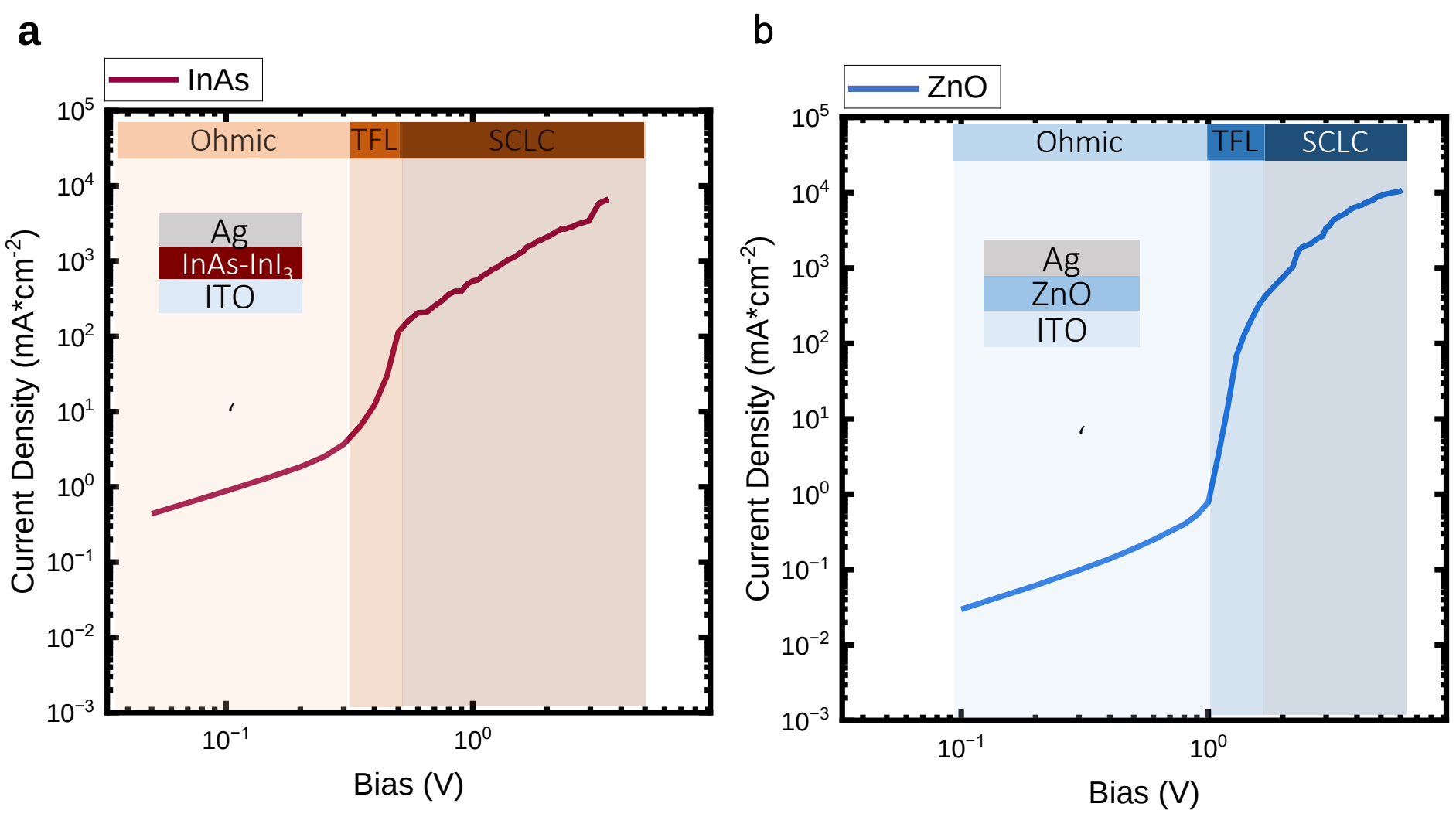

Supplementary Fig. 15 SCLC ZnO vs InAs. The extracted electron mobility of the InAs layer (**a**) is $1.3\cdot10^{-3}$ cm^2 V^{-1} s^{-1},[1,3,4] which is only modestly lower than that of the ZnO reference (**b**, $7.6\cdot10^{-3}$ cm^2 V^{-1} s^{-1}).[5] Trap densities were extracted from space-charge-limited current (SCLC) measurements using the trap-filled limit (TFL) relation:

$$N_t = \frac{2\varepsilon_0\varepsilon_r V_{TFL}}{qL^2} \tag{3}$$

assuming bulk dielectric constants for both materials (ε_r≈15 for InAs and ε_r≈9 for ZnO). Here, N_t is the trap density in cm^{-3}, ε_r is the relative permittivity of the material, ε_0 is the vacuum permittivity, V_{TFL} is the voltage at which traps are filled and the current becomes trap-free, *q* is the elementary charge, and L is the active layer thickness of the device. Using this approach, the InAs-InI_3 device (260 nm, V_{TFL}= 0.3 V) gave a trap density calculated to be $7.4\times10^{15}cm^{-3}$ while the ZnO NPs device (260 nm, V_{TFL}=0.95 V) exhibited a higher trap density of 1.4×10^{16} cm^{-3}. These results indicate that, under the assumption of bulk permittivity, the ZnO nanoparticle film contains a higher density of trap states compared to the InAs CQD layer.

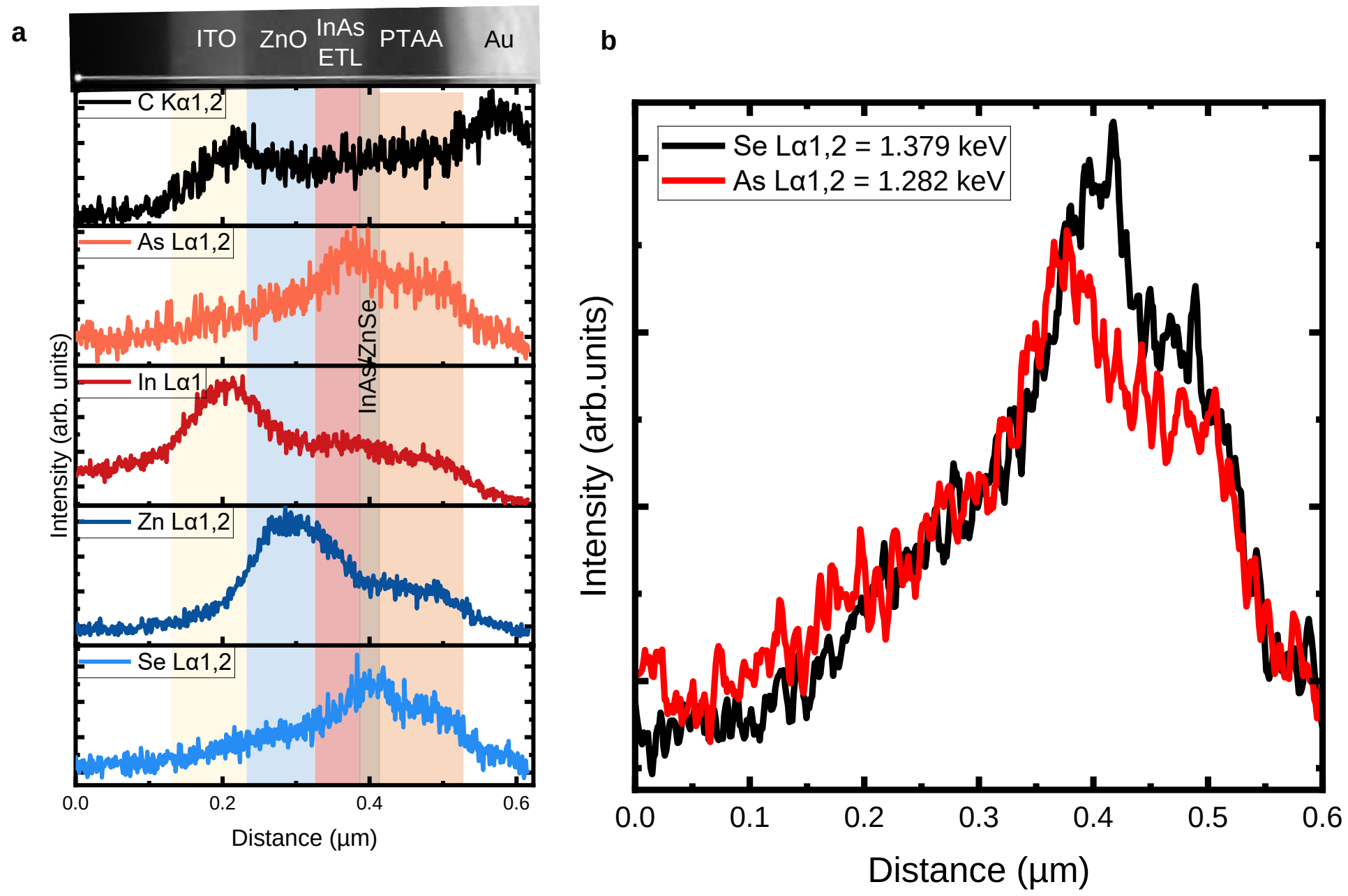

Supplementary Fig. 16 Cross-sectional elemental mapping of the device structure. **a** EDS line scan across the film thickness (distance in µm), showing the spatial distribution of C, As, In, Zn, and Se from the top to the bottom of the sample. The line profile clearly resolves the multilayer architecture, with the In signal identifying the ITO substrate at ~0.17-0.22 µm, the Zn signal peaking at ~0.29 µm corresponding to the ZnO layer (~130 nm), and the As signal localized to the InAs electron transport layer at ~0.32-0.36 µm. The Se signal, attributed to the ZnSe shell in the InAs/ZnSe quantum dots, is partially obscured by the close energy overlap with As Lα (~0.1 keV separation). **b** The deconvoluted profile in panel reveals a distinct contribution around ~0.42 µm, confirming the position of the InAs/ZnSe layer above the InAs ETL. Overlap of the Se Lα (1.379 keV) and As Lα (1.282 keV) signals highlighting their spectral proximity and partial overlap.

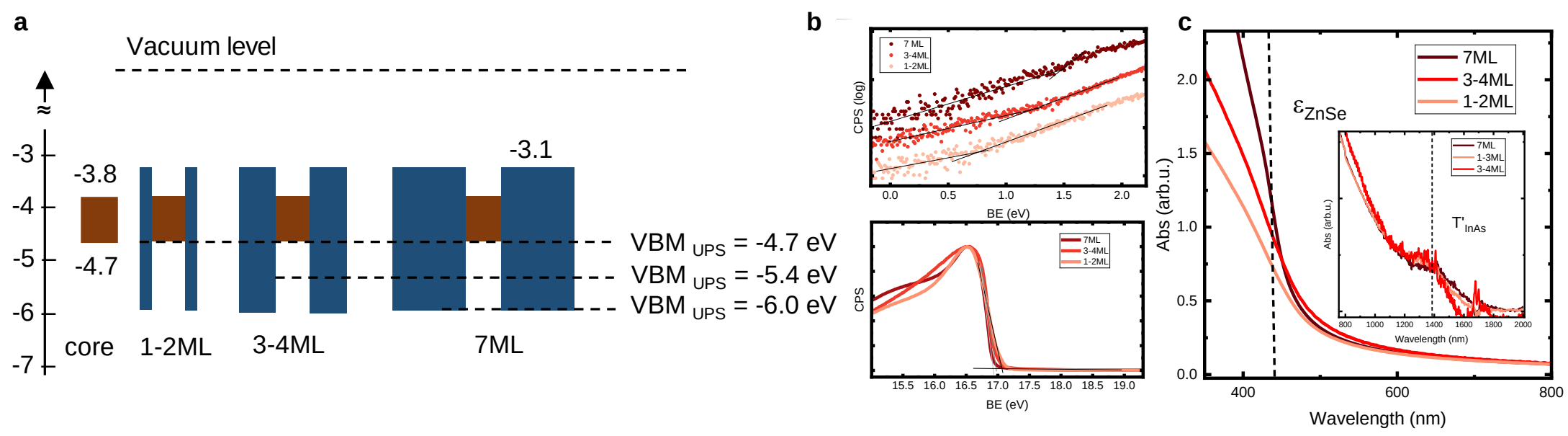

Supplementary Fig. 17 Evolution of the electronic structure with ZnSe shell thickness. a Schematic representation of bare InAs cores and InAs/ZnSe quantum dots with progressively increasing ZnSe shell thickness, illustrating the evolution of the valence band maximum (VBM). Thin shells (1-2 ML) closely resemble the electronic structure of the bare InAs core (VBM = -4.7 eV), intermediate shells (3-4 ML), corresponding to those used in the devices, exhibit mixed InAs/ZnSe character (VBM = -5.4 eV), whereas thick shells (7 ML) are dominated by the ZnSe electronic structure (VBM = -6.0 eV). These results indicate that the first ZnSe monolayer minimally perturbs the InAs core electronic structure, while increasing shell thickness progressively shifts the electronic response toward ZnSe-like behavior. **b** UPS spectra of bare InAs cores and InAs/ZnSe quantum dots with 1-2 (orange), 3-4 (red), and 7 ML (dark red) ZnSe shells, used to determine the VBM values shown in panel **a**. **c** Absorption spectra (350-800 nm) of InAs/ZnSe quantum dots with 1-2 ML (light orange), 3-4 ML (red), and 7 ML (dark red) ZnSe shells. All samples exhibit the exciton absorption peak at ~1400 nm (inset), together with a distinct ZnSe-related absorption feature at ~420 nm. This feature was used to estimate a ZnSe bandgap of ~2.9 eV, in good agreement with literature values.[6,7]

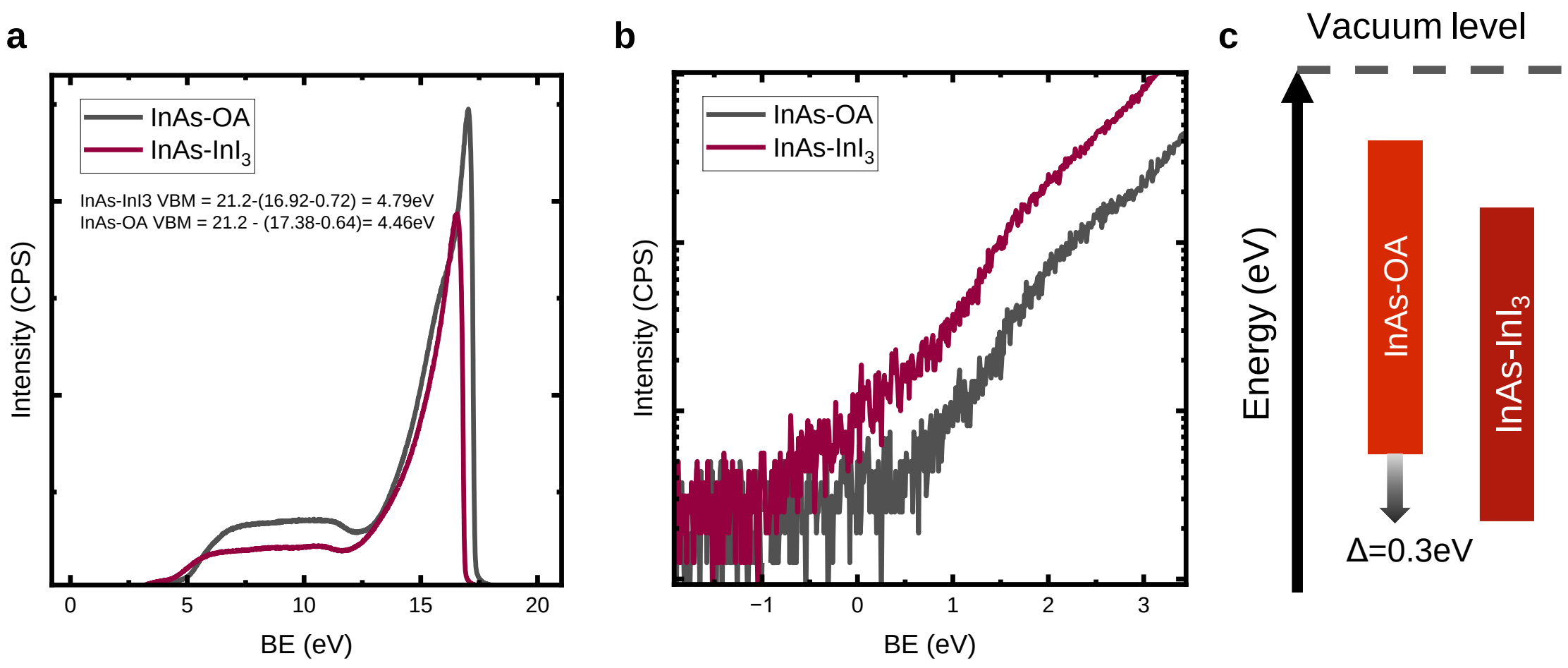

Supplementary Fig. 18 UPS characterization and energy level alignment of InAs quantum dots before and after surface treatment (SSLE) with InI_3. a UPS spectra of oleic acid-capped InAs (InAs-OA, black) and InI_3-treated InAs (InAs-InI_3, red); **b** low-binding-energy region of the UPS spectra (log-log scale) highlighting changes in the valence band edge; and **c** corresponding energy level schematic illustrating the VBM shift induced by SSLE. The InI_3 treatment produces a clear downshift of the valence band maximum from -4.4 ± 0.1 eV (InAs-OA) to -4.8 ± 0.1 eV (InAs-InI_3),[8] consistent with improved energy level alignment with the ZnO electron transport layer and the emissive layer.

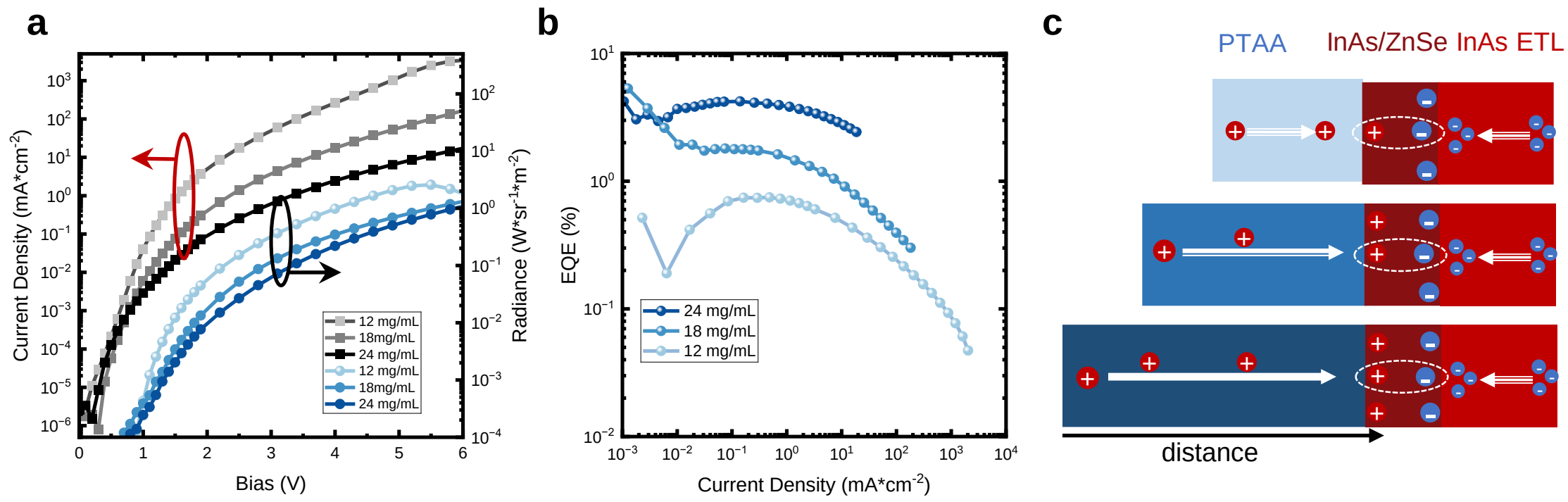

Supplementary Fig. 19 Effect of PTAA thickness on charge injection and device performance. a Current density–voltage–radiance (J-V-R) characteristics of devices with thick (~120 nm, 24 mg/mL) and thin (~60 nm) PTAA layers. Devices with thick PTAA operate in a transport-limited regime, exhibiting current densities of ~17 mA cm^{-2} (InAs-based) and radiance of ~1 W sr^{-1} m^{-2}. In contrast, reducing the PTAA thickness to ~60 nm significantly enhances charge injection, increasing the current density up to ~3000 mA cm^{-2} and radiance to ~2 W sr^{-1} m^{-2}. **b** External quantum efficiency (EQE) as a function of current density for devices with varying PTAA thickness. While thick PTAA devices achieve a higher peak EQE of 4.2%, thinner PTAA layers result in a reduced maximum EQE of 1.1% and pronounced efficiency roll-off, consistent with a transition toward electron over-injection at high current densities. **c** Schematic illustration of hole transport and injection as a function of PTAA thickness. Thinner PTAA layers enable faster hole transport but provide an insufficient hole flux relative to incoming electrons, leading to charge imbalance. Increasing the PTAA thickness improves hole injection balance by supplying a higher hole density, but at the cost of increased series resistance and reduced overall current.

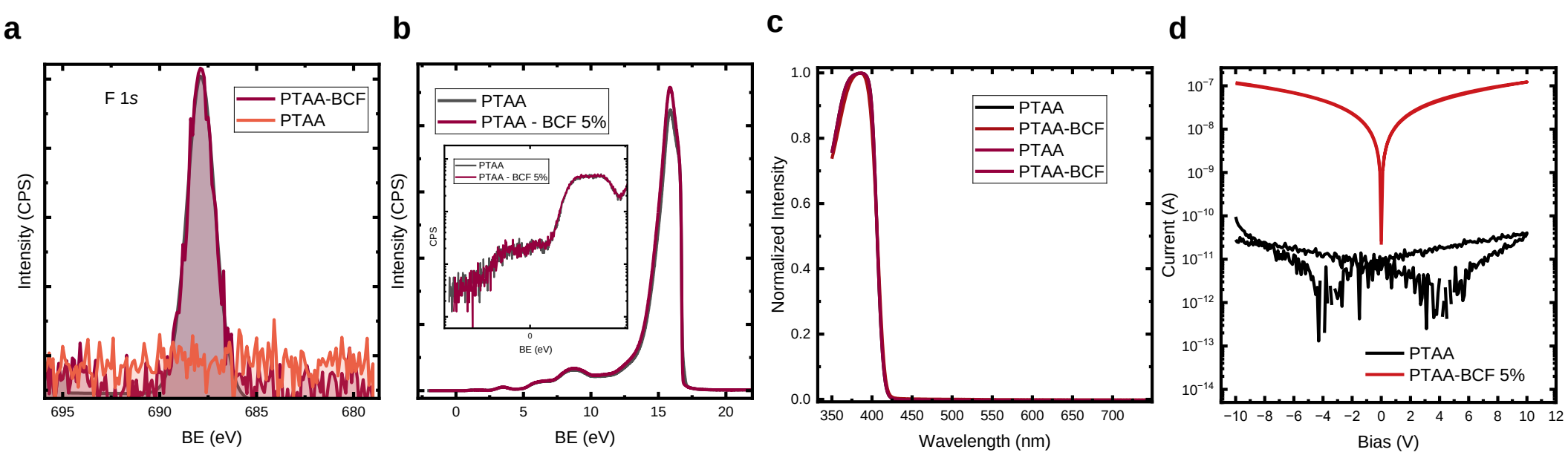

Supplementary Fig. 20 PTAA and PTAA-BCF characterizations. a XPS confirms successful incorporation of the dopant, as evidenced by the presence of the fluorine (F 1*s*) peak at ~687 eV, which is absent in undoped PTAA. **b** UPS and **c** optical absorption measurements confirm that BCF doping does not substantially modify the energy levels of PTAA, with the HOMO and LUMO remaining at approximately -5.1 ± 0.1 eV and -2.2 eV, respectively. **d** Lateral current–voltage (*I*–*V*) characteristics of pristine PTAA and PTAA-BCF films measured between two Au electrodes separated by a 10 µm gap. Both pristine PTAA and PTAA-BCF films exhibit linear and symmetric *I-V* characteristics over the measured voltage range, indicating ohmic charge transport. Linear fitting yields *I-V* slopes of $2.8 \cdot 10^{-12}$ and $9.6 \cdot 10^{-9}$ A V^{-1} for pristine PTAA and PTAA-BCF, respectively. Using the device geometry (L = 10 µm, W = 0.8 cm, t = 30 nm), the corresponding conductivities were calculated to be $1.17 \cdot 10^{-9}$ S cm^{-1} for pristine PTAA and $4 \cdot 10^{-6}$ S cm^{-1} for PTAA-BCF, corresponding to an enhancement of approximately three orders of magnitude upon BCF doping.[9]

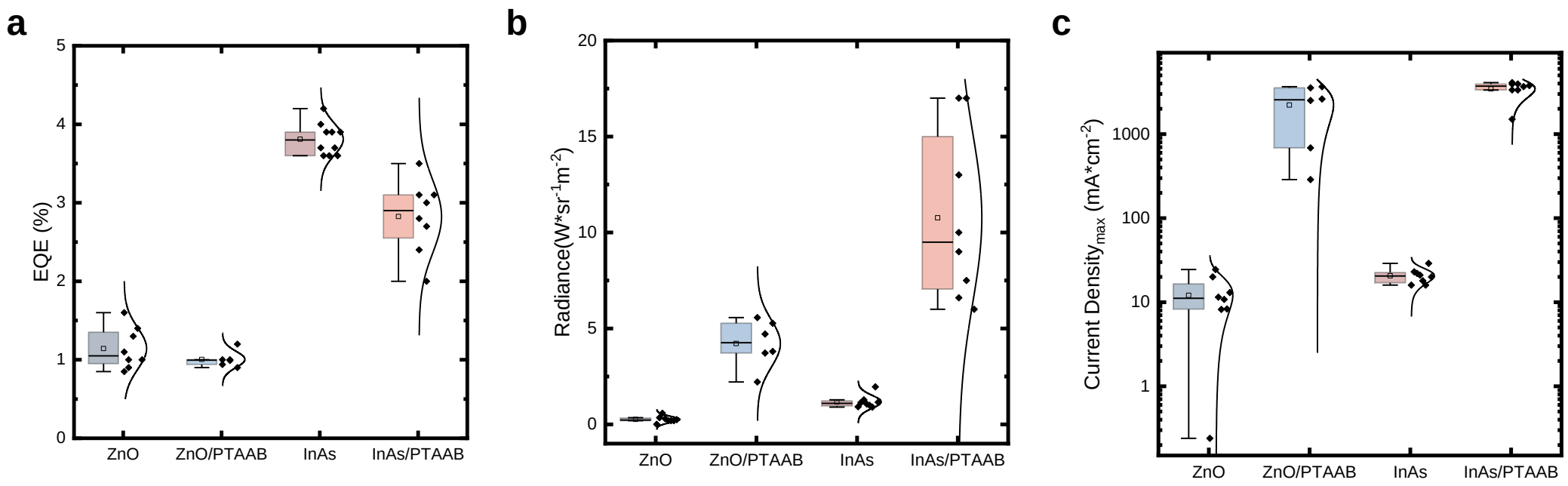

Supplementary Fig. 21 ZnO and ZnO/InAs ETLs with PTAA-BCF. a Box plots summarizing the external quantum efficiency (EQE) distributions show a slight decrease for ZnO-only devices, with a maximum EQE of 1.2% compared to 1.6% for the reference ZnO/InAs ETL devices. **b** Radiance distributions for devices employing ZnO-only and ZnO/InAs ETLs with either pristine PTAA or BCF-doped PTAA. In ZnO-only devices, the incorporation of doped PTAA enhances radiance, reaching up to 5.5 W sr^{-1} m^{-2} at a current density of 3200 mA cm^{-2}. **c** Current density distributions further highlight the improved charge injection achieved with PTAA doping, particularly in ZnO-only devices. Overall, the highest performance is consistently achieved in InAs-based devices, which exhibit superior EQE, radiance, and current density. These results indicate that while PTAA doping improves charge balance in ZnO-based devices, InAs-based architectures remain intrinsically more efficient.

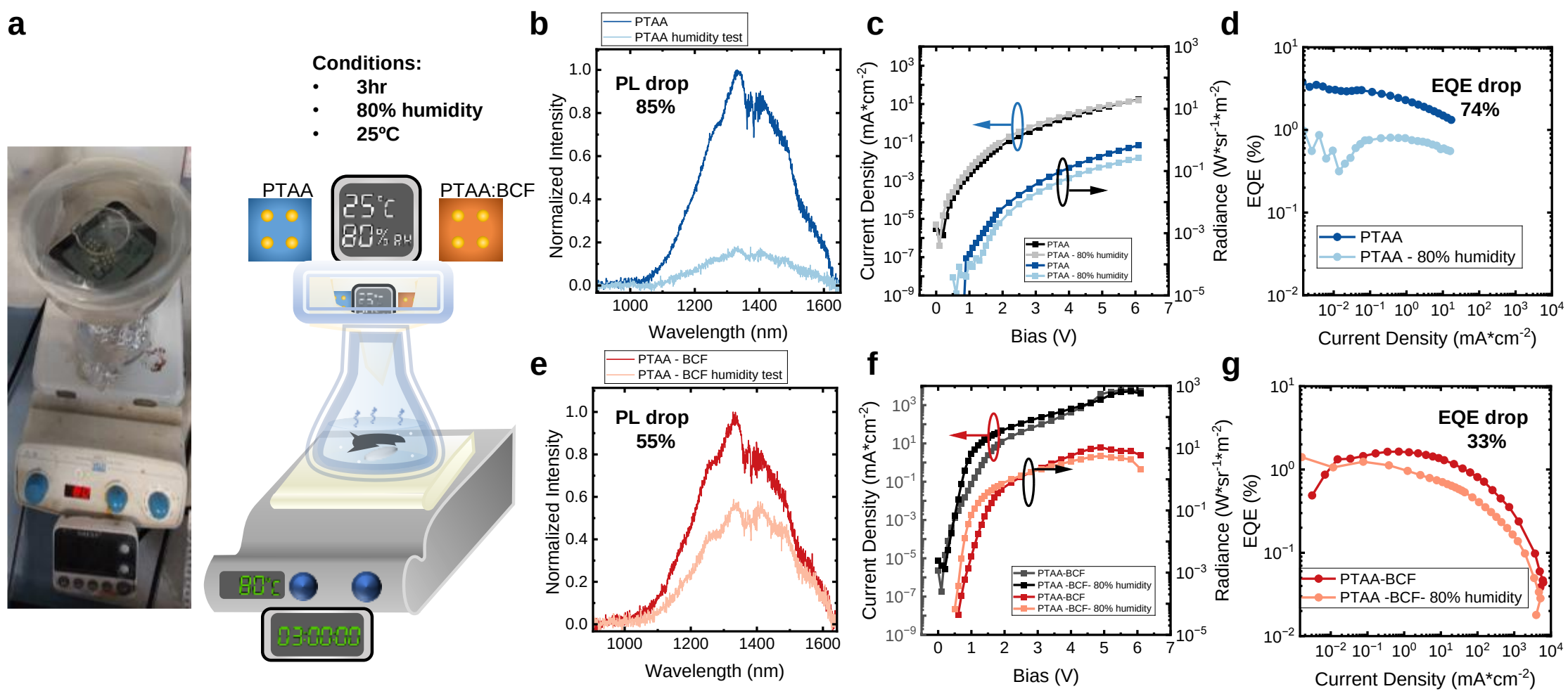

Supplementary Fig. 22 Accelerated moisture-aging test of PTAA and PTAA-BCF. a Photograph and schematic of the custom-built environmental chamber used for accelerated humidity aging (25 °C, 80% RH, 3 h). Devices were fabricated using InAs/ZnSe CQDs with 12 h oxidation, a 3-4 ML ZnSe shell, a PL peak at 1330 nm, and a solution PLQY of 33%. **b** Normalized PL spectra of InAs/ZnSe CQDs/PTAA films before and after humidity aging, normalized to the maximum PL intensity of the pristine sample. **c** *J-V-R* characteristics of PTAA-based LEDs before and after humidity aging. **d** EQE of PTAA before and after humidity aging (3.1% before and 0.8% after aging). **e** Normalized PL spectra of InAs/ZnSe CQDs/PTAA:BCF films before and after humidity aging, normalized to the maximum PL intensity of the pristine sample. **f** *J-V-R* characteristics of PTAA-BCFLEDs before and after humidity aging, and **g** the EQE before and after humidity aging (1.7% before and 1.1% after aging).

Supplementary Table 4 Comparison of maximum EQE in SWIR Light-emitting Diodes based on CQDs.

Emitting Material	EL (nm)	Peak Radiance W sr^{-1} m^{-2}	EQE (%)	Stability		Ref.
				T_{50} (h)	Radiance $_{Initial}$ (W sr^{-1} m^{-2})	
HgTe	1300	3	2.2	150*	3	10
HgTe	1400	20	0.25	80*	2	11
HgTe	2000	3	0.3	10	1	12
CdHgSe/ZnCdS	1550	0.2	3.8	-	-	13
PbS	1232	6.4	2	-	-	14
PbS	1242	0.75	4.3	-	-	15
PbS/CdS	1391	0.92	5.2	-	-	16
PbS	1400	9	7.9	48	9	17
PbS (blend)	1400	35	8.1	532	7	18
PbS	980	7.4	8.1	1.2	0.01	19
PbS	1550	10	11.8	-	-	20
Ag_2S - Perovskite matrix	1397	83.9	16.98	480 (T_{85})	83	21
AgAuSe	1046	1.51	15.8	-	-	22
InAs/ZnSE - MEHPPV	1270	-	0.5	-	-	23
In(Zn)As/In(Zn)P/GaP/ZnS	857	8.2	4.6	20 (T_{85})	8.2	24
InAs/ZnSe	947	0.15	5.5	32	0.15	25
In(Zn)As/In(Zn)P/GaP/ZnS	1006	2.2	13.3	20 (T_{85})	2.2	26

InAs/ZnSe	900	12	13.3	0.8	0.1	[27]
InAs/InP/ZnSe/ZnS	1033	581.4	20.4	550	50	[28]
InAs/ZnSe	1410	1	1.1	0.25	0.3	[29]
InAs/ZnSe	1525	17	3.8	16	4.4	**This work**

***** Maximum lifetime reported.

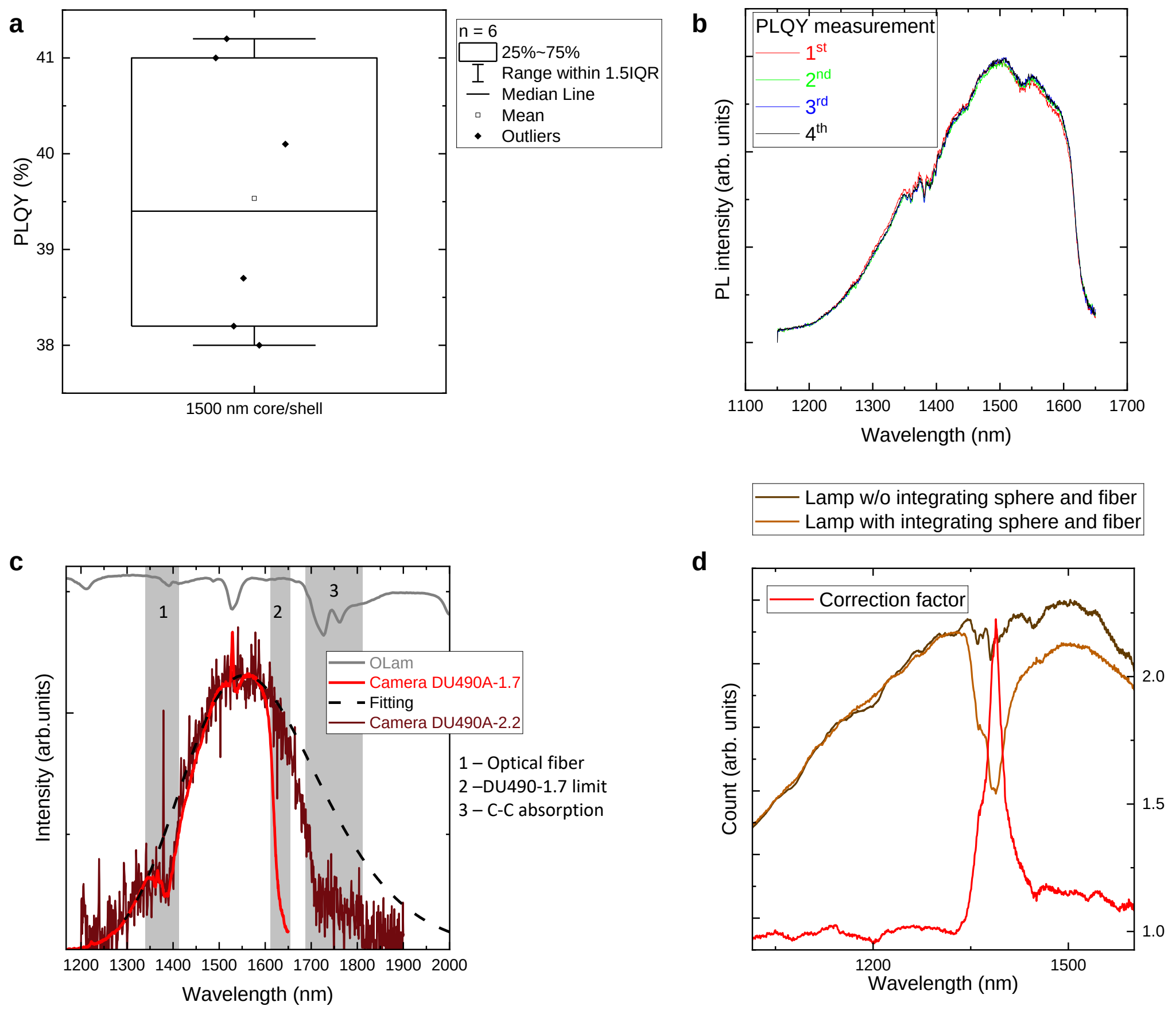

Supplementary Fig. 23 Reproducibility and validation of the PLQY measurements. a Batch-to-batch distribution of the PLQY obtained from six independently synthesized InAs/ZnSe core/shell CQD batches. The mean PLQY was approximately 39%. **b** Four consecutive PL measurements of the same InAs/ZnSe core/shell CQD sample acquired at 3 h intervals under identical conditions. The relative variation in the measured PLQY was below 1%. **c** Extended-range PL spectrum of the InAs/ZnSe core/shell CQDs measured using a detector covering wavelengths up to 2.2 μm, confirming the absence of substantial additional emission at longer wavelengths. The shaded regions indicate the spectral ranges affected by detector-response features, and the dashed line represents the fitted emission profile. **d** Broadband-lamp spectra measured using a reference optical path without the attenuation-inducing fiber and using the complete integrating-sphere/fiber configuration employed for the PLQY measurements. The wavelength-dependent correction factor was obtained from the ratio of the two measured spectra and used to correct the solution-phase PL spectra.

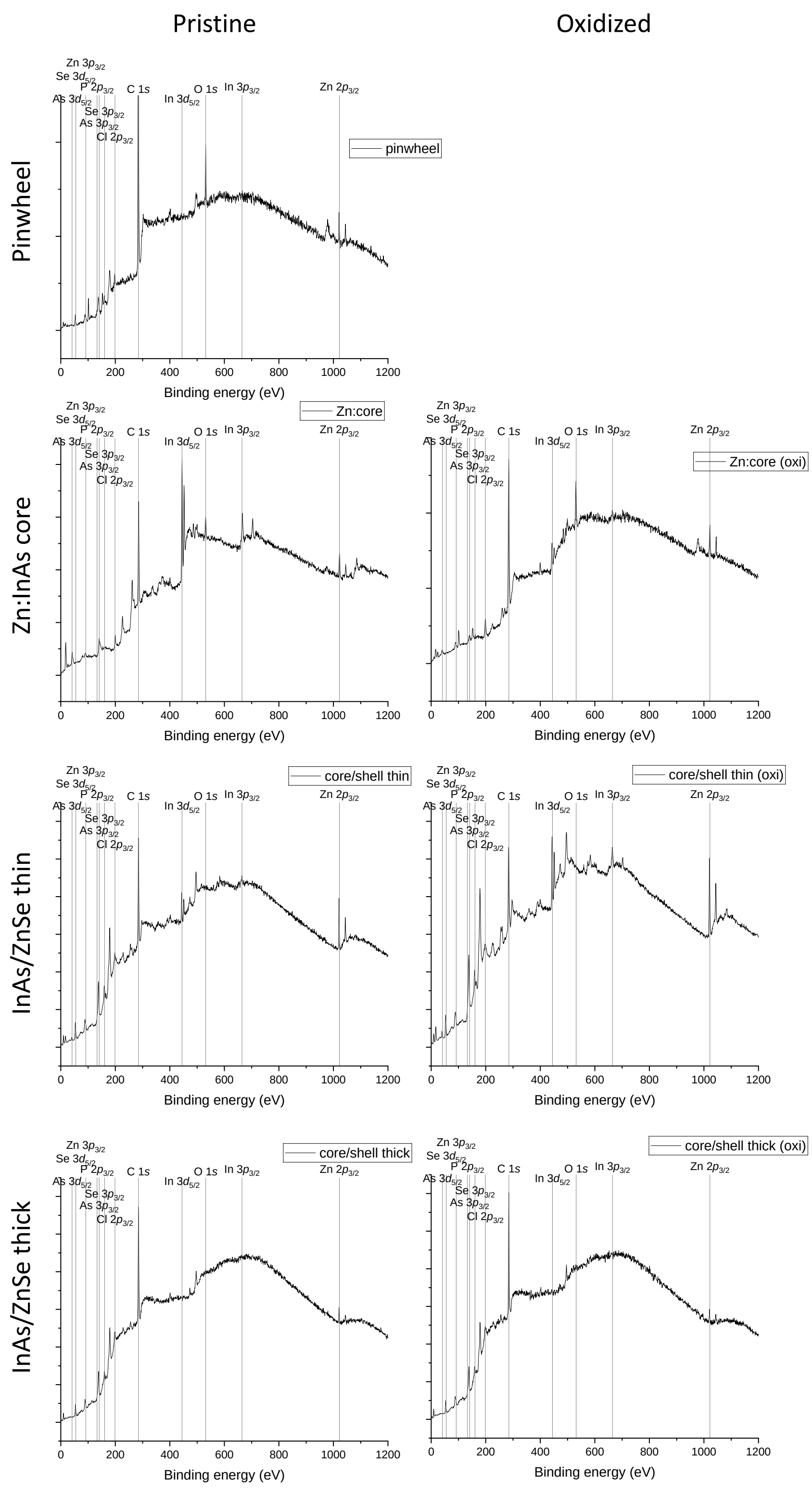

Supplementary Fig. 24. Full survey XPS spectra of Zn-treated InAs core, InAs/ZnSe core/thin- and thick shell. top Pinwheel and Zn-treated InAs cores **middle** thin-shell CQDs **bottom** thick-shell CQDs **left** pristine **right** oxidized.

Supplementary References

1. Sun, B. *et al.* Fast Near-Infrared Photodetection Using III–V Colloidal Quantum Dots. *Adv. Mater.* **34**, 2203039 (2022).
2. Jung, B. K. *et al.* High-Affinity Ligand-Enhanced Passivation of Group III–V Colloidal Quantum Dots for Sensitive Near-Infrared Photodetection. *ACS Energy Lett.* **9**, 504–512 (2024).
3. Leemans, J. *et al.* Colloidal III–V Quantum Dot Photodiodes for Short-Wave Infrared Photodetection. *Adv. Sci.* **9**, 2200844 (2022).
4. Ban, H. W. *et al.* Resurfacing of InAs Colloidal Quantum Dots Equalizes Photodetector Performance across Synthetic Routes. *J. Am. Chem. Soc.* **146**, 24935–24944 (2024).
5. Kirkwood, N., Singh, B. & Mulvaney, P. Enhancing Quantum Dot LED Efficiency by Tuning Electron Mobility in the ZnO Electron Transport Layer. *Adv. Mater. Interfaces* **3**, 1600868 (2016).
6. Al-Kuhaili, M. F., Kayani, A., Durrani, S. M. A., Bakhtiari, I. A. & Haider, M. B. Band Gap Engineering of Zinc Selenide Thin Films Through Alloying with Cadmium Telluride. *ACS Appl. Mater. Interfaces* **5**, 5366–5372 (2013).
7. Toufanian, R., Zhong, X., Kays, J. C., Saeboe, A. M. & Dennis, A. M. Correlating ZnSe Quantum Dot Absorption with Particle Size and Concentration. *Chem. Mater.* **33**, 7527–7536 (2021).
8. Song, J. H., Choi, H., Pham, H. T. & Jeong, S. Energy level tuned indium arsenide colloidal quantum dot films for efficient photovoltaics. *Nat. Commun.* **9**, 4267 (2018).
9. Ye, T. *et al.* Enhanced Efficiency of Planar Heterojunction Perovskite Solar Cells by a Light Soaking Treatment on Tris(pentafluorophenyl)borane-Doped Poly(triarylamine) Solution. *ACS Appl. Mater. Interfaces* **11**, 14004–14010 (2019).
10. Bossavit, E. *et al.* Optimized Infrared LED and Its Use in an All-HgTe Nanocrystal-Based Active Imaging Setup. *Adv. Opt. Mater.* **10**, 2101755 (2022).
11. Prado, Y. *et al.* Seeded Growth of HgTe Nanocrystals for Shape Control and Their Use in Narrow Infrared Electroluminescence. *Chem. Mater.* **33**, 2054–2061 (2021).

12. Qu, J. *et al.* Electroluminescence from nanocrystals above 2 µm. *Nat. Photonics* **16**, 38–44 (2022).

13. Roshan, H. *et al.* Short-Wave Infrared Optoelectronics with Colloidal CdHgSe/ZnCdS Core/Shell Nanoplatelets. *ACS Photonics* **12**, 40–47 (2025).

14. Sun, L. *et al.* Bright infrared quantum-dot light-emitting diodes through inter-dot spacing control. *Nat. Nanotechnol.* **7**, 369–373 (2012).

15. Supran, G. J. *et al.* High-Performance Shortwave-Infrared Light-Emitting Devices Using Core–Shell (PbS–CdS) Colloidal Quantum Dots. *Adv. Mater.* **27**, 1437–1442 (2015).

16. Gong, X. *et al.* Highly efficient quantum dot near-infrared light-emitting diodes. *Nat. Photonics* **10**, 253–257 (2016).

17. Pradhan, S. *et al.* High-efficiency colloidal quantum dot infrared light-emitting diodes via engineering at the supra-nanocrystalline level. *Nat. Nanotechnol.* **14**, 72–79 (2019).

18. Pradhan, S., Dalmases, M., Baspinar, A.-B. & Konstantatos, G. Highly Efficient, Bright, and Stable Colloidal Quantum Dot Short-Wave Infrared Light-Emitting Diodes. *Adv. Funct. Mater.* **30**, 2004445 (2020).

19. Gao, L. *et al.* Efficient near-infrared light-emitting diodes based on quantum dots in layered perovskite. *Nat. Photonics* **14**, 227–233 (2020).

20. Pradhan, S., Dalmases, M., Taghipour, N., Kundu, B. & Konstantatos, G. Colloidal Quantum Dot Light Emitting Diodes at Telecom Wavelength with 18% Quantum Efficiency and Over 1 MHz Bandwidth. *Adv. Sci.* **9**, 2200637 (2022).

21. Vasilopoulou, M. *et al.* Efficient colloidal quantum dot light-emitting diodes operating in the second near-infrared biological window. *Nat. Photonics* **14**, 50–56 (2020).

22. Ma, Z. *et al.* Interface-Mediation-Enabled High-Performance Near-Infrared AgAuSe Quantum Dot Light-Emitting Diodes. *J. Am. Chem. Soc.* **145**, 24972–24980 (2023).

23. Tessler, N., Medvedev, V., Kazes, M., Kan, S. & Banin, U. Efficient Near-Infrared Polymer Nanocrystal Light-Emitting Diodes. *Science* **295**, 1506–1508 (2002).

24. Wijaya, H. *et al.* Efficient Near-Infrared Light-Emitting Diodes based on In(Zn)As–In(Zn)P–GaP–ZnS Quantum Dots. *Adv. Funct. Mater.* **30**, 1906483 (2020).

25. De Franco, M. *et al.* Near-infrared light-emitting diodes based on RoHS-compliant InAs/ZnSe colloidal quantum dots. *ACS Energy Lett.* **7**, 3788–3790 (2022).

26. Zhao, X., Lim, L. J., Ang, S. S. & Tan, Z.-K. Efficient Short-Wave Infrared Light-Emitting Diodes Based on Heavy-Metal-Free Quantum Dots. *Adv. Mater.* **34**, 2206409 (2022).

27. Roshan, H. *et al.* Near Infrared Light-Emitting Diodes Based on Colloidal InAs/ZnSe Core/Thick-Shell Quantum Dots. *Adv. Sci.* **11**, 2400734 (2024).

28. Li, B. *et al.* Efficient and stable near-infrared InAs quantum dot light-emitting diodes. *Nat. Commun.* **16**, 2450 (2025).

29. Roshan, H. *et al.* Short-Wave Infrared InAs Quantum-Dot Light-Emitting Diodes with Tunable Electroluminescence beyond 1.4 μm. *ACS Energy Lett.* **11**, 3764–3770 (2026)
