# Supplementary material for: Controlling the shell growth mechanism and interfacial oxidation of InAs/ZnSe quantum dots for shortwave infrared emission
Source: Nat Commun. 2026 Oct 19;17:9969. doi: 10.1038/s41467-026-77896-0 (PMC13680504; doi:10.1038/s41467-026-77896-0)
Supplement: Supplementary file 2 [file 41467_2026_77896_MOESM2_ESM.pdf]

nature portfolio

Peer Review File

Controlling the Shell Growth Mechanism and Interfacial Oxidation of InAs/ZnSe Quantum Dots for Shortwave Infrared Emission

Corresponding Author: Professor Gerasimos Konstantatos

This file contains all reviewer reports in order by version, followed by all author rebuttals in order by version.

Version 0:

Reviewer comments:

Reviewer #1

(Remarks to the Author)
This manuscript reports a breakthrough in heavy-metal-free SWIR LEDs, achieving >55% PLQY at ~1400 nm and record EQE /radiance at 1525 nm using an ambient-oxidation-derived amorphous oxide buffer at the InAs/ZnSe interface. While the performance is impressive, the structural and mechanistic claims lack sufficient direct evidence. Major revision is required before reconsideration. Detailed comments are listed below:
1. The term "highly efficient" used in the title is rather strong, and a more moderate expression would be preferable. On page 3, the authors describe the architecture as "strain-free" and claim it reduces the PL FWHM. However, evidence supporting the complete absence of strain should be provided, as a strain-free state appears unusual. Furthermore, the FWHM reduction shown in Table 1 is not significant. These two points are representative; the manuscript contains many claims that lack sufficient experimental support, and the authors should carefully review the entire manuscript.
2. The inverse-oriented island growth model proposed on page 7 is an interesting hypothesis, but it currently lacks direct experimental evidence. Since this model is central to explaining the role of the surface oxide, which is the key innovation of this work, clear experimental validation is essential.
3. The current oxidation method relies on exposing samples to ambient air for a set duration, which is sensitive to many factors such as purification procedures, storage conditions, and humidity. Establishing a standardized and reproducible oxidation protocol would greatly improve the reliability of the results.
4. Although the oxide layer enhances the PLQY, the manuscript does not mention experimental errors. Multiple replicate measurements with error bars should be included to convincingly demonstrate this improvement.
5. In Figure 3h, the current density is nearly identical for devices with and without the InAs ETL at the same bias. Given that InAs ETLs typically exhibit modest conductivity and here have a substantial thickness of 40 nm, this observation is kind of surprising and requires an explanation.
6. In Figure 4a, doping PTAA with merely 5% BCF increases the current density by over two orders of magnitude at 6 V. Since the overall device conductivity is likely limited by the poorly conductive QD emissive layer and the InAs ETL, such a dramatic enhancement solely from PTAA doping is unexpected and warrants a detailed explanation.
7. Please provide the T50 lifetime at defined current density and radiance instead of T66. Besides, independent verification of the reported EQE values would strengthen the claims.
8. On page 11, the improved operational stability is attributed to reduced moisture uptake and dopant migration, but these arguments are drawn from perovskite device literature. Direct evidence specific to this QD-LED system should be provided to explain the enhanced lifetime.

Reviewer #2

(Remarks to the Author)
The manuscript describes an air oxidation strategy for improving SWIR emission from InAs/ZnSe QDs. The authors propose that oxidation of the InAs core forms an amorphous interfacial oxide layer that relieves strain during ZnSe shell growth, leading to higher PLQY beyond 1400 nm, and they further apply these emitters in QLEDs near 1550 nm. The PLQY of the QDs and related device performance is nice. However, the mechanistic discussion of how the oxide layer improves the PLQY remains rather preliminary and several key results and data analyses are not sufficiently convincing. The manuscript does not meet the high standard expected for publication in Nature Communications.

1. There are several inconsistencies in the manuscript that should be corrected. For example, Supplementary Fig. 2 appears to use an oxidation time of 36 h, whereas the main text refers to oxidation for more than 48 h. In addition, the figure citation in the main text should be carefully checked, since the discussion of the absorption shift appears to refer to Fig. 1b rather than Fig. 1c.
2. The authors state that the relatively high PLQY of the pristine InAs/ZnSe QDs is due to the continuous injection protocol and precise temperature control. Since the PLQY difference between the pristine and oxidized samples is not very large in some cases, these synthetic parameters should be investigated more carefully.
3. The PL spectra in Fig. 1d and 1e appear to be strongly affected by solvent absorption in the SWIR region. The manuscript does not clearly explain whether the PLQY measurements were influenced by this absorption, or whether fitted PL spectra were used for PLQY calculation. This raises concerns about the reliability of the reported PLQY values. The authors should optimize the PL measurements, provide proper solvent correction or control experiments, and present more reliable PL spectra. The current spectral shapes are not convincing for a main figure in Nature Communications.
4. The electron microscopy and elemental mapping results in Fig. 2a and 2b are difficult to interpret. In particular, in Fig. 2b oxygen appears to be distributed across the whole particle, whereas Zn seems to be located mainly near the upper surface. These maps do not clearly support the proposed interfacial oxide model. The authors should also explain in much more detail how the XPS data in Fig. 2c support their conclusion about Zn coordination, rather than relying mainly on literature comparison. In addition, Se elemental mapping is missing from Fig. 2, which makes the discussion of the ZnSe shell incomplete.
5. The statement that “the interfacial oxide layer suppressed interfacial alloying while maintaining a similar shell thickness” requires a clearer explanation and stronger evidence. The elemental maps in Supplementary Fig. 12 are also confusing. For example, In appears to be distributed throughout the whole particle, and As seems to appear near the particle surface. These observations should be carefully explained.
6. The Raman data in Fig. 2e show only a very small shift, around 1 cm^{-1}, between the two samples. It is unclear whether such a small difference is sufficient to support the proposed strain relaxation mechanism. Moreover, the XRD peaks of the InAs/ZnSe samples appear to shift to higher angles. The authors should clarify whether ZnSe shell growth is epitaxial in both samples and whether the InAs core is compressed in either case.
7. The proposed growth model in Fig. 2f remains speculative. The authors should provide high resolution TEM, STEM, or other structural characterization to support the proposed inverse oriented growth model and the claimed interfacial structure.
8. In the LED section, the authors should clearly state the size of the InAs/ZnSe QDs used in the devices and the corresponding ZnSe shell thickness.
9. The device discussion should be updated in light of recent studies on water treated ZnMgO electron transport layers and positive aging in QLEDs. These works suggest that the role of oxide electron transport layers, electron injection, and interfacial aging may be more complex than the conventional picture of excessive electron injection. The pronounced positive aging behavior observed in Fig. 4d should also be discussed in this context.
10. The energy level diagram in Fig. 3e should be reorganized. In its current form, the energy alignment and interfacial barriers are not sufficiently clear.
11. In Supplementary Fig. 14, the authors state that oleic acid binds more weakly to the InAs QD surface than oleylamine. This appears to contradict previous reports. The authors should provide direct evidence or appropriate references to support this statement.
12. In Supplementary Fig. 15, the authors should explain why InI_3 treatment removes surface oxides from the InAs QDs. The chemical basis for this claim is not clear, and additional characterization would be needed to support it.

Reviewer #3

(Remarks to the Author)
See attached report.

Version 1:

Reviewer comments:

Reviewer #1

(Remarks to the Author)
The revisions are satisfactory. I recommend the acceptance of this manuscript for publication at Nature Comm.

Reviewer #2

(Remarks to the Author)
The authors have addressed all my concerns and the manuscript has been substantially improved accordingly. I thus recommend the manuscript to be accepted for publication in Nature Communications.

Reviewer #3

(Remarks to the Author)
The authors have made substantial efforts to address my concerns. Especially the extra work for establishing the robustness of their PLQY measurements and the streamlined XPS vs. XRD experiments are noteworthy. I can now recommend the manuscript for publication in Nature Comm.

Open Access This Peer Review File is licensed under a Creative Commons Attribution 4.0 International License, which permits use, sharing, adaptation, distribution and reproduction in any medium or format, as long as you give appropriate credit to the original author(s) and the source, provide a link to the Creative Commons license, and indicate if changes were made.
In cases where reviewers are anonymous, credit should be given to 'Anonymous Referee' and the source.
The images or other third party material in this Peer Review File are included in the article's Creative Commons license, unless indicated otherwise in a credit line to the material. If material is not included in the article's Creative Commons license and your intended use is not permitted by statutory regulation or exceeds the permitted use, you will need to obtain permission directly from the copyright holder.
To view a copy of this license, visit https://creativecommons.org/licenses/by/4.0/

Controlling the Shell Growth Mechanism and Interfacial Oxidation of InAs/ZnSe Quantum Dots for Shortwave Infrared Emission (Previous title: Oxidation-Induced Interface Engineering in InAs/ZnSe Core/Shell Quantum Dots for Highly Efficient Shortwave Infrared Emission)

Taewan Kim[§], Manuela De Franco[§], Miguel Dosil, Jae Taek Oh, Rajesh Bera, Marta Martos Valverde, Gaurav Kumar & Gerasimos Konstantatos[*]

===
Editorial comments:
===

REVIEWER COMMENTS

===
Reviewer 1 Comments to the Author
===

This manuscript reports a breakthrough in heavy-metal-free SWIR LEDs, achieving >55% PLQY at ~1400 nm and record EQE /radiance at 1525 nm using an ambient-oxidation-derived amorphous oxide buffer at the InAs/ZnSe interface. While the performance is impressive, the structural and mechanistic claims lack sufficient direct evidence. Major revision is required before reconsideration. Detailed comments are listed below:

Author response: We thank the reviewer for recognizing the significance of the reported SWIR LED performance and for the constructive assessment of the structural and mechanistic aspects of our study. In response, we have substantially revised the manuscript and Supplementary Information to strengthen the experimental basis of our interpretation. We added and expanded structural, spectroscopic, and control analyses, clarified the respective roles of the EELS, XPS, XRD, Raman, and TRPL results, and moderated mechanistic statements where direct identification was not possible. We also added further details regarding the oxidation conditions, synthesis reproducibility, and optical-measurement validation. These revisions more clearly distinguish direct experimental observations from the proposed mechanistic interpretation. Our detailed responses to each comment are provided below.

1. The term "highly efficient" used in the title is rather strong, and a more moderate expression would be preferable. On page 3, the authors describe the architecture as "strain-free" and claim it reduces the PL FWHM. However, evidence supporting the complete absence of strain should be provided, as a strain-free state appears unusual. Furthermore, the FWHM reduction shown in Table 1 is not significant. These two points are representative; the manuscript contains many claims that lack sufficient experimental support, and the authors should carefully review the entire manuscript.

Author response to comment 1: We sincerely appreciate the reviewers for their meticulous review and constructive feedback. Based on the additionally obtained EELS and TEM images, we have investigated the initial growth mechanism of the InAs/ZnSe core/shell structure, as well as the specific characteristics of the continuous injection process and $ZnCl_2$ degassing, thereby improving the overall quality of the manuscript. Accordingly, to reflect our findings more accurately and address the concern regarding the strong phrasing in the title, we have revised the title to "**Controlling the Shell Growth Mechanism and Interfacial Oxidation of InAs/ZnSe Quantum Dots for Shortwave Infrared Emission**."
Furthermore, we have thoroughly reviewed the entire manuscript to provide the lacking experimental support for our claims. We have also carefully corrected the drafting errors and inconsistencies pointed out by the reviewers, including the oxidation time and figure citations. For example, we have also removed the claim regarding FWHM reduction, as the reviewer correctly noted that the data do not support a significant narrowing upon oxidation. The FWHM values in Table 1 were recalculated from the spectrally corrected experimental PL spectra and are now reported without any accompanying claim of linewidth narrowing. Please refer to our detailed point-by-point responses below.

2. The inverse-oriented island growth model proposed on page 7 is an interesting hypothesis, but it currently lacks direct experimental evidence. Since this model is central to explaining the role of the surface oxide, which is the key innovation of this work, clear experimental validation is essential.

Author response to comment 2: We appreciate the reviewers' comments. We investigated the growth mechanism of the InAs/ZnSe QDs through EELS and TEM analyses at both the initial and final stages of growth. The early-stage HAADF-STEM/EELS data provide both compositional and crystallographic evidence for vertex-localized shell nucleation. Zn-rich domains are observed near the InAs core vertices, and the lattice fringes within these domains are non-collinear with those of the adjacent core. This directly demonstrates that the initial ZnSe-rich domains are crystallographically misoriented relative to the InAs core rather than forming through conformal continuation of the core lattice. We have revised the text to clarify:

Description on page 5 of manuscript:

To understand the origin of this phenomenon, we performed TEM with EELS measurements on the core/shells at the initial stage of the reaction (Supplementary Fig. 1). Zn-rich domains were preferentially localized near the vertices of the InAs cores. In the corresponding high-resolution images, the lattice fringes within these Zn-rich regions were non-collinear with those of the adjacent InAs core, demonstrating that the initial domains were crystallographically misoriented rather than conformally extending the core lattice. These observations support a vertex-initiated island-nucleation pathway. The occurrence of approximately four to five wing-like domains in the fully grown particles is also consistent with the growth of multiple independently nucleated domains.

[… *text omitted* …]

One notable observation, consistent with previous studies on similar architectures[26] and our TEM data (Fig. 1d and Supplementary Fig. 3), is that the ZnSe shell does not conformally coat the InAs core. Instead, it often grows as an inverted tetrahedron relative to the core, with the core vertices facing the shell's facet centers rather than its vertices. In addition, although the ZnSe-rich domains initially nucleate without lattice matching at the core vertices, the fully grown core/shell particles exhibit epitaxial registry across the InAs/ZnSe interface.

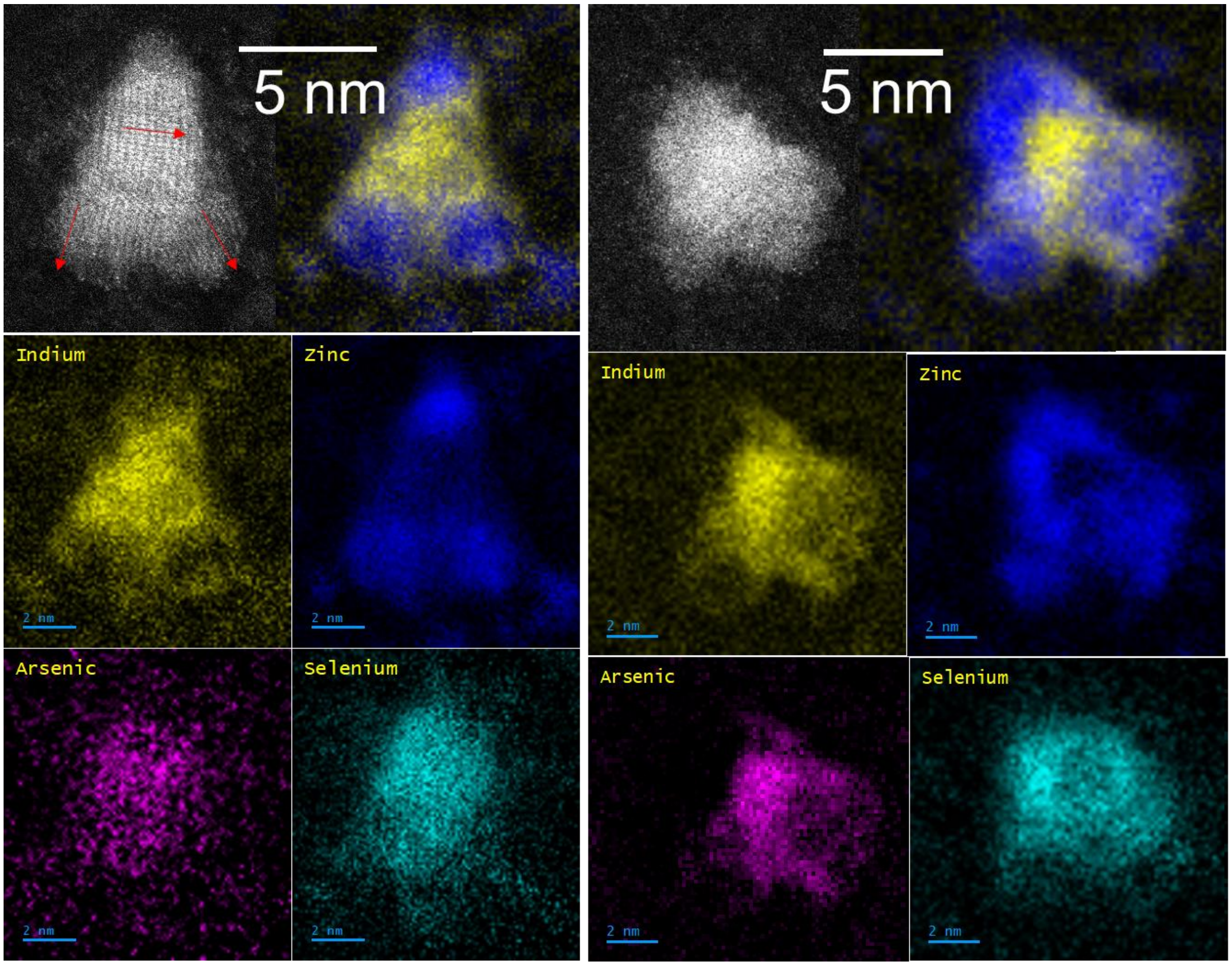

Supplementary Fig. 1. Structural and elemental analysis of InAs/ZnSe core/shell CQDs at the initial stage of shell growth. High-resolution HAADF-STEM images and corresponding EELS elemental maps of two representative particles. Zn-rich domains are preferentially localized near the vertices of the InAs cores, whereas Se is distributed more broadly over the particles. In the high-resolution image, lattice fringes within the vertex-localized Zn-rich domain exhibit an orientation distinct from that of the adjacent InAs core, as indicated by the guide lines. This local lattice misorientation demonstrates that the initial ZnSe-rich domains do not form through a conformal continuation of the InAs core lattice and supports a vertex-initiated island-nucleation pathway.

3. The current oxidation method relies on exposing samples to ambient air for a set duration, which is sensitive to many factors such as purification procedures, storage conditions, and humidity. Establishing a standardized and reproducible oxidation protocol would greatly improve the reliability of the results.

Author response to comment 3: We appreciate the reviewer's valid concern regarding the sensitivity of ambient oxidation to environmental factors. To minimize such variables, all

purification procedures were strictly conducted inside a glove box using anhydrous solvents, and the QDs were oxidized under maintained humidity conditions (20–23 °C, 30 ± 5% RH, and 12 h exposure).
While we explored alternative methods to oxidize the core in a more controlled manner, we were unfortunately unable to establish a viable chemical protocol that yielded the desired properties. Therefore, to improve the standardization and ensure the reliability of our results, we systematically tracked the relationship between the oxidation time, the degree of oxidation, and the PLQY. Specifically, we correlated the PLQY with the degree of oxidation by analysing the absorption shifts and the oxidation peaks in the XPS measurements across various oxidation durations. We believe this systematic evaluation provides a reliable framework for reproducing our results.

Description on page 6 of manuscript:

To address the challenge of shell growth on these enlarged facets, we explored a surface-engineering strategy involving controlled air exposure. To evaluate the effect of core oxidation on subsequent shell growth, 1400 nm InAs cores were exposed to ambient air at room temperature for 0–144 h after purification. As 3d XPS analysis showed a progressive increase in the relative contribution of oxidized arsenic species with increasing exposure time (Supplementary Fig. 4). The resulting InAs/ZnSe core/shell CQDs exhibited their highest PLQYs of approximately 40–45% when the oxidized arsenic contribution was in the range of approximately 17–26%. Based on these results, an oxidation time of 12 h was selected as the standard condition. Detailed experimental conditions are provided in the Methods section.

4. Although the oxide layer enhances the PLQY, the manuscript does not mention experimental errors. Multiple replicate measurements with error bars should be included to convincingly demonstrate this improvement.

Author response to comment 4: To address the reviewer's concern, we performed shell growth under identical conditions using six independently synthesized batches (n = 6) of cores with comparable absorption profiles to evaluate the batch-to-batch reproducibility of the optimized synthesis. Averaged over these replicates, the standard error and standard deviation were 0.58% and 1.42%, respectively, confirming that our synthesis is highly reproducible. In addition, four repeated PLQY measurements of the same sample, performed at 3 h intervals, showed a relative variation of less than 1%.
Separately, the absolute PLQY values are subject to a systematic instrumental uncertainty arising from the detector quantum efficiency and grating efficiency of the spectrograph, each estimated at approximately 5%. Propagated in quadrature, these give a relative instrumental uncertainty of approximately 7%. Because this instrumental uncertainty is substantially larger than the batch-to-

batch reproducibility (1.42%), we have adopted the more conservative instrumental uncertainty (±7%) as the reported error for PLQY values in the manuscript and Table 1, rather than the smaller reproducibility error. We note, however, that since this instrumental uncertainty is a systematic bias common to all samples measured on the same setup, it does not affect the relative comparison between pristine and oxidized samples measured side by side; the PLQY enhancement observed upon oxidation exceeds this conservative uncertainty bound, supporting the robustness of the comparison.

<u>Description on page 29 of Supplementary Information:</u>

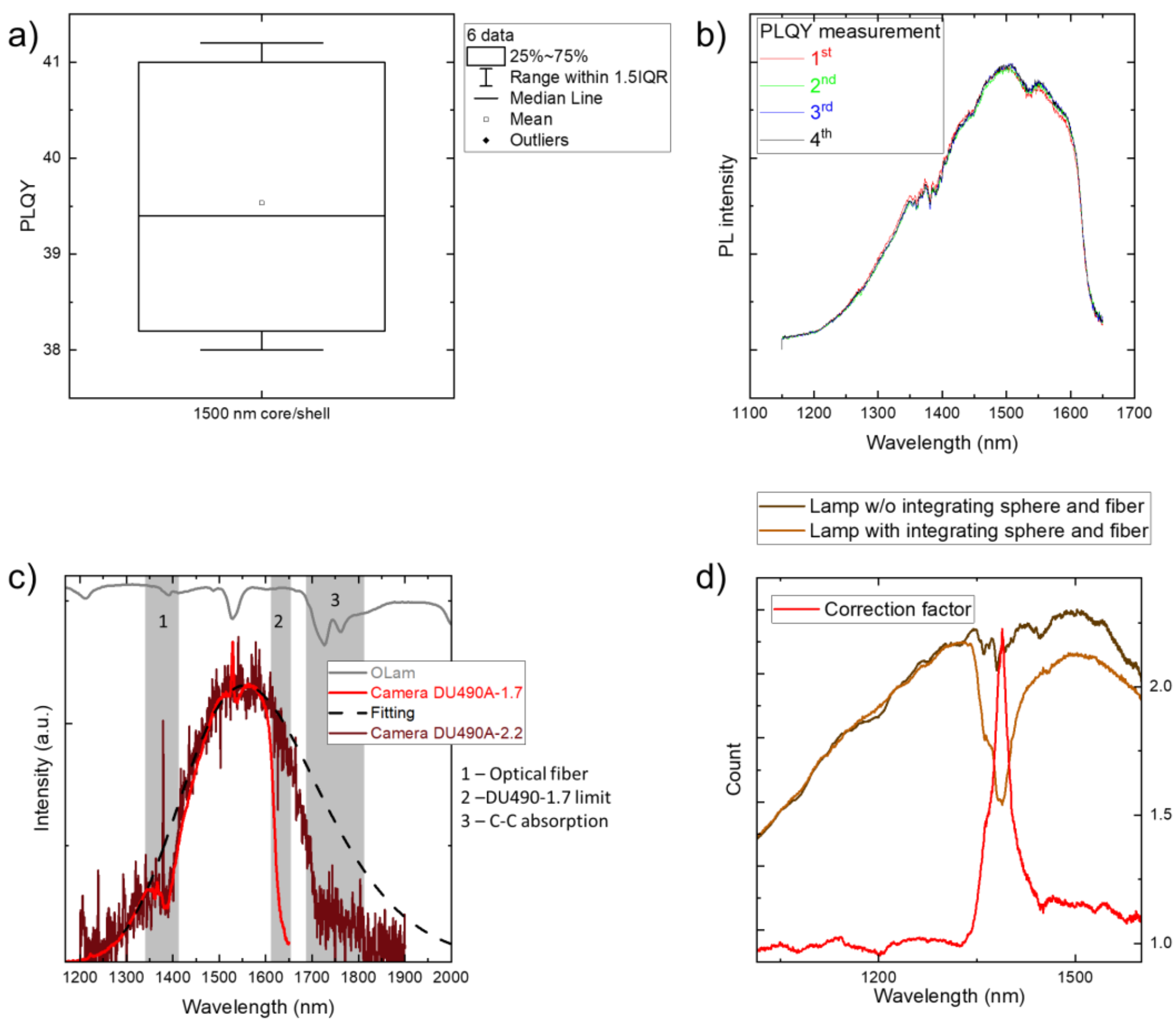

Supplementary Fig. 23 | Reproducibility and validation of the PLQY measurements. a Batch-to-batch distribution of the PLQY obtained from six independently synthesized InAs/ZnSe core/shell CQD batches. The mean PLQY was approximately 39%. **b** Four consecutive PL measurements of the same InAs/ZnSe core/shell CQD sample acquired at 3 h intervals under identical conditions. The relative variation in the measured PLQY was below 1%. **c** Extended-range PL spectrum of the InAs/ZnSe core/shell CQDs measured using a detector covering wavelengths up to 2.2 μm, confirming the absence of substantial additional emission at longer

wavelengths. The shaded regions indicate the spectral ranges affected by detector-response features, and the dashed line represents the fitted emission profile. **d** Broadband-lamp spectra measured using a reference optical path without the attenuation-inducing fiber and using the complete integrating-sphere/fiber configuration employed for the PLQY measurements. The wavelength-dependent correction factor was obtained from the ratio of the two measured spectra and used to correct the solution-phase PL spectra.

Description on page 20 of Methods:

For samples whose emission extended beyond the nominal detector range, the corrected spectrum within the directly measured range was fitted with a single Gaussian function, and only the unmeasured spectral tail was obtained from the fit. Extended-range detector measurements confirmed that no substantial additional emission component was present beyond the fitted range. Measurement repeatability was further confirmed by four consecutive measurements of the same sample at 3 h intervals, with a relative PLQY variation below 1%. Extended-range measurements using a detector covering wavelengths up to 2.2 μm confirmed the absence of substantial additional emission at longer wavelengths. In addition, the wavelength-dependent attenuation introduced by the integrating-sphere/fiber optical path was experimentally determined using broadband-lamp measurements and corrected as described in the Methods. These validation results are summarized in Supplementary Fig. 23. The reported absolute PLQY values carry an estimated relative instrumental uncertainty of approximately 7%, based on the detector quantum-efficiency and grating-efficiency calibrations. Furthermore, we observed that OLAm exhibits slight reabsorption in the infrared region, which decreases the measured PLQY by approximately 3%; however, no sample-specific correction was applied because the residual OLAm concentration could not be quantified accurately for each sample.

5. In Figure 3h, the current density is nearly identical for devices with and without the InAs ETL at the same bias. Given that InAs ETLs typically exhibit modest conductivity and here have a substantial thickness of 40 nm, this observation is kind of surprising and requires an explanation.

Author response to comment 5: To clarify this point, we extracted the carrier mobility from the performed space-charge-limited current (SCLC) measurements (Supporting Fig. 16), which show that the InAs ETL has electron mobility comparable to ZnO and a significantly lower trap density, indicating efficient electron transport despite its 40 nm thickness. Furthermore, as shown in Supporting Fig. 19, the device current is limited by the PTAA hole-transport layer rather than the ETL. Therefore, the similar current density observed in Figure 3h is expected and indicates that the InAs ETL does not limit charge transport. We have updated the details and Supporting Fig. 15 accordingly.

Description on page 19 of Supporting information:

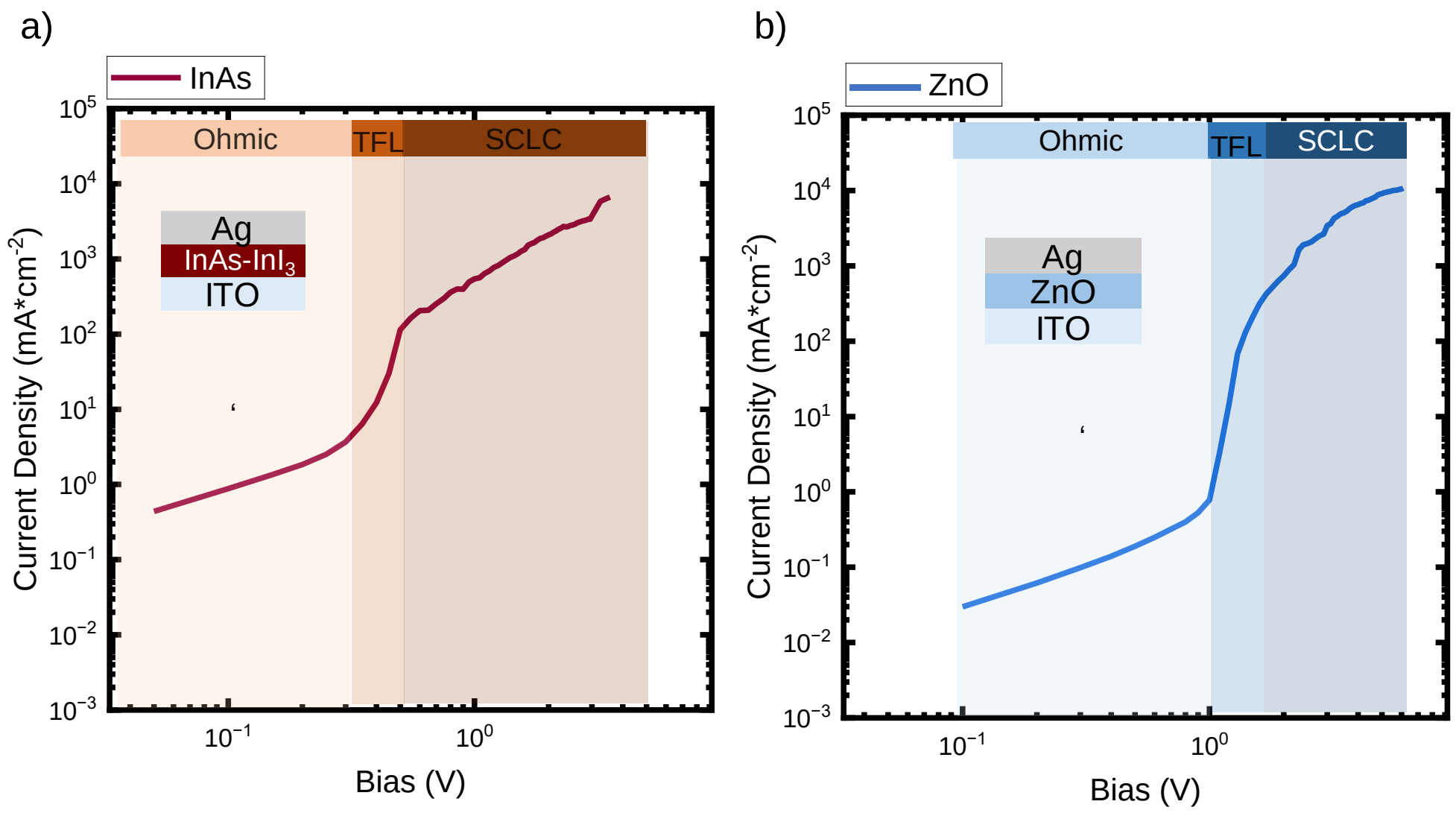

Supplementary Fig 15 SCLC ZnO vs InAs The extracted electron mobility of the InAs layer is $1.3 \cdot 10^{-3}$ cm^2 V^{-1} s^{-1}, which is only modestly lower than that of the ZnO reference ($7.6 \cdot 10^{-3}$ cm^2 V^{-1} s^{-1}). [… *text omitted* …]. Using this approach, the InAs-InI_3 device (260 nm, V_{TFL}= 0.3 V) gave a trap density calculated to be $7.4{\times}10^{15}cm^{-3}$ while the ZnO NPs device (260 nm, V_{TFL}=0.95 V) exhibited a higher trap density of $1.4{\times}10^{16}$ cm^{-3}.

6. In Figure 4a, doping PTAA with merely 5% BCF increases the current density by over two orders of magnitude at 6 V. Since the overall device conductivity is likely limited by the poorly conductive QD emissive layer and the InAs ETL, such a dramatic enhancement solely from PTAA doping is unexpected and warrants a detailed explanation.

Author response to comment 6: We thank the reviewer for this insightful comment. We agree that the device's overall conductivity is determined by the combined contributions of all functional layers. However, our experimental results indicate that the PTAA constitutes a significant component of the overall series resistance. As shown in Supporting Fig. 19, the device current exhibits a strong dependence on the PTAA thickness, demonstrating that charge transport through the HTL substantially influences the device conductivity. In addition, we have included new I-V measurements of pristine and BCF-doped PTAA films (Supporting Fig. 20), which directly show a pronounced increase in HTL conductivity upon BCF doping. We have added this discussion to the Supporting Information.

Description on page 24 of Supporting Information:

Supplementary Figure 20. [… *text omitted* …]. **(d) Lateral current–voltage (I–V) characteristics of pristine PTAA and PTAA-BCF** films measured between two Au electrodes separated by a 10 μm gap. Both films exhibit linear and symmetric I–V behaviour, indicating ohmic charge transport. Both pristine PTAA and PTAA-BCF films exhibit linear and symmetric I-V characteristics over the measured voltage range, indicating ohmic charge transport. Linear fitting yields I-V slopes of $2.8 \cdot 10^{-12}$ and $9.6 \cdot 10^{-9}$ $A \cdot V^{-1}$ for pristine PTAA and PTAA-BCF, respectively. Using the device geometry (L = 10 μm, W = 0.8 cm, t = 30 nm), the corresponding conductivities were calculated to be $1.17 \cdot 10^{-9}$ S cm^{-1} for pristine PTAA and $4 \cdot 10^{-6}$ S cm^{-1} for PTAA-BCF, corresponding to an enhancement of approximately three orders of magnitude upon BCF doping.

7. Please provide the T50 lifetime at defined current density and radiance instead of T66. Besides, independent verification of the reported EQE values would strengthen the claims.

Author response to comment 7: We have revised the manuscript to report the T50 lifetime instead of T66. The revised measurements were performed on a newly fabricated batch of devices under different ambient conditions (24 °C, 60% RH) compared with the original measurements (22 °C, 30% RH). Despite the differences in the absolute T50 values, the relative improvement achieved by BCF-doped PTAA remains unchanged, demonstrating that the enhanced operational stability is reproducible across independently fabricated devices and different testing conditions. This conclusion is further supported by the accelerated ageing experiments presented in the Supporting Information, which show that BCF-doped PTAA consistently exhibits improved resistance to moisture-induced degradation compared with pristine PTAA.
Regarding the second point of the question and in the absence of any accredited laboratory to provide certification for LEDs, we have expanded the Supporting Information to provide a detailed description of the EQE measurement procedure together with an independent validation of the radiometric calibration by using a commercially available LED with a formal datasheet. Radiance measurements were independently verified using a NIST-traceable Ge photodetector (Newport 818-IR) equipped with a calibrated DB15 module. As a reference source, we used a commercial 1550 nm LED (SMB1N-1550S – red curve) with a specified optical output power. The emitted power was measured under three independent geometries: at 5 cm (light-grey dots) and 10 cm (dark-grey dots) through a pinhole (3.8 mm^2), with the total emitted power obtained by accounting for the LED emission profile, and at 11.7 cm (black dots) using a lens tube to optically couple the LED to the photodetector. In all cases, the extracted optical powers were in excellent agreement with the manufacturer's specifications, confirming the accuracy and reliability of our radiometric calibration and, consequently, the reported EQE values.

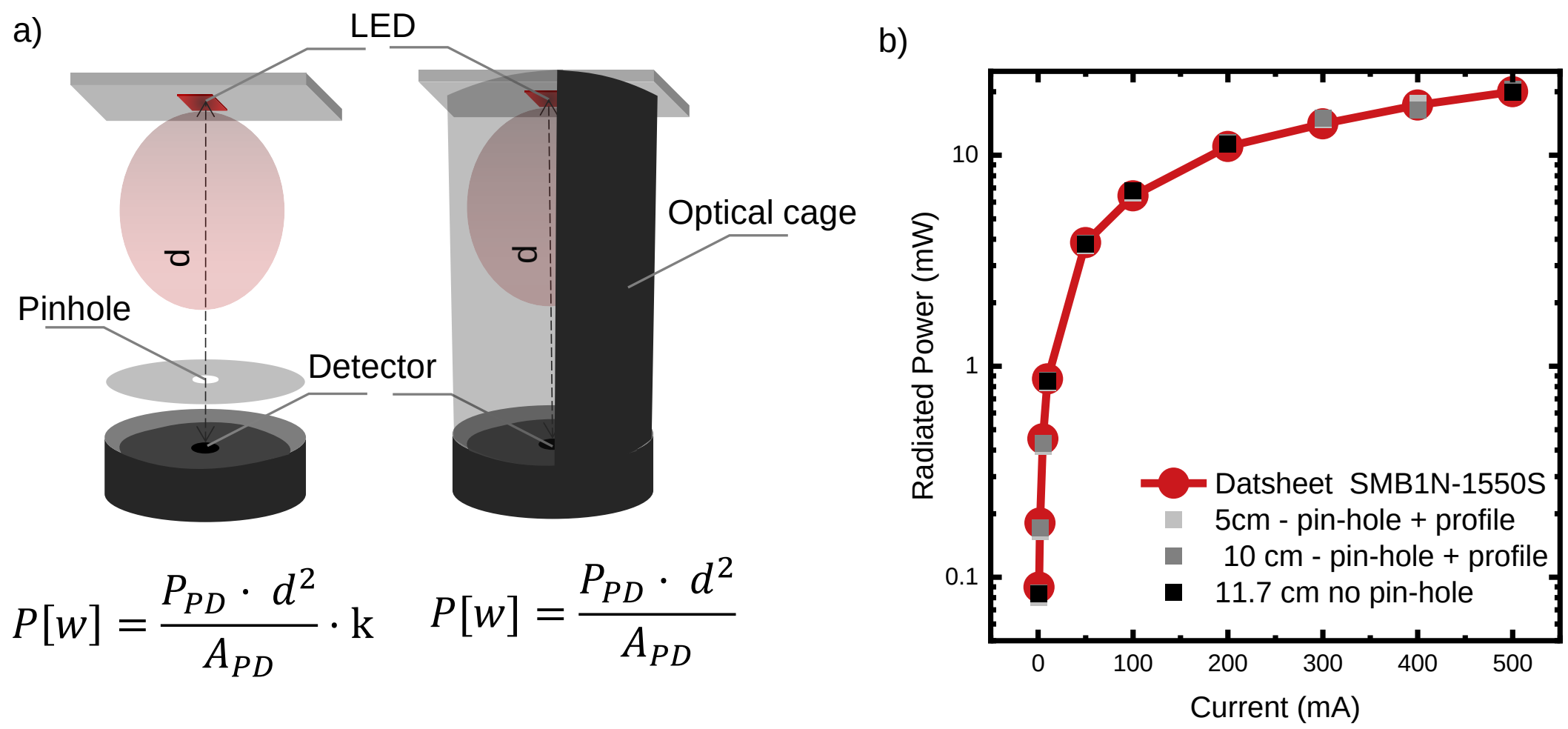

$$P[w] = \frac{P_{PD} \cdot d^2}{A_{PD}} \cdot \mathrm{k} \qquad P[w] = \frac{P_{PD} \cdot d^2}{A_{PD}}$$

k : non-Lambertian profile correction (from datasheet)

Response Figure 1. Validation of the emitted power measurement. a Schematic of the experimental setups used to determine the emitted power of a commercial LED. Measurements were performed with the LED positioned at two different distances (5 and 10 cm) from the detector. The emitted power was determined either by integrating the LED emission profile using the correction factor derived from the specified emission half-angle (67°, k=3.62) (**left**), or by directly collecting the emitted light using an optical cage to suppress ambient light (**right**). **b** Comparison of the emitted power obtained using both measurement approaches with the value reported by the manufacturer.

Caption on page 32 of manuscript:

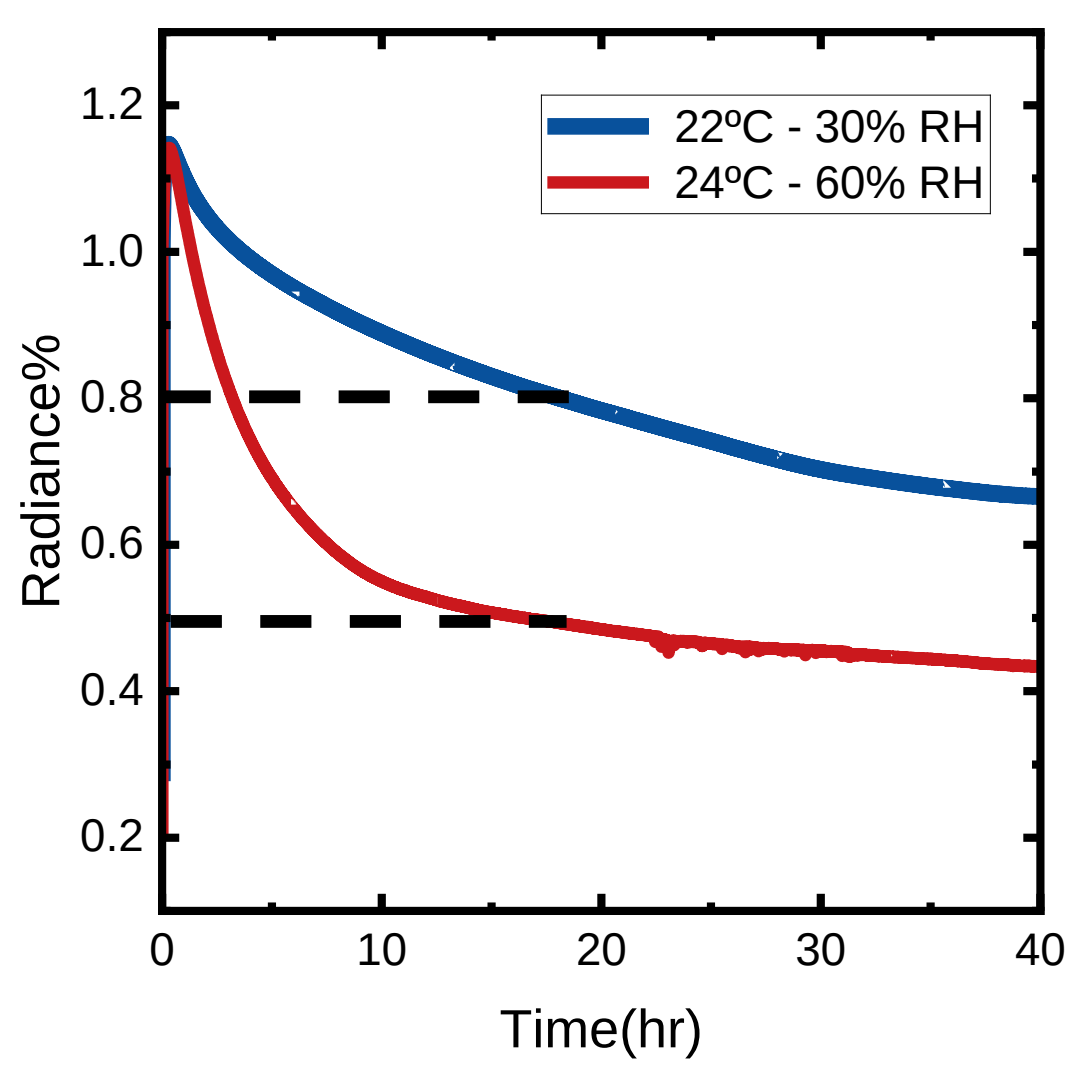

d Stability test on ITO//ZnO//InAs ETL//InAs/ZnSe//PTAA-BCF 5% //MoOx//Au at a constant current density of 100 mA cm^{-2}, under low (blue curve, 22ºC, 30% RH) and high humidity environments (red curve, 24ºC, 60% RH).

Description on pages 13-14 of manuscript:

Finally, the operational stability of the optimized devices was evaluated under constant-current driving in air, without encapsulation. Measurements were performed under a constant current density of 100 mA cm^{-2} (initial bias ~3 V and radiance ~4.4 W sr^{-1} m^{-2}) under two ambient conditions (22 °C/30% RH and 24 °C/60% RH). Under low humidity (30% RH), the device reached 80% of its initial radiance (T_{80}) after 18 h and remained above ~60% of its initial radiance after 40 h. In contrast, at elevated humidity (60% RH), degradation accelerated markedly: the device reached T_{80} in only 3 h and half of the starting radiance (T_{50}) in 16 h, with the radiance decreasing to ~40% of its initial value within the first ~15-20 h before stabilizing.

Description on pages 18-19 of Method:

The external quantum efficiency (EQE) and radiance were determined following the method described in reported papers.[1,46] Assuming Lambertian emission, the total optical flux emitted into the forward hemisphere is given by

$$F_{ext} = \pi \cdot L_0$$

where L_0 is the radiance normal to the emitting surface. The radiance was determined from the optical power measured by the photodetector according to

$$L_0 = \frac{P_{det} \cdot d^2}{S_{det}}$$

where P_{det} is the detected optical power, S_{det} is the active area of the photodetector, and d is the distance between the device and the detector. The EQE was then calculated as

$$EQE = \frac{\pi \cdot P_{det} \cdot d^2 \cdot \lambda \cdot e}{S_{det} \cdot h \cdot c \cdot I}$$

where λ is the EL peak wavelength, I is the device current, e is the elementary charge, h is Planck's constant, and c is the speed of light. The radiance was calculated as

$$R = \frac{P_{det} \cdot d^2}{S_{det} \cdot S_{LED}}$$

where S_{LED} is the emitting pixel area.

8. On page 11, the improved operational stability is attributed to reduced moisture uptake and dopant migration, but these arguments are drawn from perovskite device literature. Direct evidence specific to this QD-LED system should be provided to explain the enhanced lifetime.

Author response to comment 8:

To provide direct evidence specific to our QD-LED system, we have included additional experiments in the Supporting Information (Supporting Fig. 22). Accelerated humidity ageing test show that both the PL intensity and device EQE degrade significantly less in BCF-doped

PTAA (55% and 33%, respectively) than in undoped PTAA (85% and 75%, respectively) under identical conditions. These results directly demonstrate the improved environmental robustness of the doped hole transport layer and provide experimental support for the enhanced operational stability. The manuscript and Supporting Information have been updated accordingly.

Description on page 26 of Supporting Information:

Supplementary Figure 22. Accelerated moisture-aging test of PTAA and PTAA-BCF. a Photograph and schematic of the custom-built environmental chamber used for accelerated humidity aging (25 °C, 80% RH, 3 h). Devices were fabricated using InAs/ZnSe CQDs with 12 h oxidation, a 3-4 ML ZnSe shell, a PL peak at 1330 nm, and a solution PLQY of 33%. **b** Normalized PL spectra of InAs/ZnSe QDs/PTAA films before and after humidity aging, normalized to the maximum PL intensity of the pristine sample. **c** J-V-R characteristics of PTAA-based LEDs before and after humidity aging. **d** EQE of PTAA before and after humidity aging (3.1% before and 0.8% after aging). **e,** Normalized PL spectra of InAs/ZnSe CQDs/PTA-:BCF films before and after humidity aging, normalized to the maximum PL intensity of the pristine sample. **f** J-V-R characteristics of PTAA-BCFLEDs before and after humidity aging, and **g,** the EQE before and after humidity aging (1.7% before and 1.1% after aging).

==

Reviewer 2 Comments to the Author

==

The manuscript describes an air oxidation strategy for improving SWIR emission from InAs/ZnSe QDs. The authors propose that oxidation of the InAs core forms an amorphous interfacial oxide layer that relieves strain during ZnSe shell growth, leading to higher PLQY beyond 1400 nm, and they further apply these emitters in QLEDs near 1550 nm. The PLQY of the QDs and related device performance is nice. However, the mechanistic discussion of how the oxide layer improves the PLQY remains rather preliminary and several key results and data analyses are not sufficiently convincing. The manuscript does not meet the high standard expected for publication in Nature Communications.

Author response: We thank the reviewer for the careful assessment of our manuscript and for acknowledging the PLQY and device performance of the InAs/ZnSe CQDs. We recognize the reviewer's concern that the mechanistic discussion remained preliminary and that several key results and analyses were not sufficiently convincing. Accordingly, we have revised the manuscript extensively to provide stronger experimental support for the proposed role of the amorphous interfacial oxide layer. We added new control experiments and supplementary analyses, refined the interpretation of the structural and spectroscopic results, and clearly distinguished experimentally observed changes from the proposed mechanism. We also strengthened the PLQY validation, reproducibility analysis, and device characterization. We believe that these revisions substantially improve the rigor of the study and address the reviewer's concerns regarding the standard expected for publication in Nature Communications. Detailed responses are provided below.

1. There are several inconsistencies in the manuscript that should be corrected. For example, Supplementary Fig. 2 appears to use an oxidation time of 36 h, whereas the main text refers to oxidation for more than 48 h. In addition, the figure citation in the main text should be carefully checked, since the discussion of the absorption shift appears to refer to Fig. 1b rather than Fig. 1c.

Author response to comment 1: We have extensively revised the manuscript to elucidate the mechanism of core shell growth and the effect of our synthesis method. During this process, we carefully checked the main text and citations. Detailed responses can be found in the comments below.

2. The authors state that the relatively high PLQY of the pristine InAs/ZnSe QDs is due to the continuous injection protocol and precise temperature control. Since the PLQY difference between the pristine and oxidized samples is not very large in some cases, these synthetic parameters should be investigated more carefully.

Author response to comment 2: We demonstrated through high resolution TEM and EELS analyses that the continuous injection process and $ZnCl_2$ degassing conditions determine the dispersibility and morphology of the QDs, as well as the uniformity of the shell coverage on the surface. Specifically, while the synthesis following the conventional method exhibited an efficiency of approximately 7% with strong aggregation, the continuous injection process prevented such aggregation, achieving 26%. Furthermore, the formation of an alloy through temperature control during $ZnCl_2$ degassing ultimately improved the efficiency to 42%. To reflect this, the manuscript has been revised as follows:

Description on page 4 of manuscript:

To investigate this structural degradation at the interface, we first analyzed the InAs/ZnSe core/shell structures grown via conventional heating-up methods. InAs CQDs were synthesized using In(I)Cl and amino-As precursors, following a modified protocol from reported method.[12]
[… *text omitted* …]
Hypothesizing that this aggregation was caused by an excessive amount of shell precursors, we conducted the heating-up process with only half the initial amount of TOP-Se, followed by the continuous injection of the remaining half. [… *text omitted* …]
The degassing of $ZnCl_2$ was initially performed at 90 °C because $ZnCl_2$ can etch the surface of InAs CQDs. However, we found that deliberately inducing this etching process results in structural modifications. [… *text omitted* …] As shown in Fig. 1e, while the initially aggregated core/shells and the pinwheel-shaped shells exhibited PLQYs of only 7% and 26%, respectively, the core/shells synthesized under the optimum conditions achieved an efficiency of approximately 42%.

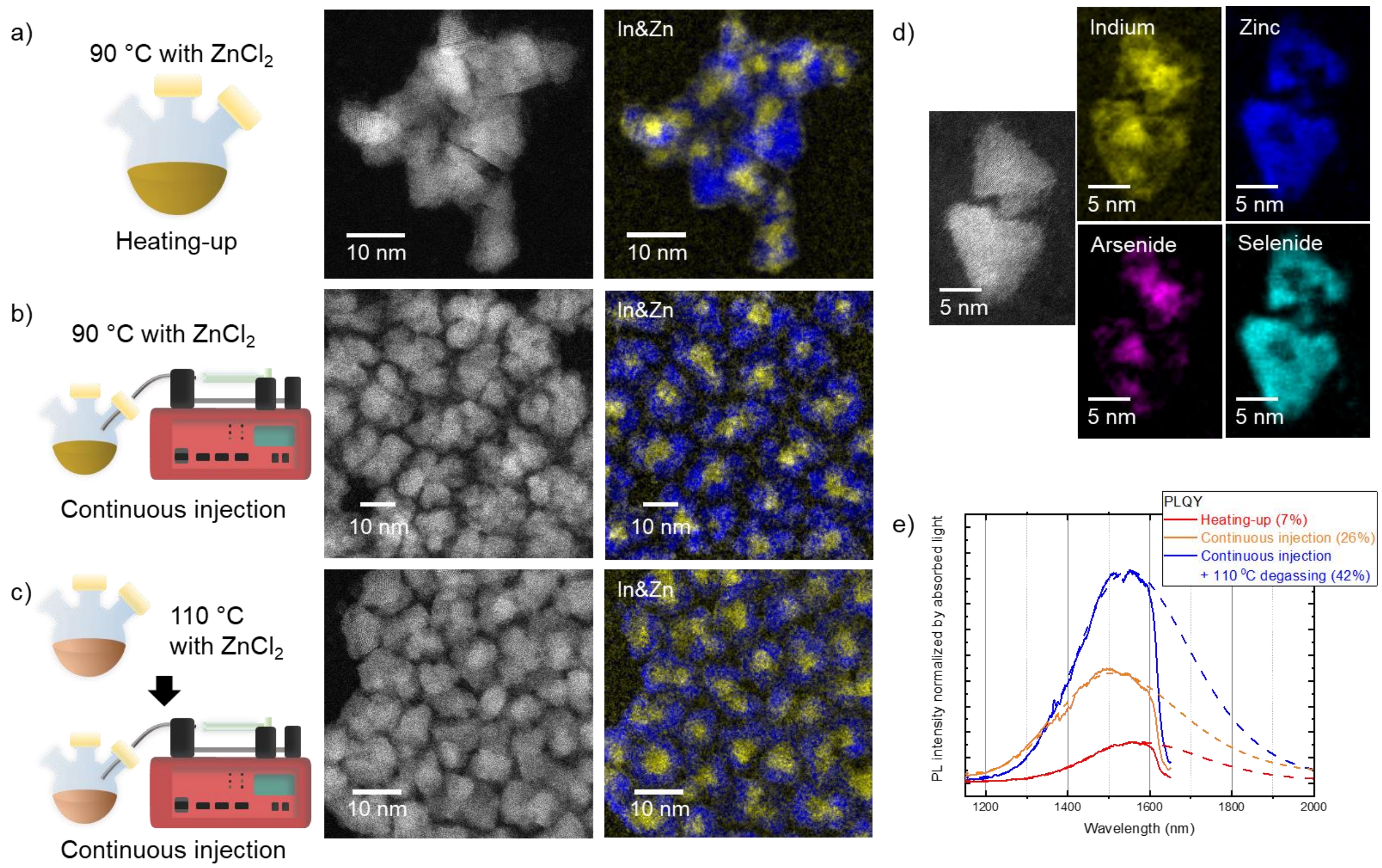

Fig. 1: Elucidation of the InAs/ZnSe core/shell growth mechanism. a HAADF-STEM image and corresponding EELS elemental map (In, yellow; Zn, blue) of InAs/ZnSe core/shell CQDs synthesized via a conventional heating-up method with $ZnCl_2$ degassed at 90 °C, showing severe aggregation. **b** HAADF-STEM image and EELS elemental map of core/shell CQDs synthesized under identical $ZnCl_2$ degassing conditions but with a continuous precursor injection protocol, revealing a pinwheel-like shell morphology with non-conformal ZnSe growth. **c** HAADF-STEM image and EELS elemental map of core/shell CQDs synthesized with $ZnCl_2$ degassed at 110 °C followed by continuous injection, yielding uniform tetrahedral or near-spherical morphologies. **d** EELS elemental maps of a representative CQD from the optimized synthesis (c), showing the spatial distribution of indium, zinc, arsenic, and selenium, with trace co-distribution of In and As in the shell region indicative of interfacial alloy formation. **e** PL spectra of InAs/ZnSe core/shell CQDs synthesized under the three conditions, with PLQY values of 7%, 26%, and 42% for heating-up, continuous injection, and 110 °C degassing with continuous injection, respectively.

3. The PL spectra in Fig. 1d and 1e appear to be strongly affected by solvent absorption in the SWIR region. The manuscript does not clearly explain whether the PLQY measurements were influenced by this absorption, or whether fitted PL spectra were used for PLQY calculation. This raises concerns about the reliability of the reported PLQY values. The authors should optimize the PL measurements, provide proper solvent correction or control experiments, and present

more reliable PL spectra. The current spectral shapes are not convincing for a main figure in Nature Communications.

Author response to comment 3: We thank the reviewer for raising this important concern. To determine whether the recurring spectral feature near 1400 nm originated from the sample or the measurement system, we measured the same broadband lamp through a reference fiber path and through the complete integrating-sphere/fiber optical path used for the solution-phase PLQY measurements under otherwise identical acquisition conditions. The attenuation feature near 1400 nm appeared only when the complete PLQY optical path was used, showing that it originated from the wavelength-dependent response of this optical path rather than from the CQD sample or solvent absorption. We therefore calculated the correction factor as the ratio of the two experimentally measured spectra, $I_{ref}(\lambda)/I_{full}(\lambda)$, and applied it to the solution-phase PL spectra. PLQY was calculated by integrating the spectrally corrected experimental emission within the directly measurable wavelength range. A Gaussian fit was used only to estimate the unmeasured fraction of the emission extending beyond the nominal detector limit and was not used to replace the measured spectrum. Within the measurable range, the corrected experimental spectra closely agree with the fitted profiles. Measurements using an extended-range detector further confirmed that no substantial additional emission component was present beyond this range.

<u>Description on page 20 of Methods:</u>

Absolute PLQY was measured using an integrating sphere following the method of de Mello et al.[48] To correct the wavelength-dependent attenuation introduced by the PLQY optical path, the same broadband lamp was measured through (i) a reference fiber path that did not produce the 1400 nm attenuation feature (I_{ref}) and (ii) the complete integrating-sphere/fiber configuration used for the PLQY measurements (I_{full}), under otherwise identical acquisition conditions. The correction factor was calculated as $I_{ref}(\lambda)/I_{full}(\lambda)$ and applied to the solution-phase PL spectra. For samples whose emission extended beyond the nominal detector range, the corrected spectrum within the directly measured range was fitted with a single Gaussian function, and only the unmeasured spectral tail was obtained from the fit. Extended-range detector measurements confirmed that no substantial additional emission component was present beyond the fitted range. The reported absolute PLQY values carry an estimated relative instrumental uncertainty of approximately 7%, based on the detector quantum-efficiency and grating-efficiency calibrations. Furthermore, we observed that OLAm exhibits slight reabsorption in the infrared region, which decreases the measured PLQY by approximately 3%; however, no sample-specific correction was applied because the residual OLAm concentration could not be quantified accurately for each sample.

4. The electron microscopy and elemental mapping results in Fig. 2a and 2b are difficult to interpret. In particular, in Fig. 2b oxygen appears to be distributed across the whole particle, whereas Zn seems to be located mainly near the upper surface. These maps do not clearly support the proposed interfacial oxide model. The authors should also explain in much more detail how the XPS data in Fig. 2c support their conclusion about Zn coordination, rather than relying mainly on literature comparison. In addition, Se elemental mapping is missing from Fig. 2, which makes the discussion of the ZnSe shell incomplete.

Author response to comment 4: To address this concern, we have acquired new high resolution TEM and EELS images that provide a much clearer visualization of the elemental distribution. Furthermore, to discuss how the In and Zn peaks shift due to oxidation and the newly identified alloy formation between the InAs core and the ZnSe shell, we measured and compared the XPS spectra of samples with varying shell thicknesses. Based on this thickness dependent analysis, we have provided a more detailed explanation of the Zn and In coordination rather than relying solely on literature comparisons. Additionally, we have included the elemental mapping results for As and Se alongside those for In and Zn to complete the structural discussion. To reflect this, the manuscript has been revised as follows:

Description on page 7 of manuscript:

To investigate the role of interfacial oxidation, we synthesized core/shell CQDs with a thin shell layer and conducted EELS compositional analysis. Both the pristine and oxidized-core samples exhibited high initial PLQYs of 44% and 42%, respectively (Supplementary Figs. 5a and 5b). As shown in Fig. 2c, the EELS mapping of the pristine InAs/ZnSe CQDs reveals a distinct indium signal but lacks any localized oxygen signal in the CQDs, as shown in Supplementary Fig. 6. In contrast, the EELS mapping of indium and oxygen for the oxidized-core/shell CQDs in Fig. 2d reveals a localized enhancement of the oxygen signal at positions coinciding with the In-rich region, which is absent in the pristine sample (Supplementary Fig. 7).

Description on page 8 of manuscript:

To further investigate these chemical species, XPS analysis was conducted on samples with different shell thicknesses and on the pinwheel-shaped sample shown in Fig. 1b (Supplementary Fig. 9). In both the thick-shell and pinwheel-shaped samples, the In 3d signal was largely attenuated, whereas the Zn 2p spectra were dominated by a ZnSe-like doublet, with the Zn $2p_{3/2}$ component near 1021 eV. Under thin shell conditions, however, both the In 3d and Zn 2p spectra exhibited similar trends. First, tracking the interfacial alloy formation was challenging because the reported binding energies of potential alloy species (such as In_2Se_3 and Zn_3As_2) overlap closely with the conventional InAs and ZnSe peaks.[30,31] Instead, the distinct changes emerged in the higher binding energy regions, suggesting the presence of different chemical species.

Specifically, in the pristine sample, additional peaks became apparent near 445.6 and 453.2 eV for In, and 1023.26 and 1046.5 eV for Zn, which are presumed to originate from metal chlorides such as $InCl_3$ and $ZnCl_2$.[32,33] In contrast, for the oxidized sample, these secondary peaks appeared with increased relative intensity at slightly lower binding energies (near 445.2 and 452.9 eV for In 3d and 1022.9 and 1045.9 eV for Zn 2p), which suggests the additional presence of metal oxides such as In_xO_y and Zn_xO_y. The prominent spectral contributions from surface ligands and oxidized chemical species in the thin shell samples can be attributed to the increased surface-to-volume ratio, different synthetic conditions, and the limited probing depth of XPS (see Methods and Supplementary Fig. 9 for details). By contrast, the pinwheel-shaped particles consisted of spatially separated InAs cores and ZnSe wing domains, with no clear evidence of additional Zn-related chemical species.

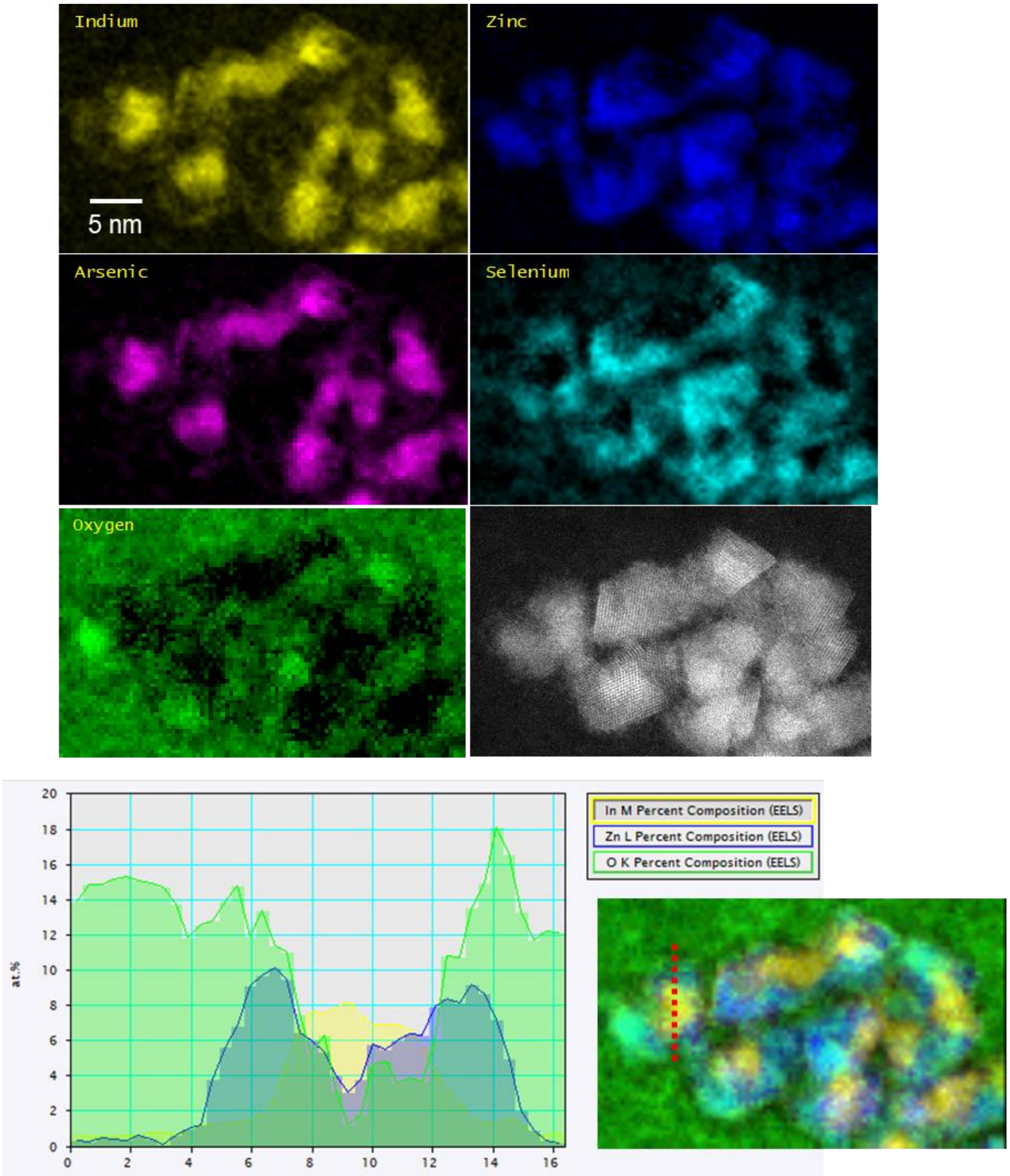

Supplementary Fig. 6. Elemental distribution and line-profile analysis of pristine InAs/ZnSe core/shell CQDs. HAADF-STEM image and corresponding EELS elemental maps of indium, zinc, arsenic, selenium, and oxygen for a cluster of pristine InAs/ZnSe core/shell CQDs. Bottom left, line profile of In, Zn, and O atomic percentage along the dashed line indicated in the overlay image (bottom right). Although a background oxygen signal is present throughout the sample due to methanol treatment during TEM preparation, the oxygen profile does not show localized co-enhancement with indium, in contrast to the oxidized sample (Supplementary Fig. 8).

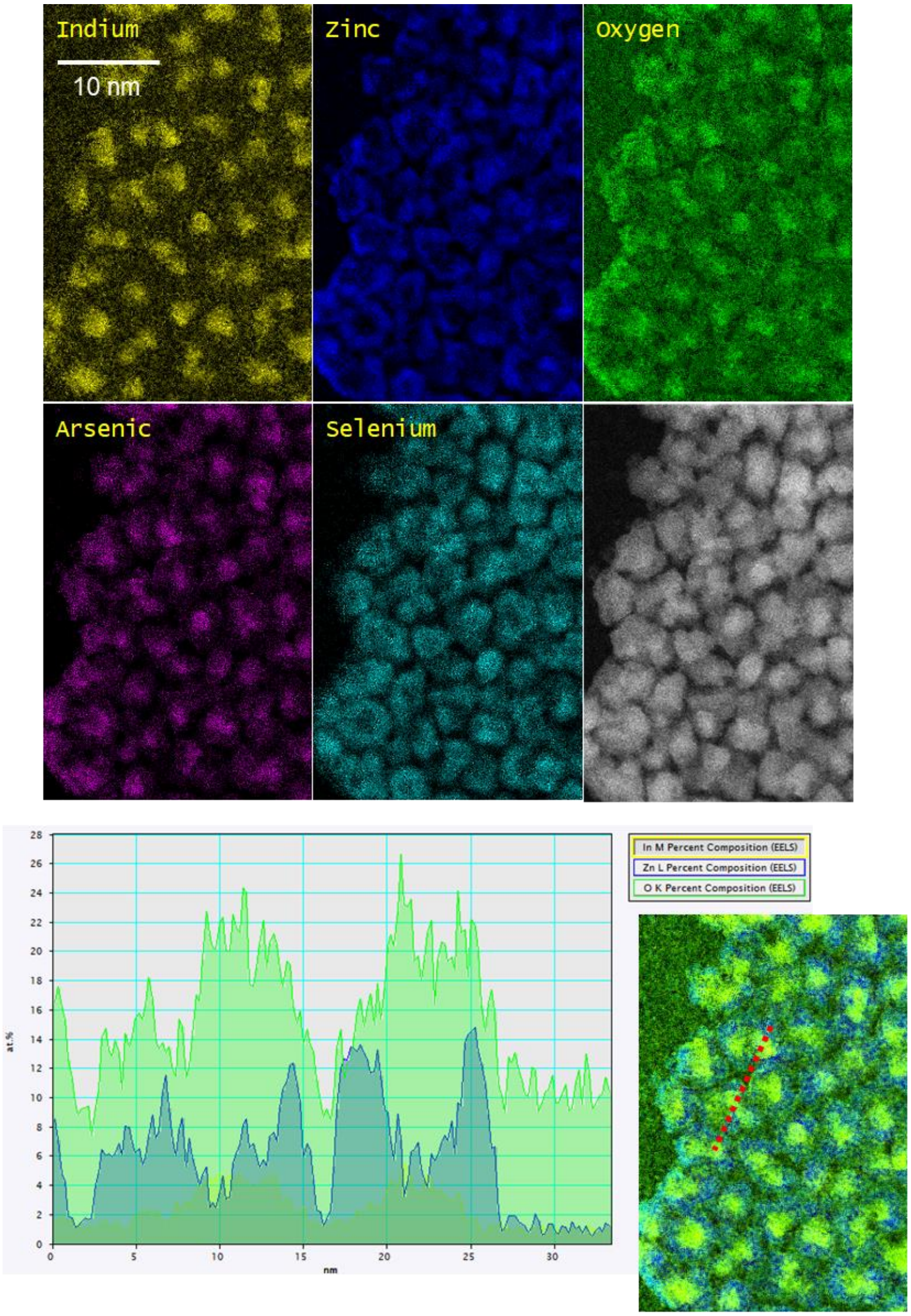

Supplementary Fig. 7. Elemental distribution and line-profile analysis of oxidized InAs/ZnSe core/shell CQDs. HAADF-STEM image and corresponding EELS elemental maps of indium, zinc, arsenic, selenium, and oxygen for a cluster of oxidized InAs/ZnSe core/shell CQDs. Bottom left, line profile of In, Zn, and O atomic percentage along the dashed line indicated in the overlay image (bottom right), showing that the oxygen signal is enhanced at positions coinciding with indium, in contrast to the pristine sample (Supplementary Fig. 7).

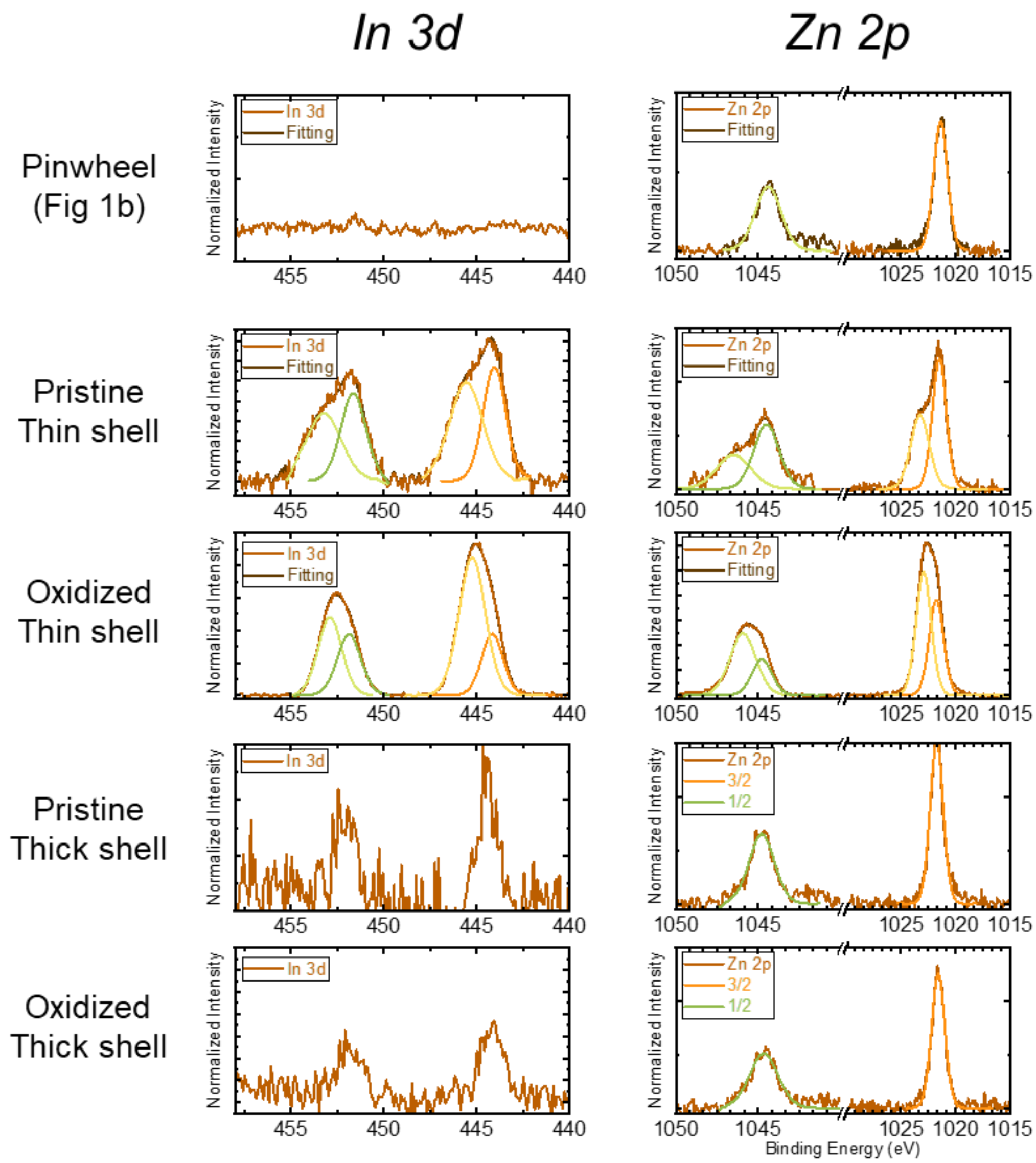

Supplementary Fig. 9. XPS spectra of In 3d and Zn 2p regions for pinwheel, pristine, and oxidized InAs/ZnSe core/shell CQDs with thin and thick shells. Left, In 3d spectra; **right**, Zn 2p spectra, with fitted components shown where resolvable. In the thick-shell samples and pinwheel one, the In 3d signal is largely obscured by noise due to the limited XPS probing depth.

5. The statement that "the interfacial oxide layer suppressed interfacial alloying while maintaining a similar shell thickness" requires a clearer explanation and stronger evidence. The elemental maps in Supplementary Fig. 12 are also confusing. For example, In appears to be distributed throughout the whole particle, and As seems to appear near the particle surface. These observations should be carefully explained.

Author response to comment 5: We thank the reviewer for this observation, which is indeed consistent with our revised understanding of the growth mechanism. The co-distribution of In and As within the shell region is now attributed to interfacial alloy formation: $ZnCl_2$ etches the InAs core surface, and the resulting In and As species re-participate in shell growth rather than remaining confined to the core. This is consistent with the reviewer's observation that In and As are not sharply confined to the core in our elemental maps.
The statement referred to by the reviewer has been revised accordingly. Rather than suggesting that the interfacial oxide suppresses alloy formation altogether, we now interpret the structural evolution as follows. The co-distribution of In and As within the shell region indicates that species etched from the InAs core re-participate in shell growth, leading to an In/As-containing interfacial alloy. At the same time, the oxidized thin-shell sample shows XRD peaks closer to the bulk ZnSe position than the pristine thin-shell sample, indicating that the shell reaches a more bulk-like ZnSe lattice structure at an earlier stage of growth. We have therefore revised the manuscript to distinguish the observed interfacial alloy formation from the more bulk-like structural evolution of the oxidized shell. We have also added Se elemental maps alongside In, Zn, and As (Fig. 1 and Supplementary Figs. 1–3) to provide a more complete structural picture.
Regarding the claim of comparable shell thickness, we based this on two independent measures. First, TEM analysis showed no significant difference in overall particle size between the pristine and oxidized samples. Second, we compared the ratio of the absorption intensity in the region above 800 nm, which originates solely from the InAs core, to that below 600 nm, which contains contributions from both the core and the ZnSe shell. This ratio was essentially identical between the two samples, indicating that the relative volume fraction of the shell - and therefore the shell thickness - is comparable.

Description on page 8 of manuscript:

To verify whether these effects originated from variations in the shell thickness, TEM images and absorption spectra were compared. Given the absence of significant size differences in the TEM images and the identical intensity ratios between the core-originating peaks (above 800 nm) and both core- and shell- originated peaks (below 600 nm), the core and shell thicknesses are considered to be comparable between the samples.

6. The Raman data in Fig. 2e show only a very small shift, around 1 cm^{-1}, between the two samples. It is unclear whether such a small difference is sufficient to support the proposed strain relaxation mechanism. Moreover, the XRD peaks of the InAs/ZnSe samples appear to shift to higher angles. The authors should clarify whether ZnSe shell growth is epitaxial in both samples and whether the InAs core is compressed in either case.

Author response to comment 6: We thank the reviewer for this important observation. To address this concern, we performed Lorentzian fitting on the InAs LO phonon region (separated from the TO band near 220 cm^{-1}) for both pristine and oxidized core/shell CQDs across all shell thicknesses examined, and re-examined the spectra in terms of both peak position and lineshape rather than relying on the peak position alone.

This analysis revealed that the two samples differ not primarily in their absolute peak positions, but in how the LO band evolves with shell thickness. In the pristine CQDs, the LO band progressively shifts toward higher wavenumbers and broadens as the shell thickness increases from 1–2 to 7 monolayers, consistent with increasing compressive-strain signatures in the InAs cores. In contrast, the oxidized core/shell CQDs retain a largely unchanged LO-band position and lineshape across the examined shell thicknesses, consistent with the interfacial oxide limiting additional shell-thickness-dependent strain accumulation.

We believe this thickness-dependent contrast, rather than a single-point shift, provides a more robust signature of strain accumulation, and is consistent with the divergence in PLQY observed between the two systems upon thick-shell growth (40% vs. 23%). We have revised the manuscript to present the data and discussion in these terms, and have included the full Lorentzian fitting parameters (peak position and FWHM for all conditions) in Supplementary Figure 10 and Supplementary Table 1.

In the XRD data, the shift to higher angles originates from the ZnSe shell rather than the InAs core. The oxidized thin-shell sample shows diffraction peaks closer to the bulk ZnSe position than the pristine thin-shell sample, indicating that the shell reaches a more bulk-like ZnSe lattice structure at an earlier stage of growth.

Regarding epitaxy, the new microscopy results show that the initially nucleated ZnSe-rich domains are crystallographically misoriented relative to the InAs core, whereas the fully grown core/shell particles exhibit interfacial lattice registry. The detailed HAADF-STEM/EELS analysis and corresponding images are provided in our response to Comment 7 below.

<u>Description on page 9 of manuscript:</u>

To investigate the mechanical strain exerted on the InAs core, Raman spectroscopy was performed on pristine and oxidized core/shell CQDs with varying shell thicknesses. In the pristine CQDs, the InAs LO phonon band progressively shifts toward higher wavenumbers and broadens as the shell thickness increases from 1–2 to 7 monolayers (Supplementary Fig. 10), consistent with the accumulation of compressive strain in the core.[34] In contrast, the oxidized core/shell CQDs exhibit a LO phonon band that remains largely unchanged in both position and lineshape across all shell thicknesses examined, suggesting that the interfacial oxide region limits the additional accumulation of compressive strain in the core as the ZnSe shell thickens. This thickness-independent behavior in the oxidized samples, contrasting with the pronounced spectral evolution in pristine CQDs, is consistent with the divergence in PLQY observed

between the two systems upon thick-shell growth (40% vs. 23%) and with a strain-buffering role of the interfacial oxide during shell thickening.

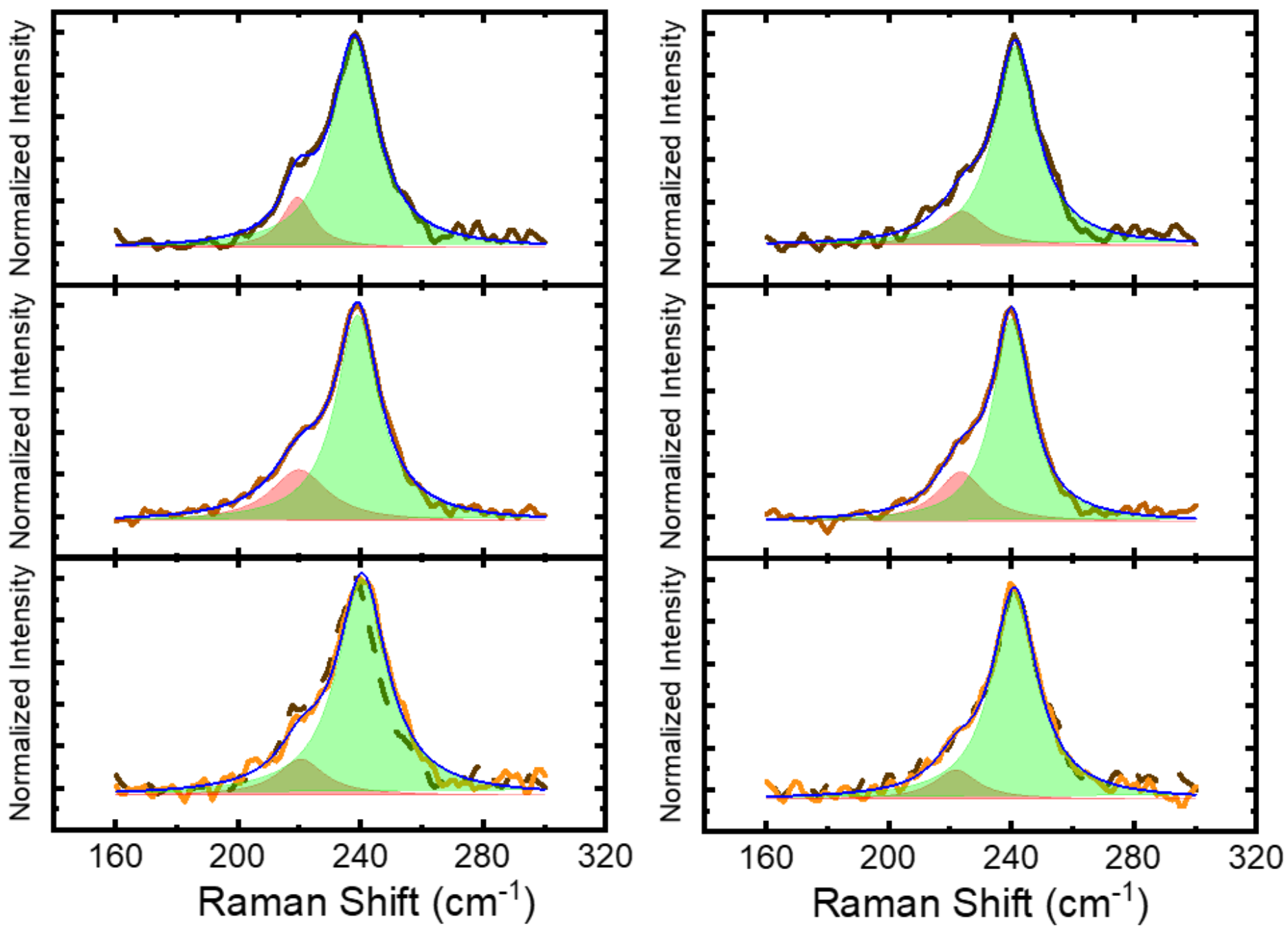

Supplementary Fig. 10. Two-component Lorentzian fitting of the InAs LO phonon mode for pristine and oxidized InAs/ZnSe core/shell CQDs. Raman spectra of pristine (left) and oxidized (right) core/shell CQDs with 1–2 ML (top), 4 ML (middle), and 7 ML (bottom) shell thickness, fit with two Lorentzian components (green, main LO component; pink, secondary component near 220 cm^{-1}) and their sum (blue). In the 7 ML panels, the corresponding 1–2 ML spectrum and fit (dashed) are overlaid for direct comparison, highlighting the pronounced shift and broadening of the LO band in the pristine sample relative to the comparatively unchanged lineshape in the oxidized sample. Extracted peak positions and FWHM values are summarized in Supplementary Table 1.

7. The proposed growth model in Fig. 2f remains speculative. The authors should provide high resolution TEM, STEM, or other structural characterization to support the proposed inverse oriented growth model and the claimed interfacial structure.

Author response to comment 7: We thank the reviewer for this important comment. To strengthen the experimental basis of the proposed growth pathway, we acquired new high-resolution HAADF-STEM and EELS data at both the initial and final stages of shell growth. At the initial stage, Zn-rich domains are preferentially localized near the vertices of the InAs cores, and their lattice fringes are non-collinear with those of the adjacent core. These observations

directly support vertex-initiated nucleation of crystallographically misoriented ZnSe-rich domains. In fully grown particles, continuous lattice fringes are observed across the InAs/ZnSe interface, indicating the development of interfacial registry during subsequent growth.

Based on these results, we have revised the manuscript to distinguish the directly observed initial nucleation and final interfacial registry from the intermediate structural evolution, which remains a proposed growth pathway. The inverse-oriented schematic is therefore presented as a model consistent with the observed initial and final structures rather than as a directly resolved sequence of intermediate states.

<u>Description of Supplementary Information:</u>

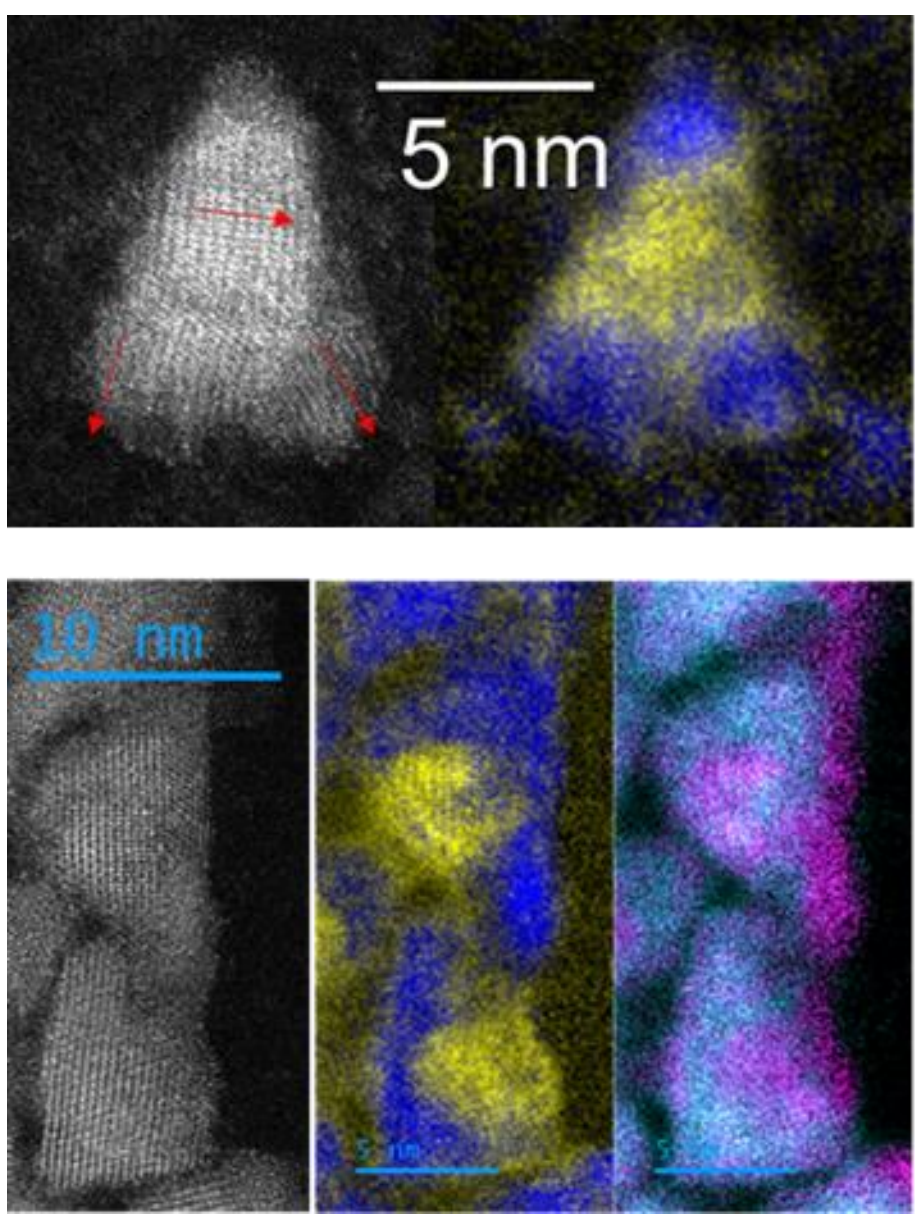

Response Figure 2. Representative HAADF-STEM images and corresponding EELS elemental maps showing (a) vertex-localized, crystallographically misoriented ZnSe-rich domains at the initial stage of shell growth and (b) continuous lattice fringes across the InAs/ZnSe interface in fully grown particles. The panels are reproduced from Supplementary Figs. 1 and 3.

8. In the LED section, the authors should clearly state the size of the InAs/ZnSe QDs used in the devices and the corresponding ZnSe shell thickness.

Author response to comment 8: We have added the size of the InAs/ZnSe quantum dots and the corresponding ZnSe shell thickness to the LED section of the manuscript to improve clarity.

<u>Description on page 10 of manuscript:</u>

Leveraging the tunable optical properties of our InAs/ZnSe CQDs, we demonstrated LEDs spanning a broad spectral range from 1300 to 1550 nm (Fig. 3a). Device optimization was performed using the largest-core InAs/ZnSe CQDs, which exhibit a first excitonic absorption peak at 1400 nm and a film PL emission centered at 1500 nm, resulting in electroluminescence centered at 1525 nm (Supplementary Fig. 13). These devices employ CQDs coated with a 3-4 ML ZnSe shell (lateral size of ~11 nm) and subjected to controlled surface oxidation for 12 h, yielding a relatively high PLQY of ~40%.

9. The device discussion should be updated in light of recent studies on water treated ZnMgO electron transport layers and positive aging in QLEDs. These works suggest that the role of oxide electron transport layers, electron injection, and interfacial aging may be more complex than the conventional picture of excessive electron injection. The pronounced positive aging behavior observed in Fig. 4d should also be discussed in this context.

Author response to comment 9: We thank the reviewer for highlighting these recent studies. We agree that recent reports have considerably expanded the understanding of positive aging in QLEDs beyond the conventional interpretation based solely on excessive electron injection. In particular, studies on ZnO- and ZnMgO-based ETLs suggest that positive aging arises from the dynamic evolution of oxide layers and their interfaces through both moisture-assisted and electrically induced processes. We have revised the manuscript to discuss our observations in the context of these recent findings. While our devices exhibit a qualitatively similar transient increase in radiance, the available evidence has largely been established for conventional p-i-n QLEDs. In contrast, our devices employ an inverted (n-i-p) architecture in which an InAs interlayer is introduced between the ZnO ETL and the emissive layer, creating two distinct interfaces (ZnO/InAs and InAs/emissive layer) whose evolution under electrical stress has not yet been systematically investigated. Therefore, although our observations are consistent with an interfacial origin of positive aging, the microscopic mechanisms governing both the initial performance enhancement and the subsequent degradation cannot be unambiguously assigned in the present architecture.

Description on page 14 of manuscript:

[… *text omitted* …] Under both humidity conditions, the devices exhibit a characteristic transient increase in radiance during the initial stage of operation, followed by gradual degradation. While similar positive-aging behavior has been reported for oxide-based ETLs,[45–48] the additional interfaces introduced by the InAs interlayer make the microscopic origin in the present architecture difficult to identify.

10. The energy level diagram in Fig. 3e should be reorganized. In its current form, the energy alignment and interfacial barriers are not sufficiently clear.

Author response to comment 10: Figure 3e has been revised and reorganized to provide a clearer presentation of the energy level alignment and interfacial barriers, improving the readability of the energy level diagram.

11. In Supplementary Fig. 14, the authors state that oleic acid binds more weakly to the InAs QD surface than oleylamine. This appears to contradict previous reports. The authors should provide direct evidence or appropriate references to support this statement.

Author response to comment 11: We thank the reviewer for this comment. Previous studies have shown that OLAm is not completely displaced from the surface of InAs and other III–V quantum dots during ligand exchange with carboxylate or halide ligands, resulting in residual amine species after the exchange process. However, we agree that our original wording unnecessarily implied a comparison between the binding strengths of OA and OLAm on the InAs QD surface, which is not supported by direct evidence in the present work. Since such a comparison is not essential to our study, we have removed this statement entirely and revised the caption to provide only a procedural description of the InI_3 solid-state ligand exchange process.

Description on page 24 of Supporting Information:

[…] (b) Schematic of the InI_3 solid-state ligand exchange (SSLE). OA-capped InAs QDs are deposited by spin coating, exposed to the InI_3 solution for 30 s, and spin-rinsed with anhydrous methanol followed by toluene, resulting in a compact, conductive QD film while preserving film integrity. […]

12. In Supplementary Fig. 15, the authors should explain why InI3 treatment removes surface oxides from the InAs QDs. The chemical basis for this claim is not clear, and additional characterization would be needed to support it.

Author response to comment 12: We agree that the original wording implied a mechanism that is not directly demonstrated by the data presented. We have therefore revised the text to distinguish between our experimental observations and the proposed interpretation. Specifically, XPS analysis shows that the surface oxide fraction decreases from approximately 23% in the untreated InAs QDs to approximately 8% after InI_3 treatment. As a possible explanation, we refer to the established behavior of indium trihalides reported in the literature. In particular, $InBr_3$ has been shown to act as a Lewis acidic precursor that promotes surface reconstruction through halide-mediated coordination processes, leading to oxide suppression on III-V quantum dots. Given the analogous chemistry of InI_3, albeit with weaker dissociation due to the more covalent In-I bond, we suggest that a similar process may occur in our system.

We have revised the Supporting Information caption to better align with the literature and rephrased the discussion to report only the experimental observation from the XPS analysis, namely the decrease in the relative As-O peak contribution after InI_3 treatment.

Description on page 17 of Supporting Information:
[…] (c-d) High-resolution As 3d XPS spectra of InAs-OA and InAs-InI_3 films. The oxidized arsenic (As–O) component at 44.5 eV is reduced from approximately 23% (c) to 8% (d), consistent with a successful resurfacing. […]

===

Reviewer 3 Comments to the Author

===

Oxidation-Induced Interface Engineering in InAs/ZnSe Core/Shell Quantum Dots for Highly Efficient Shortwave Infrared Emission by Kim et al. This work reports on the first InAs quantum dot-based LED with emission maximum beyond 1400 nm. The performance numbers – in comparison to similar, previously reported devices – are worthy of a publication in Nature Communications. In addition, the authors attempt a derivation of broader lessons learnt for other quantum dot-based LEDs, e.g. the introduction of an oxide layer to reduce lattice strain. While I value this approach (and deem it appropriate for a publication in this journal), this attempt is somewhat hindered by the combination of experimental procedures used in this study that apparently work well all together but obscure to some extent the effect of each individual measure. To be specific, in addition to their main claim that the oxide layer is key to the observed performance, the paper also introduces the insightful utilization of a large-band gap quantum dot buffer layer to modify electron transport and a doping strategy for their hole transport layer to balance charge carrier injection. In fact, I enjoyed reading the sections detailing these two strategies almost more than the oxide layer part as they were well-supported by the experimental data and readily explainable in their outcome. The oxide layer part could be equally interesting but it would require a number of substantial clarifications, which are detailed below. In summary, this work is original, goes beyond the state-of-the-art in a challenging field and has the potential to be of high interest to a broad readership. However, it would require major revisions to achieve full consistency between some of its interpretations and the provided experimental data.

Author response: We thank the reviewer for the thoughtful and balanced assessment of our work, particularly for recognizing its originality, its advance beyond the state of the art, and its potential interest to a broad readership. We also appreciate the reviewer’s concern that the combination of several successful strategies could obscure the contribution of each individual measure, especially the specific role of the oxide-containing interface. In response, we have revised the manuscript to more clearly separate the effects of interface oxidation, the large-bandgap quantum-dot buffer layer, and hole-transport-layer doping. We have added control experiments and expanded the structural and spectroscopic analyses related to the oxide layer, while revising the mechanistic discussion to maintain closer consistency between the interpretations and the experimental data. These changes clarify which conclusions are directly supported by experiment and which represent a proposed mechanistic picture. Detailed responses to the reviewer’s comments are provided below.

1. I am concerned with the determination of the PLQY. I would like to see a calibration of their integrating sphere-based set up with a known standard, confirming that the PLQY values in this regime are accurately determined. First, the authors are surprised themselves that even the pristine quantum dots exhibit record-breakingly high PLQYs. Second, how exactly was the PLQY calculated? From the raw PL data or the fitted spectra (which have much higher area than the raw data)?

Author response to comment 1: We thank the reviewer for raising these important questions regarding both the origin of the relatively high PLQY of the pristine CQDs and the reliability of its absolute determination.

The high PLQY of the pristine CQDs results from the optimized shell-growth procedure rather than from the oxidation treatment. In the conventional heating-up synthesis, the CQDs exhibited substantial aggregation and a PLQY of approximately 7%. Continuous injection of the Se precursor suppressed aggregation and increased the PLQY to approximately 26%. Increasing the $ZnCl_2$ degassing temperature to 110 °C, together with continuous injection, produced more uniformly developed core/shell particles and increased the PLQY to approximately 42%. These structural and optical comparisons are now presented in the revised Fig. 1.

We agree that a certified PLQY standard in the 1400–1600 nm range would provide the most direct validation of the absolute values. However, we were unable to obtain a certified standard or access an independent facility with validated absolute PLQY capability in this spectral range. We therefore performed several complementary validation measurements. An independently prepared PbS CQD sample measured using the same integrating-sphere configuration yielded a corrected PLQY of 23%, consistent with the expected range for comparable PbS CQDs, although we emphasize that it is used as a secondary consistency check rather than as a certified standard.

PLQY was calculated by integrating the spectrally corrected experimental emission within the directly measured range. The wavelength-dependent correction factor was experimentally obtained by measuring the same broadband lamp through a reference optical path and through the complete integrating-sphere/fiber configuration. A Gaussian fit was used only to estimate the small unmeasured spectral tail beyond the detector limit and did not replace the measured spectrum. Extended-range measurements confirmed the absence of a substantial additional emission component.

Finally, six independently synthesized batches exhibited a standard deviation of 1.42%, and four repeated measurements of the same sample showed less than 1% relative variation. The reported PLQY values include the more conservative estimated instrumental uncertainty of ±7%.

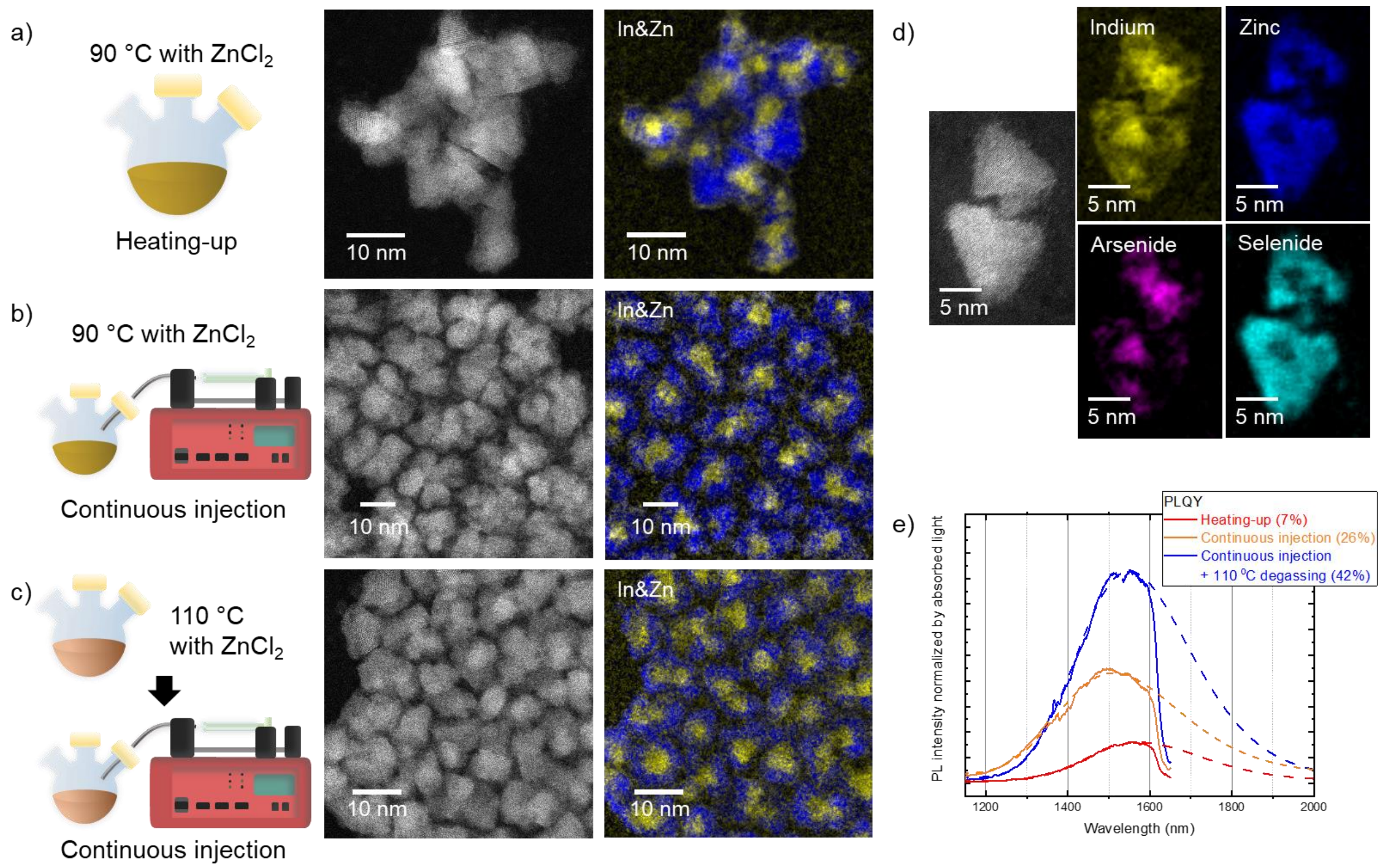

Fig. 1: Elucidation of the InAs/ZnSe core/shell growth mechanism. a HAADF-STEM image and corresponding EELS elemental map (In, yellow; Zn, blue) of InAs/ZnSe core/shell CQDs synthesized via a conventional heating-up method with $ZnCl_2$ degassed at 90 °C, showing severe aggregation. **b** HAADF-STEM image and EELS elemental map of core/shell CQDs synthesized under identical $ZnCl_2$ degassing conditions but with a continuous precursor injection protocol, revealing a pinwheel-like shell morphology with non-conformal ZnSe growth. **c** HAADF-STEM image and EELS elemental map of core/shell CQDs synthesized with $ZnCl_2$ degassed at 110 °C followed by continuous injection, yielding uniform tetrahedral or near-spherical morphologies. **d** EELS elemental maps of a representative CQD from the optimized synthesis (c), showing the spatial distribution of indium, zinc, arsenic, and selenium, with trace co-distribution of In and As in the shell region indicative of interfacial alloy formation. **e** PL spectra of InAs/ZnSe core/shell CQDs synthesized under the three conditions, with PLQY values of 7%, 26%, and 42% for heating-up, continuous injection, and 110 °C degassing with continuous injection, respectively.

<u>Description on page 29 of Supplementary Information:</u>

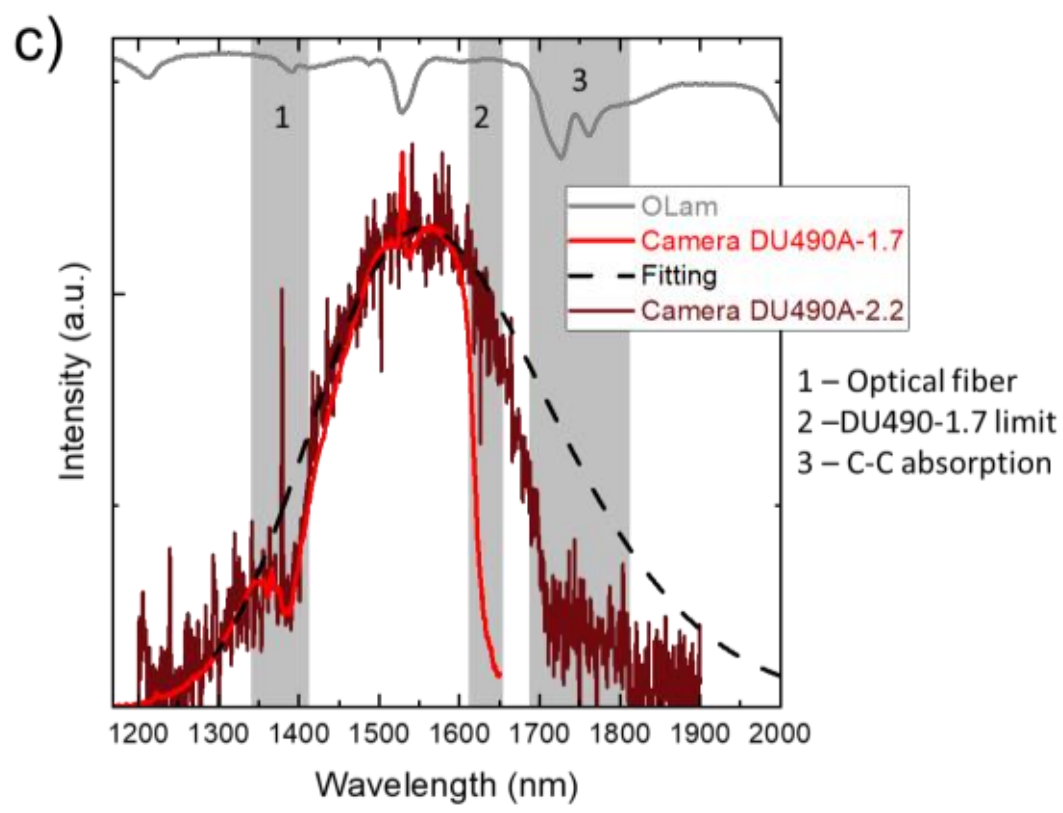

Supplementary Fig. 23 | Reproducibility and validation of the PLQY measurements. [… *text omitted* …] **c** Extended-range PL spectrum of the InAs/ZnSe core/shell CQDs measured using a detector covering wavelengths up to 2.2 μm, confirming the absence of substantial additional emission at longer wavelengths. The shaded regions indicate the spectral ranges affected by detector-response features, and the dashed line represents the fitted emission profile.

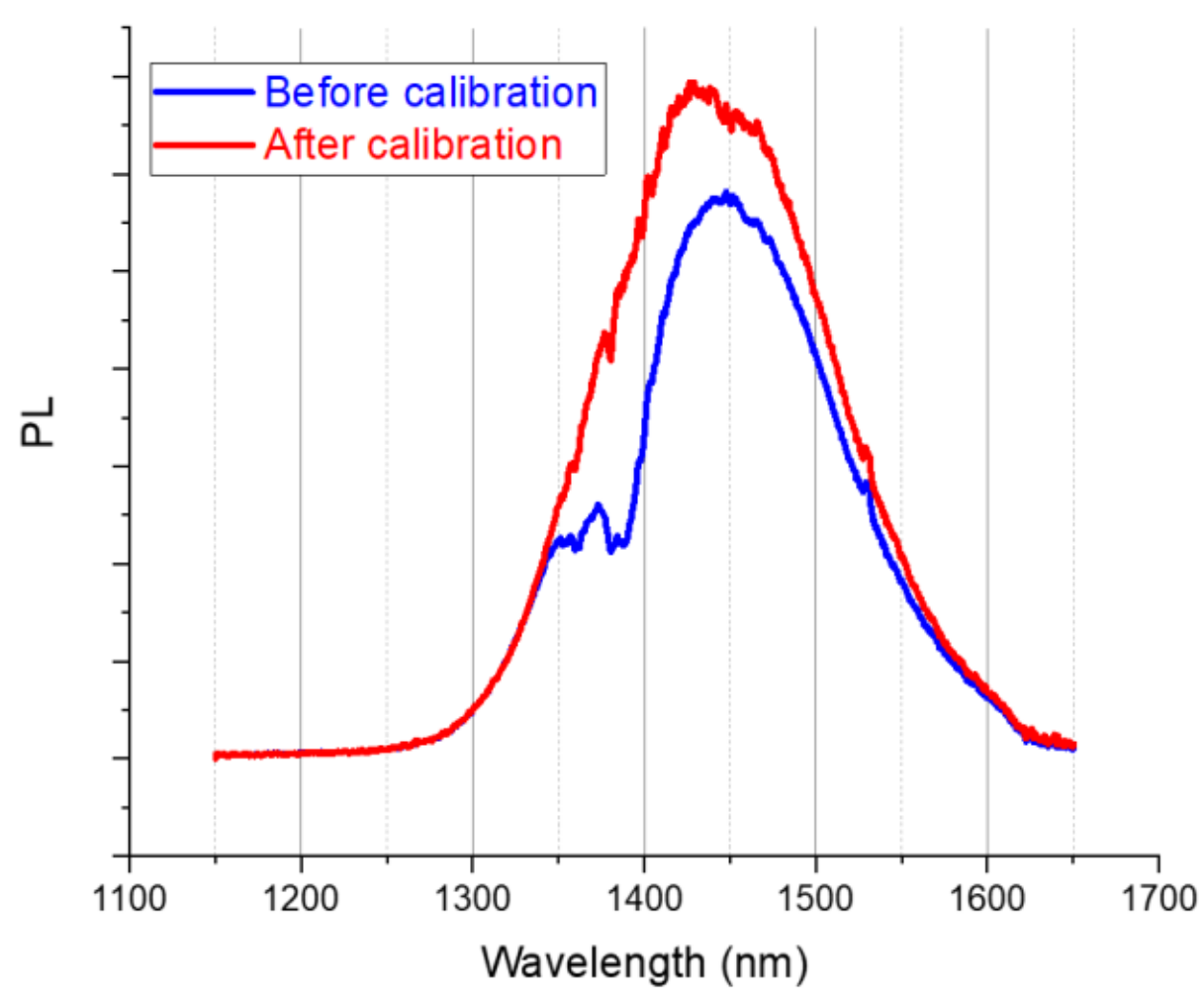

Response Figure 3. PL spectrum of a PbS CQD control before and after optical-path correction. The corrected measurement yielded a PLQY of 23%.

2. To this point: I am concerned by the recurring drop in practically all PL spectra around 1400 nm. When comparing the solution PL spectra to the thin film spectra in Fig. S13, it appears that

this feature only appears in solution. This would suggest an impurity in the solution phase that significantly impacts the recorded PL. If so, how can the PLQY results be reliable if they depend on impurities? I suggest that the authors identify the nature of this impurity, remove it and demonstrate that the PLQY is still the same. Fig. S13 implies that this should be possible.

Author response to comment 2: We thank the reviewer for this careful observation. To determine whether the recurring feature near 1400 nm originated from the CQD solution or from the measurement system, we measured the same broadband lamp through two optical configurations: a reference path that did not include the attenuation-inducing fiber assembly and the complete integrating-sphere/fiber path used for the PLQY measurements. The pronounced attenuation near 1400 nm appeared only in the complete optical path, demonstrating that it was an instrumental response rather than a solution-phase impurity.
We therefore obtained a wavelength-dependent correction factor from the ratio of the two lamp spectra and applied it to all solution-phase PL spectra. Measurements using an extended-range detector confirmed that no substantial additional emission component was present beyond the directly measured range. We also examined possible absorption by OLAm. Its absorption was weak and broad in the SWIR region and was estimated to reduce the measured PLQY by approximately 3%, but it did not reproduce the sharper feature near 1400 nm. Because the residual OLAm concentration could not be quantified for each sample, no sample-specific correction was applied.

Description on page 20 of Methods:

Absolute PLQY was measured using an integrating sphere following the method of de Mello et al.[47] To correct the wavelength-dependent attenuation introduced by the optical path used for the PLQY measurements, the same broadband lamp was measured with and without the additional fiber/optical components under otherwise identical acquisition conditions. The ratio of the two measured spectra was used as a wavelength-dependent correction factor and applied to the solution-phase PL spectra. For samples whose emission extended beyond the nominal detector range, the corrected spectrum within the directly measured range was fitted with a single Gaussian function, and only the unmeasured spectral tail was obtained from the fit. Extended-range detector measurements confirmed that no substantial additional emission component was present beyond the fitted range. The reported absolute PLQY values carry an estimated relative instrumental uncertainty of approximately 7%, based on the detector quantum-efficiency and grating-efficiency calibrations. Furthermore, we observed that OLAm exhibits slight reabsorption in the infrared region, which decreases the measured PLQY by approximately 3%; however, this effect was intentionally excluded from the calibration.

Description on page 29 of Supplementary Information:

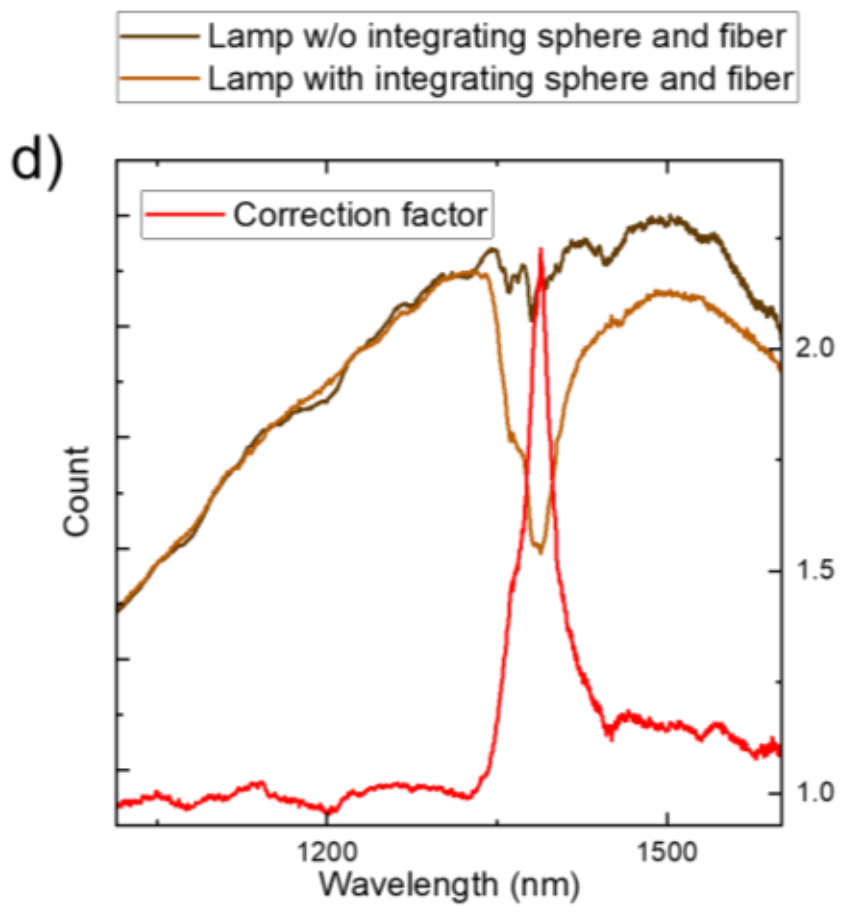

Supplementary Fig. 23 | Reproducibility and validation of the PLQY measurements. [… *text omitted* …] **d** Broadband-lamp spectra measured using a reference optical path without the attenuation-inducing fiber and using the complete integrating-sphere/fiber configuration employed for the PLQY measurements. The wavelength-dependent correction factor was obtained from the ratio of the two measured spectra and used to correct the solution-phase PL spectra.

3. It is puzzling that the authors provide XPS measurements "after ZnCl2 treatment but before shell growth" while the corresponding XRD measurements were taken after shell growth. I suggest streamlining these two experiments into a unified step in their synthesis procedure. Preferentially, this would be after shell growth.

Author response to comment 3: We thank the reviewer for this suggestion. To directly compare the chemical and structural evolution at the same stage of synthesis, we added thickness-dependent XPS analyses of the InAs/ZnSe core/shell samples and evaluated them together with the corresponding post-growth XRD measurements.

For the thin-shell samples, XPS reveals distinct In 3d and Zn 2p chemical environments in the pristine and oxidized samples. In the oxidized sample, the secondary In and Zn components shift toward lower binding energies, consistent with an increased contribution from oxygen-coordinated species. In the thick-shell samples, the In signal is strongly attenuated and the Zn spectrum is dominated by ZnSe-like components, as expected from the limited XPS probing depth. The corresponding XRD data show that the oxidized thin-shell sample exhibits diffraction peaks closer to bulk ZnSe than the pristine thin-shell sample, indicating that the shell reaches a more bulk-like ZnSe structure at an earlier growth stage.

The $ZnCl_2$-treated core measurements have been retained as a separate pre-growth control because they probe the initial surface chemical environment before shell formation. We have

revised the manuscript to clearly separate this pre-growth analysis from the unified post-growth XPS/XRD comparison.

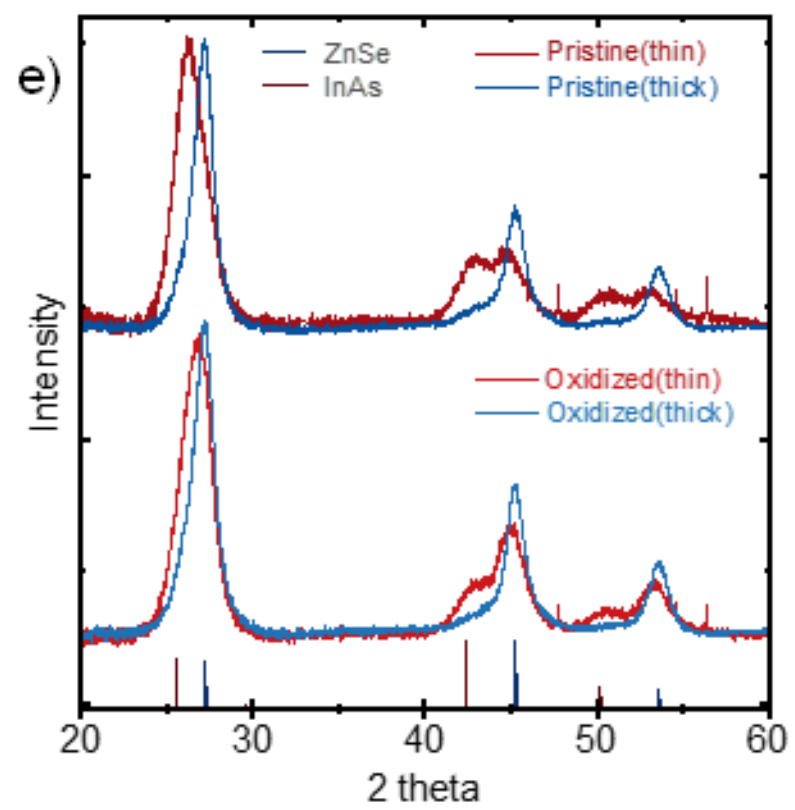

Fig. 2: Optical characterization and structural analysis for oxidation-associated strain evolution in InAs/ZnSe core/shell CQDs. [… *text omitted* …] **e** XRD patterns of thin- and thick-shell InAs/ZnSe CQDs synthesized from pristine and oxidized cores. Reference peak positions for bulk ZnSe (blue) and InAs (red) are indicated.

Description on page 10 of Supplementary Information:

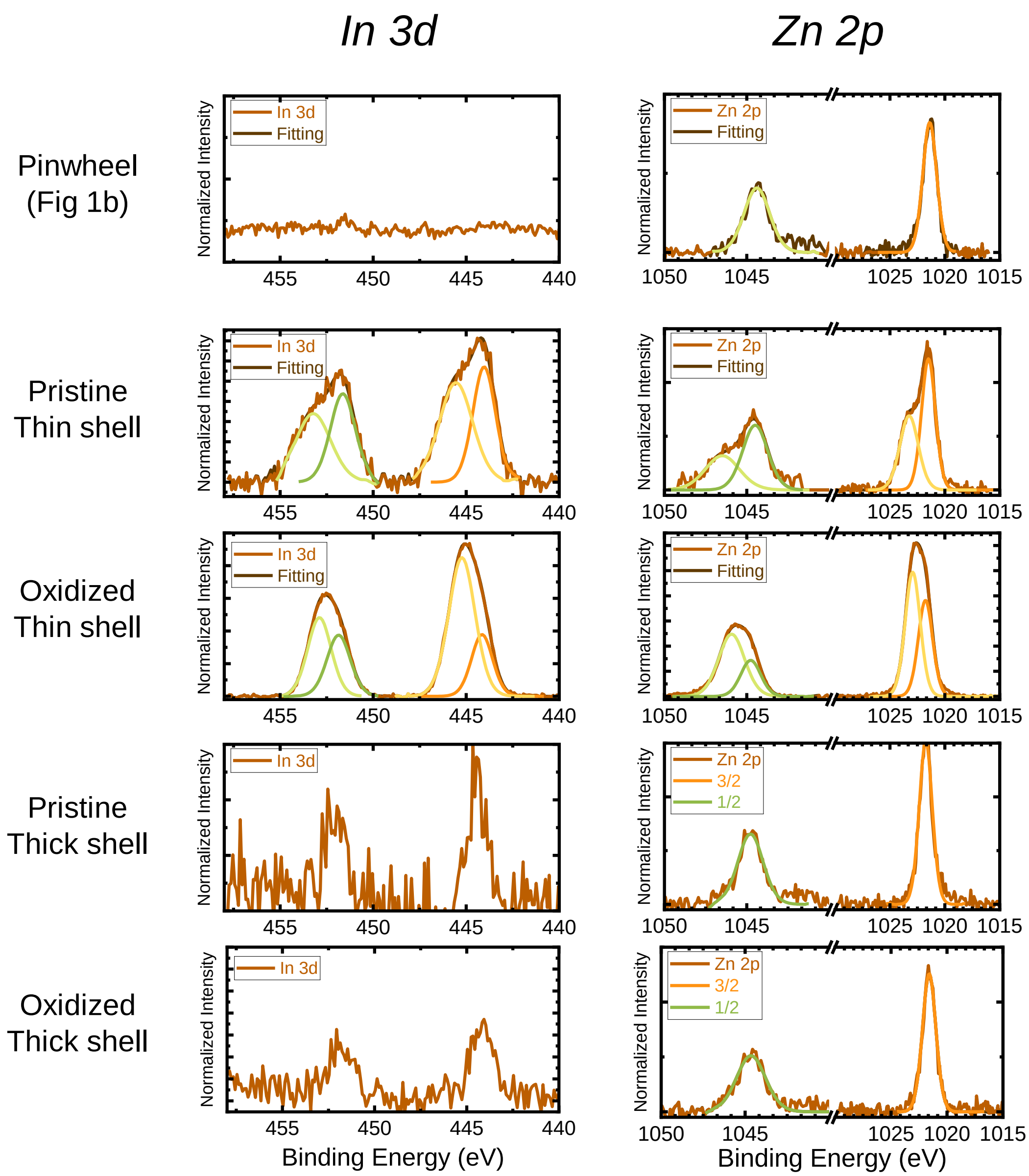

Supplementary Fig. 9. XPS spectra of In 3d and Zn 2p regions for pinwheel, pristine, and oxidized InAs/ZnSe core/shell CQDs with thin and thick shells. Left In 3d spectra; **right** Zn 2p spectra, with fitted components shown where resolvable. In the thick-shell samples and pinwheel one, the In 3d signal is largely obscured by noise due to the limited XPS probing depth.

4. The authors claim that “the ZnSe shell does not conformally coat the InAs core”, and they base this conclusion on Fig. 2a and Fig S12. With all due respect, I cannot come to this conclusion based on that data. The same applies to the sketch in Fig. 2f, which tries to illustrate this view. This inability comes from the fact that the provided high resolution TEM image is simply not very good. The authors have a state-of-the-art probe-corrected JEOL JEM ARM200F NEOARM microscope at their disposal which should easily be capable of delivering the required atomic resolution to support their claim. The contrast in the images in Figure S8 and S10 to me looks promising for retrieving such information from their samples. Without this improved data, the whole paragraph detailing the nucleation of ZnSe islands on InAs is pure speculation.

Author response to comment 4: We thank the reviewer for emphasizing the need for clearer structural evidence. We acquired new high-resolution HAADF-STEM and EELS measurements using the probe-corrected microscope at both the initial and final stages of shell growth. At the initial stage, Zn-rich domains are preferentially localized near the vertices of the InAs cores. The lattice fringes within these domains exhibit orientations distinct from those of the adjacent cores, demonstrating that the initial shell domains do not form by conformal continuation of the InAs lattice.

In the fully-grown pinwheel-like particles, the ZnSe-rich domains remain spatially separated around the InAs core, directly demonstrating incomplete and nonconformal shell coverage under this growth condition. In contrast, particles synthesized under the optimized conditions exhibit a more compact morphology and continuous lattice fringes across the final core/shell interface. We have revised the text to base the nonconformal-growth conclusion on these direct observations and to describe the intermediate evolution toward the inverse-oriented final structure as a proposed mechanism rather than a directly imaged sequence.

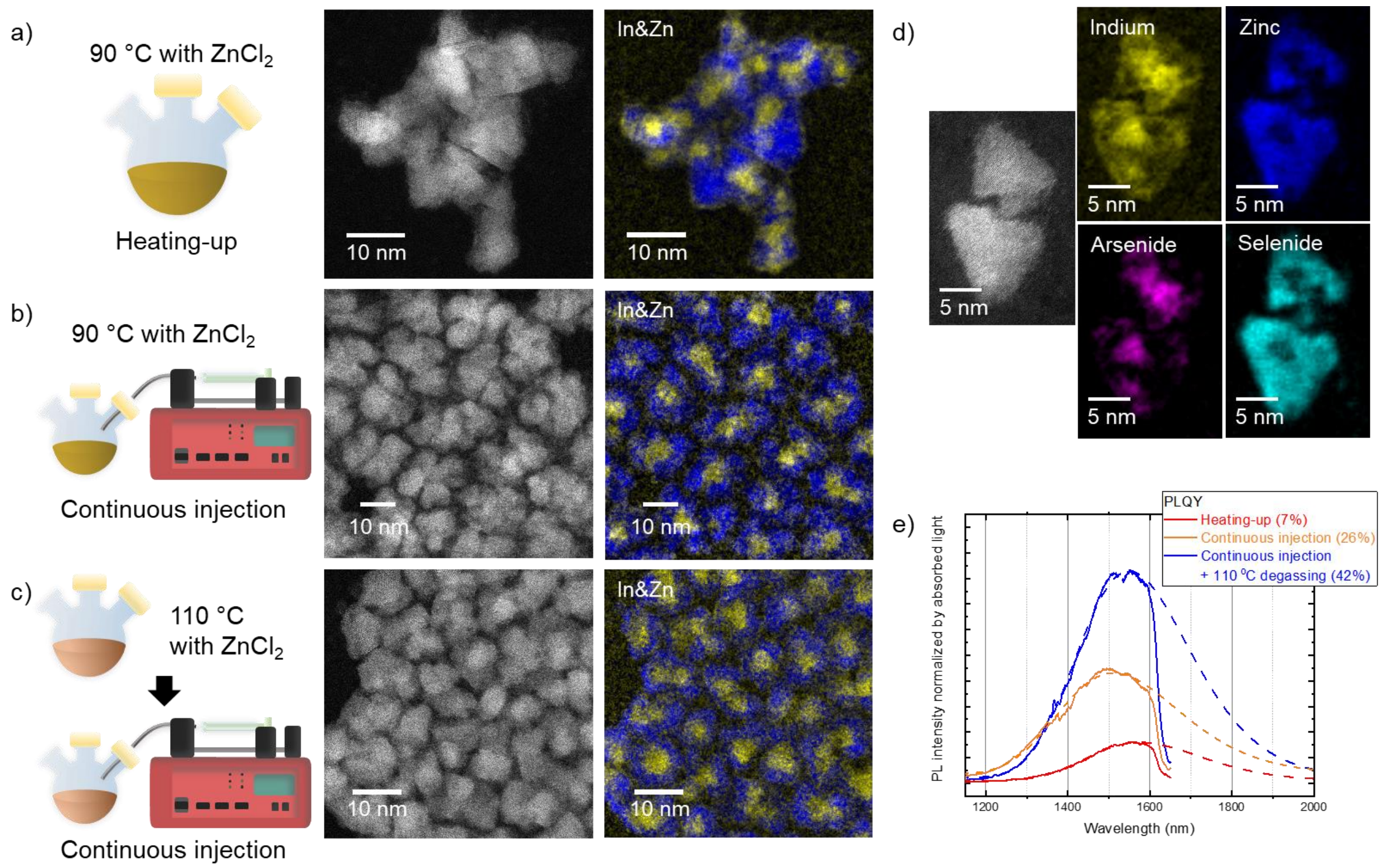

Fig. 1: Elucidation of the InAs/ZnSe core/shell growth mechanism. a HAADF-STEM image and corresponding EELS elemental map (In, yellow; Zn, blue) of InAs/ZnSe core/shell CQDs synthesized via a conventional heating-up method with $ZnCl_2$ degassed at 90 °C, showing severe aggregation. **b** HAADF-STEM image and EELS elemental map of core/shell CQDs synthesized under identical $ZnCl_2$ degassing conditions but with a continuous precursor injection protocol, revealing a pinwheel-like shell morphology with non-conformal ZnSe growth. **c** HAADF-STEM image and EELS elemental map of core/shell CQDs synthesized with $ZnCl_2$ degassed at 110 °C followed by continuous injection, yielding uniform tetrahedral or near-spherical morphologies. **d** EELS elemental maps of a representative CQD from the optimized synthesis (c), showing the spatial distribution of indium, zinc, arsenic, and selenium, with trace co-distribution of In and As in the shell region indicative of interfacial alloy formation. **e** PL spectra of InAs/ZnSe core/shell CQDs synthesized under the three conditions, with PLQY values of 7%, 26%, and 42% for heating-up, continuous injection, and 110 °C degassing with continuous injection, respectively.

5) The comparison of the EELS data in Fig 2a is only meaningful if the count rates that are used to display the contrast for the individual images is the same for both samples. In particular, this is vital for the oxygen spectra. Please provide a unified color map that explicitly shows that “a purple pixel of a certain intensity” means the same EELS count rate in both images

Author response to comment 5: We agree that the color intensities of independently normalized EELS maps cannot be compared quantitatively between samples. Therefore, we do not use the map contrast itself to compare the oxygen content. Instead, we added quantitative line-profile analyses reporting the oxygen atomic percentage for both samples using the same quantification procedure. These profiles show a localized enhancement of the oxygen signal at positions coinciding with the In-rich region only in the oxidized sample. The elemental maps were individually normalized solely to visualize the spatial distribution within each sample.

Description on page 7 of manuscript:

As shown in Fig. 2c, the EELS mapping of the pristine InAs/ZnSe CQDs reveals a distinct indium signal but lacks any localized oxygen signal in the CQDs. In contrast, the EELS mapping of indium and oxygen for the oxidized-core/shell CQDs in Fig. 2d reveals a localized enhancement of the oxygen signal at positions coinciding with the In-rich region, which is absent in the pristine sample. Although a high oxygen background signal is present in both samples due to the methanol treatment during TEM sample preparation as shown in Supplementary Fig. 7., the compositional profile clearly distinguishes this localized interfacial oxide layer, which is absent in the pristine sample.

6. Why was the In 3d-3/2 component not provided/analyzed in the same detail as the 5/2-component? This would have helped to improved the fit.

Author response to comment 6: The 3/2 component was initially omitted solely for visual clarity. We have now included the 3/2 component, which exhibits the same behavior and identical peak shifts as the 5/2 component.

7. Minor points:

Please provide the full survey spectra used in the XPS studies to indicate how relevant the selected binding energy regions were for the sample

Please provide the full Raman spectra from which the selections in Fig. 1e for made

Page 3: Please provide all spectral shifts in meV, not nm.

Fig 1a: Typo: “pristine

Author response to comment 7: We have included the full XPS survey spectra in the revised Supporting Information. Additionally, we have corrected the typographical error in Fig. 1a as requested. Finally, we have thoroughly reviewed the entire manuscript to ensure unit consistency and to correct any other potential typographical errors.

The full Raman spectra are included below for completeness; no additional Raman features were observed outside the InAs LO/TO region within the measured 150–500 cm^{-1} range.

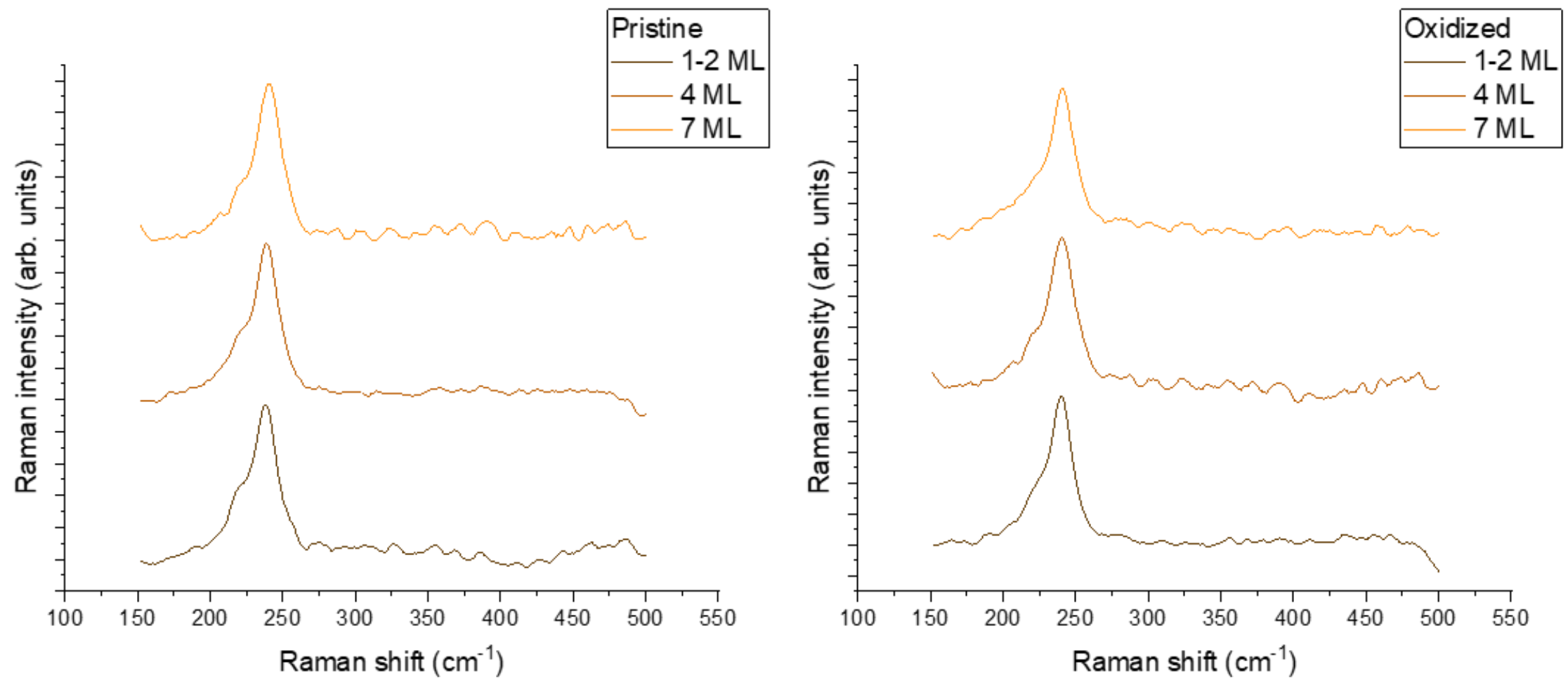

Response Figure 4. Full Raman spectra of InAs/ZnSe

Controlling the Shell Growth Mechanism and Interfacial Oxidation of InAs/ZnSe Quantum Dots for Shortwave Infrared Emission

Taewan Kim[§], Manuela De Franco[§], Miguel Dosil, Jae Taek Oh, Rajesh Bera, Marta Martos Valverde, Gaurav Kumar & Gerasimos Konstantatos[*]

REVIEWER COMMENTS

==
Reviewer 1 Comments to the Author
==

The revisions are satisfactory. I recommend the acceptance of this manuscript for publication at Nature Comm.

Author response: We thank the reviewer for their positive assessment of our revisions and for recommending the manuscript for publication.

==
Reviewer 2 Comments to the Author
==

The authors have addressed all my concerns and the manuscript has been substantially improved accordingly. I thus recommend the manuscript to be accepted for publication in Nature Communications.

Author response: We thank the reviewer for their positive assessment. We appreciate the reviewer's comments, which helped us substantially improve the manuscript, and we are pleased that all concerns have been satisfactorily addressed.

==
Reviewer 3 Comments to the Author
==

The authors have made substantial efforts to address my concerns. Especially the extra work for establishing the robustness of their PLQY measurements and the streamlined XPS vs. XRD experiments are noteworthy. I can now recommend the manuscript for publication in Nature Comm.

Author response: We thank the reviewer for their positive assessment and recommendation for publication. We particularly appreciate the reviewer's recognition of the additional work undertaken to establish the robustness of the PLQY measurements and to strengthen the XPS and XRD analyses.
